# Genome-wide Association Studies of Retinal Vessel Tortuosity Identify Numerous Novel Loci Revealing Genes and Pathways Associated With Ocular and Cardiometabolic Diseases

**DOI:** 10.1016/j.xops.2023.100288

**Published:** 2023-02-16

**Authors:** Mattia Tomasoni, Michael Johannes Beyeler, Sofia Ortin Vela, Ninon Mounier, Eleonora Porcu, Tanguy Corre, Daniel Krefl, Alexander Luke Button, Hana Abouzeid, Konstantinidis Lazaros, Murielle Bochud, Reinier Schlingemann, Ciara Bergin, Sven Bergmann

**Affiliations:** 1 Department of Computational Biology, University of Lausanne, Lausanne, Switzerland; 2 Swiss Institute of Bioinformatics, Lausanne, Switzerland; 3 Jules-Gonin Eye Hospital, Lausanne, Switzerland; 4 Center for Primary Care and Public Health (Unisanté), University of Lausanne, Lausanne, Switzerland; 5 Center for Integrative Genomics, University of Lausanne, Lausanne, Switzerland; 6 Division of Ophthalmology, Geneva University Hospitals, Geneva, Switzerland; 7 Clinical Eye Research Center Memorial Adolphe de Rothschild, Geneva, Switzerland; 8 Department of Integrative Biomedical Sciences, University of Cape Town, Cape Town, South Africa; 9 Department of Ophthalmology, Amsterdam University Medical Centres, Amsterdam, The Netherlands

**Keywords:** GWAS, Retina, Microvasculature, Tortuosity, Mendelian randomization

## Abstract

**Purpose:**

To identify novel susceptibility loci for retinal vascular tortuosity, to better understand the molecular mechanisms modulating this trait, and reveal causal relationships with diseases and their risk factors.

**Design:**

Genome-wide Association Studies (GWAS) of vascular tortuosity of retinal arteries and veins followed by replication meta-analysis and Mendelian randomization (MR).

**Participants:**

We analyzed 116 639 fundus images of suitable quality from 63 662 participants from 3 cohorts, namely the UK Biobank (n = 62 751), the *Swiss Kidney Project on Genes in Hypertension* (n = 397), and *OphtalmoLaus* (n = 512).

**Methods:**

Using a fully automated retina image processing pipeline to annotate vessels and a deep learning algorithm to determine the vessel type, we computed the median arterial, venous and combined vessel tortuosity measured by the *distance factor* (the length of a vessel segment over its chord length), as well as by 6 alternative measures that integrate over vessel curvature. We then performed the largest GWAS of these traits to date and assessed gene set enrichment using the novel high-precision statistical method *PascalX*.

**Main Outcome Measure:**

We evaluated the genetic association of retinal tortuosity, measured by the distance factor.

**Results:**

Higher retinal tortuosity was significantly associated with higher incidence of angina, myocardial infarction, stroke, deep vein thrombosis, and hypertension. We identified 175 significantly associated genetic loci in the UK Biobank; 173 of these were novel and 4 replicated in our second, much smaller, metacohort. We estimated heritability at ∼25% using linkage disequilibrium score regression. Vessel type specific GWAS revealed 116 loci for arteries and 63 for veins. Genes with significant association signals included *COL4A2*, *ACTN4*, *LGALS4*, *LGALS7*, *LGALS7B*, *TNS1*, *MAP4K1*, *EIF3K*, *CAPN12*, *ECH1*, and *SYNPO2*. These tortuosity genes were overexpressed in arteries and heart muscle and linked to pathways related to the structural properties of the vasculature. We demonstrated that retinal tortuosity loci served pleiotropic functions as cardiometabolic disease variants and risk factors. Concordantly, MR revealed causal effects between tortuosity, body mass index, and low-density lipoprotein.

**Conclusions:**

Several alleles associated with retinal vessel tortuosity suggest a common genetic architecture of this trait with ocular diseases (glaucoma, myopia), cardiovascular diseases, and metabolic syndrome. Our results shed new light on the genetics of vascular diseases and their pathomechanisms and highlight how GWASs and heritability can be used to improve phenotype extraction from high-dimensional data, such as images.

**Financial Disclosure(s):**

The author(s) have no proprietary or commercial interest in any materials discussed in this article.

Cardiovascular diseases (CVD) are the leading cause of death in developed countries^1^, ^2^, ^3^ and a major societal health burden. Though several risk factors for CVD development, such as age, smoking, and hypertension, have been firmly established, the degree of importance of vascular properties as risk factors is unclear. Retinal fundus photos allow noninvasive in vivo assessment of the vascular system of the superficial inner retina, i.e., the central and branch veins and arteries plus the venules and arterioles. These vessels are composed of tightly sealed endothelial cells (ECs) forming the inner blood-retina barrier, encased by smooth muscle cells (SMCs) forming the vessel wall.^4^^,^^5^ Automatic segmentation of retinal vessels in fundus images is well established, and computer-aided image analysis started entering clinical care to screen and diagnose ocular and systemic diseases.^6^ In diabetes, for example, hyperglycemia induces damage to the ECs and pericytes of the inner blood-retina barrier contributing to retinal edema and hemorrhage.^7^

Pathological changes in the retinal vessels often coincide with those in the microvasculature of other organs and may precede the progression of systemic vascular diseases. The retinal vasculature can provide insights into neurodegenerative diseases, such as Alzheimer’s, Parkinson’s, and vascular dementia.^8^, ^9^, ^10^, ^11^, ^12^ In addition, abnormalities in retinal parameters, such as vascular calibers and tortuosity, are of diagnostic value for systemic diseases, including increased risk of diabetes,^13^, ^14^, ^15^ obesity,^16^ and CVD^17^^,^^18^ (such as stroke,^19^, ^20^, ^21^, ^22^ coronary heart disease,^23^ peripheral artery disease,^24^ hypertension,^21^^,^^25^, ^26^, ^27^, ^28^, ^29^, ^30^, ^31^, ^32^, ^33^ atherosclerosis,^19^^,^^21^^,^^34^ myocardial infarction,^35^^,^^36^ and nephropathies^37^^,^^38^).

In recent years, genome-wide association studies (GWAS) have been used to link genes with phenotypes extracted from fundus images, such as vessel size,^39^^,^^40^ optic disc morphology,^41^^,^^42^ vascular density,^43^ fractal dimensions,^43^ and vessel tortuosity.^44^ The diameter of the retinal microvasculature was associated with genes *TEAD1*, *TSPAN10*, *GNB3*, and *OCA2*.^39^ A recently published study^43^ on vascular density and fractal dimensions reported 7 and 13 single nucleotide polymorphisms (SNPs) associated with these traits respectively, including *OCA2*, *MEF2C*, and *GNB3*. Retinal vessel tortuosity has been associated with SNPs that map to the genes *ACTN4* and *COL4A2*.^44^ Tortuosity of the vasculature was reported in the context of coronary artery disease (CAD)^44^ and connective tissue disease.^45^ These results demonstrated that GWAS on retinal traits extracted at a single time point can reveal genes with a potential role in modulating vascular properties and related pathomechanisms.

Here, we report the results of the largest GWAS on vessel tortuosity to date using images and genotypes from 62 751 subjects in the UK Biobank (UKBB) and from 397 and 512 subjects of the much smaller, yet independent, population-based cohorts, the *Swiss Kidney Project on Genes in Hypertension* (SKIPOGH)^46^^,^^47^ and *OphtalmoLaus*.^48^ Our study was motivated by the clinical relevance of this trait to diseases^9^^,^^13^^,^^28^^,^^45^^,^^49^^,^^50^ and by the fact that significant associations were already reported in much smaller sample sizes,^44^ making further discoveries likely. We constructed an automated image analysis pipeline to extract retinal tortuosity from these data as a biomarker. We report the correlation with patient records, SNPs, genes, pathways (set of genes), tissue expression, pathomechanisms, and causal effects associated with this biomarker. Our findings advance the understanding of the molecular players and mechanisms contributing to retinal vessel morphology, which may be important also for other vasculatures and associated diseases.

## Methods

### Data: Genotypes, Phenotypes, and Fundus Images

The UKBB is a population-based cohort of approximately 488 000 subjects with rich, longitudinal phenotypic data and a median 10-year follow-up.^51^^,^^52^ We analyzed 173 837 standard retinal 45° color fundus images from 84 825 individuals, captured using a Topcon Triton 3D OCT 1000. Genotyping was performed on Axiom arrays for a total of 805 426 markers, from which approximately 96 million genotypes were imputed. We used the subset of 15 599 830 SNPs that had been assigned an rsID. We performed an additional quality control (QC) step by filtering out SNPs with minor allele frequency < 5 × 10^−4^. Our choice of low minor allele frequency cut-off was motivated by the large power of the UKBB. With our sample size of 62 751 of subjects after QC (see below), we still expect about 30 subjects to have ≥ 1 minor allele, so the effect size estimate is still reasonably robust. Finally, we applied a filtering procedure^53^ to remove SNPs with imputation quality < 0.3. In addition to genomic information, the UKBB also provided us with phenotypic information from the patient records, particularly with diagnosis dates for: type-2 diabetes, angina, myocardial infarction, deep vein thrombosis, stroke, hypertension and smoking status. Age, sex, and principal components of genotypes were used to correct for biases in the genetic associations.

**Figure 1:**
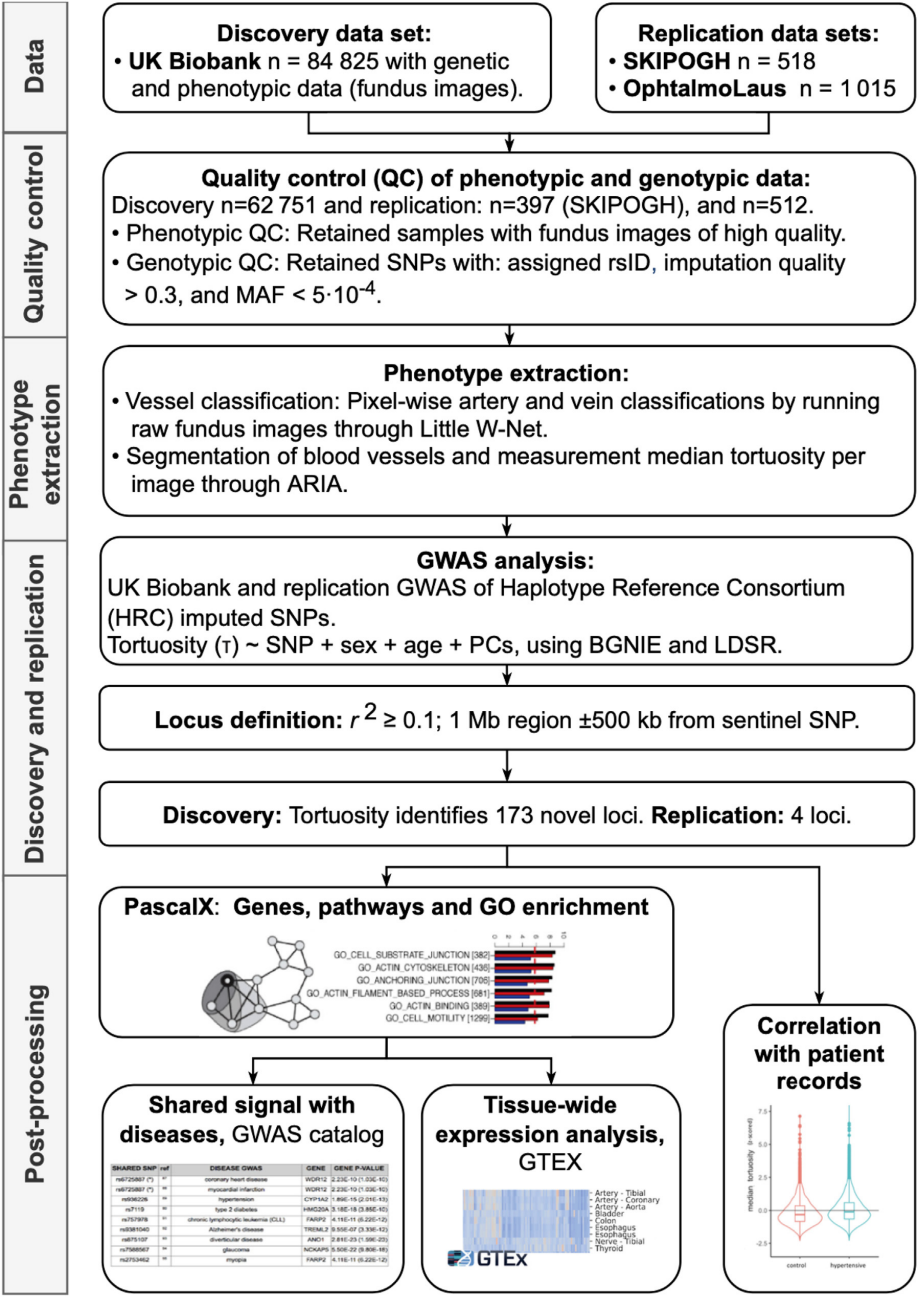
Pipeline and results. Relevant phenotypes, genotypes, and fundus images were collected from the UK Biobank, *OphtalmoLaus,* and the *Swiss Kidney Project on Genes in Hypertension (*SKIPOGH). After quality control, the images were processed by deep learning, classifying arteries and veins. A range of tortuosity measures were then calculated which provided the phenotypes for the genome-wide association studies (GWASs). The primary results were 173 novel genetic trait loci. These associations include signals which were shared between retinal tortuosity and several diseases (metabolic syndrome and cardiovascular diseases). Their aggregation on annotated gene-sets identified relevant pathways and gene ontology (GO) terms. Tissue-wide expression analysis revealed expression in the arteries and heart. Correlation analysis revealed associations between retinal tortuosity and cardiometabolic diseases. LDSR = linkage disequilibrium score regression; MAF = minor allele frequency; PC = principle component; SNPs = single nucleotide polymorphisms.

We performed replication via a meta-analysis of 2 independent, population-based cohorts: SKIPOGH^46^^,^^47^ and *OphtalmoLaus*.^48^ The SKIPOGH is a family-based, cross-sectional study exploring the role of genes and kidney hemodynamics in blood pressure regulation and kidney function in the general population, comprising 1054 genotyped individuals. One thousand three hundred fifty-two retinal fundus images were available from 518 participants. The genotyping was performed with the Illumina Omni 2.5 chip. *OphtalmoLaus* is a substudy of *Cohorte Lausannoise* (*CoLaus*), a population-based cohort comprising 6188 genotyped individuals. Seven thousand two hundred fifty-two fundus images were available from 1015 subjects. *CoLaus* has as its objective to investigate the epidemiology and genetic determinants of CVD risk factors and metabolic syndrome; participants were phenotyped accordingly. The genotyping was performed using the 500K Affymetrix chip technology. Like in the UKBB, in both Swiss cohorts retinal fundus images were captured using Topcon Triton devices. Genotype imputation for SKIPOGH and *CoLaus* was performed using Minimac 3 as algorithm and version 1.1 from the Haplotype Reference Consortium (http://www.haplotype-reference-consortium.org) as reference panel. For an overview of our pipeline see Figure 1.

### Automated Analysis of Color Fundus Images and QC

We extended the software ARIA^54^ to perform batch segmentation and positional annotation of blood vessels, using the default parameters.^55^ The exclusion criteria were based on upper and lower thresholds on the total length of the vasculature and on the number of vessels (Supplemental Text 1). Roughly 2 of 3 images passed this strict QC (116 639 out of 173 837 in the UKBB). Based on ARIA's vessel annotations, we calculated a tortuosity measure known as the *distance factor* (DF),^56^ defined as:

$$DF=\frac{s\left(C\right)}{chord\left(C\right)}$$

where the total vessel length, s(C), is divided by the Euclidean distance between the vessel segment endpoints, chord(C). Distance factor is referred to in a recent review as the arc over chord ratio.^57^ In addition to DF, we also calculated 6 other tortuosity phenotypes based on alternative measures using integrals over the curvature along the vessel (Supplemental Text 2).

We phenotyped each individual by calculating median retinal tortuosities, then averaging the values derived from 1 image of the left and 1 from the right eye, when available. If only 1 retinal image was available we used the value of this image. In the few cases where multiple images were available for the same eye, we only considered 1 image from the earliest time point (for the resulting distribution, refer to Supplemental Text 3).

### Deep Learning Classification of Arteries and Veins

We calculated pixel-wise artery and vein classifications using the deep learning algorithm *Little W-Net*.^58^ For each vessel segment recognized by ARIA, we used the difference between pixels classified as arterial and venous as a score that was required to be positive or negative for the segment to be annotated as artery or vein, respectively. On a set of 44 images, manually annotated by an ophthalmologist (H.A.), we obtained an area under the curve of 0.93 and an accuracy of 0.88. Thus, we performed vessel type classification for the entire set of retinal fundus images; computing artery- and vein-specific tortuosity values (Supplemental Text 4).

### Genome-wide Association Analyses

We ran genetic association studies on tortuosity of arteries, of veins, and combining both vessel types (from UKBB color fundus images [CFIs]). We used BGENIE,^59^ applying linear regression to confounder-corrected, quantile-quantile normalized, and retinal vessel tortuosity on the genotypes of the matching subjects imputed to a panel of approximately 15 million genetic variants. In order to account for confounding effects,^60^ the following variables were provided as covariates, as usual in GWAS: age, sex, and principle component (PC) of the genotypes (we considered only PCs with a significant correlation to tortuosity, namely 1, 2, 5, 6, 7, 8, 16, 17, and 18). A sensitivity analysis controlling for additional covariates, including age-squared, spherical power, smoking, hypertension, diabetes, eye-related diseases and conditions, assessment-center, and genotyping array, indicated only minor impact on the significant association *P*-values (Supplemental Text 14). We considered SNPs to be nominally significant if their *P*-value was below the classical Bonferroni threshold of 5 × 10^−8^ (i.e., correcting for an estimated 1 million of independent SNPs). A list of independent SNP was obtained by performing linkage disequilibrium (LD) pruning using the LD pair function of the R package LD linkR.^61^ Two SNPs were considered independent if they had LD *r*^*2*^ < 0.1 or were > 500 000. bases apart (Supplemental Dataset 1).

### Replication Metacohort

As the SKIPOGH cohort includes subjects with a high degree of relatedness, we used the EMMAX function of the Efficient and Parallelizable Association Container Toolbox (EPACTS) software^62^ and the kinship matrix in the model to account for family structure. We also included the recruitment center as a covariable. For the GWAS on the *OphtalmoLaus* cohort, we used the same parameters and tools as for the discovery cohort. Results from SKIPOGH and *OphtalmoLaus* were meta-analyzed using an inverse-variance weighting scheme for the respective effect sizes. Due to the small sample size of the replication cohort, we only attempted replication for the SNPs and genes that were significant in the discovery cohort.

### Heritability Estimates

We used LD Score Regression^63^ to estimate the SNP-based heritability of our tortuosity measures.

### Novel Method for Gene-Based Tests

We used *PascalX*,^64^ a novel high-precision pathway scoring algorithm that we developed, building on our *Pascal*^65^ tool, to aggregate SNP-wise summary statistics into gene scores using a sum of χ^2^ statistics: *PascalX* takes into account LD by effectively transforming the sum of χ^2^ from all SNPs within the gene window into a new basis of independent “Eigen-SNPs” corresponding to a weighted sum of χ^2^ statistics. Using multiple-precision arithmetics, *PascalX* computes the corresponding null cumulative probability distribution to essentially arbitrary precision, while other tools usually only approximate the underlying distribution. We thus computed *P*-values up to a precision of 10^−100^, allowing for accurate scoring of genes with contributions from extremely significant SNPs, which become increasingly frequent in highly powered GWASs such as this one.

We used the following configurations: We computed gene scores from SNPs lying within a window of 50 kb before the transcription start site and 50 kb after the transcript end. The annotation of the gene positions was based on the Genome Reference Consortium Human genome build 37 (GRCh37/hg19) downloaded from the Ensembl biomart^66^; we considered only protein-coding and lincRNA genes. The reference panel from the UK10K project^67^ was used to estimate the SNP-SNP correlations (LD effects). *PascalX* uncovered 265 significant genes (after Bonferroni correction for 25 489 gene-based tests *P* < 0.05/25 489 ≃ 2.0 × 10^−6^).

### Gene Set Enrichment

We used *PascalX*^64^ to compute gene set enrichment scores based on ranking derived from the gene-based tests. As a large number of genes have inflated *P*-values in highly powered GWASs, this ranking approach was more conservative. We first computed scores for 2868 canonical pathways (BioCarta, Kyoto Encyclopedia of Genes and Genomes (KEGG), protein interaction database (PID), Reactome, and WikiPathways), then extended our analysis to the 31 120 pathways in MSigDB (version 7.2).^68^ To adjust for statistical dependence and coexpression, genes that are < 100 kb apart were “fused” (i.e., considered as single entities termed “fusion genes”^65^).

### Tissue-wide Gene Expression Analysis

We performed tissue-wide gene expression analysis using *PascalX*^64^ on the whole GTEx^69^ (version 8) dataset, comprising 54 tissues. We defined gene sets based on the significant genes from each of our 3 GWAS on DF tortuosity (artery, vein, and combined). *PascalX* was used to perform an enrichment analysis that indicated whether these sets were over-expressed in any particular tissue. *PascalX* corrected for the co-expression of gene subclusters within each gene set by merging nearby genes to fusion genes. We computed the fusion genes expression values in transcripts per kilobase million from the raw read counts. These values values were made uniform via ranking, transformed to χ^2^-distributed random variables, summed, and tested against a χ^2^ distribution with as many degrees of freedom as there were “fusion genes” in each set. We applied a Bonferroni threshold: *P* = 0.05/54 = 9.2 × 10^−4^.

### Shared Genetic Signal With Disease

We computed the overlap between DF tortuosity SNPs (from the combined-vessel GWAS) and disease-related SNPs. To this end, we first identified which of the independent SNPs in the combined-vessel GWAS were listed in the GWAS Catalog.^70^ We then extended this analysis by considering DF tortuosity SNPs in LD (*r*^*2*^ > 0.8) with disease-related SNPs in the GWAS Catalog.

### Mendelian Randomization Analysis

We performed 2-sample bidirectional Mendelian randomization (MR)^71^^,^^72^ to search for evidence of causal effects between DF tortuosity (from the combined-vessel GWAS) and the following traits: body mass index (BMI), CAD, systolic blood pressure, and lipid traits, namely high-density lipoprotein, low-density lipoprotein (LDL), total cholesterol, and triglycerides. For each trait, we used independent (*r*^*2*^ < 0.01) significant (*P* < 5 × 10^−8^) SNPs as instrumental variables. All summary statistics (estimated univariate effect size and standard error) originated from the most recent meta-analyses (not including UKBB individuals) and were downloaded from the publicly available National Institutes of Health Genome-wide Repository of Associations between SNPs and Phenotypes.^73^ We only used SNPs on autosomal chromosomes available in the UK10K reference panel,^67^ which allowed us to estimate the LD among these SNPs and prune them. We removed strand ambiguous SNPs. Causal estimates were based on the inverse variance weighted method^74^ and calculated using the MR R package.^75^

### Code Availability

The code used to measure the tortuosity phenotypes is available at: https://github.com/BergmannLab/Retina-tortuosity.

### Ethics Approval

The UKBB has obtained Research Tissue Bank approval from its ethics committee that covers our use of the resource. The UKBB Research Ethics Committee approval number is 16/NW/0274. *OphtalmoLaus* obtained ethics approval from *La Commission cantonale d'éthique de la recherche sur l'être humain* (project PB_2019-00168). The same commission approved ethics for SKIPOGH (Protocols 92/07 and 303/12). All 3 studies adhere to the Declaration of Helsinki and obtained informed consent from all subjects.

## Results

### Baseline Characteristics and Tortuosity Quantification

Following QC measures, we analyzed 116 639 images from 62 751 subjects of the UKBB (mean ± standard deviation age = 56 ± 8 years; 35 098 females at birth [54%]; 4618 smokers [7%]). We analyzed 1352 images from 379 subjects of the SKIPOGH cohort (mean ± standard deviation age = 48 ± 16 years; 211 females [53%]; 107 smokers [27%]). We analyzed 7254 images from 512 subjects of the *OphtalmoLaus* cohort (mean ± standard deviation age = 51 ± 10 years; 270 females [53%]). Baseline characteristics and disease prevalence are presented in Supplemental Text 6. For an overview of our pipeline see Figure 1. Note that we did not explicitly exclude subjects with retinal diseases or other ocular conditions from the dataset, but that images from such subjects often did not pass our QC standards (Supplemental Text 1).

The distributions of DF tortuosity were similar across cohorts: long-tailed, left-skewed, with means ranging from 1.030 (UKBB) to 1.034 (*OphtalmoLaus*). Distance factor was higher in the elderly population (Cohen’s d = 0.49, *P =* 1 × 10^−195^) and in women (Cohen’s d = 0.049, *P =* 9 × 10^−10^). Overall, DF was higher in veins (Cohen’s d = 0.13, *P* = 9 × 10^−142^). For details about the stratified analysis of the DF phenotype in the UKBB see Supplemental Text 3.

We extracted 6 additional tortuosity measures based on alternative mathematical definitions. Correlations analysis and dimensionality reduction in terms of principle components showed that the DF is most similar to the path integral of the squared curvature (τ_3_) and least similar to the path integral of the curvature (τ_2_). The other alternative measures (τ_4–7_) were similar to each other, very different from τ_2_ and of intermediate similarity to the DF and τ_3_ (Supplemental Text 2).

### Vessel Tortuosity Correlates With Disease Status

We found that the DF tortuosity of arteries was associated with hypertension (*beta =* 0.19, *P =* 3 × 10^−56^) and angina (*beta =* 0.09, *P =* 6 × 10^−4^), but not with myocardial infarction, stroke, or deep vein thrombosis. In the case of veins, the DF was significantly associated with hypertension (*beta =* 0.25, *P =* 7 × 10^−99^), angina (*beta =* 0.18, *P =* 2 × 10^−10^), myocardial infarction (*beta =* 0.12, *P* = 2 × 10^−4^), stroke (*beta =* 0.16, *P =* 5 × 10^−5^), and deep vein thrombosis (*beta =* 0.11, *P* = 5 × 10^−4^). For predictive power over disease status, see Supplemental Text 7.

### Vessel Tortuosity GWASs Identify 173 Novel Loci

**Figure 2:**
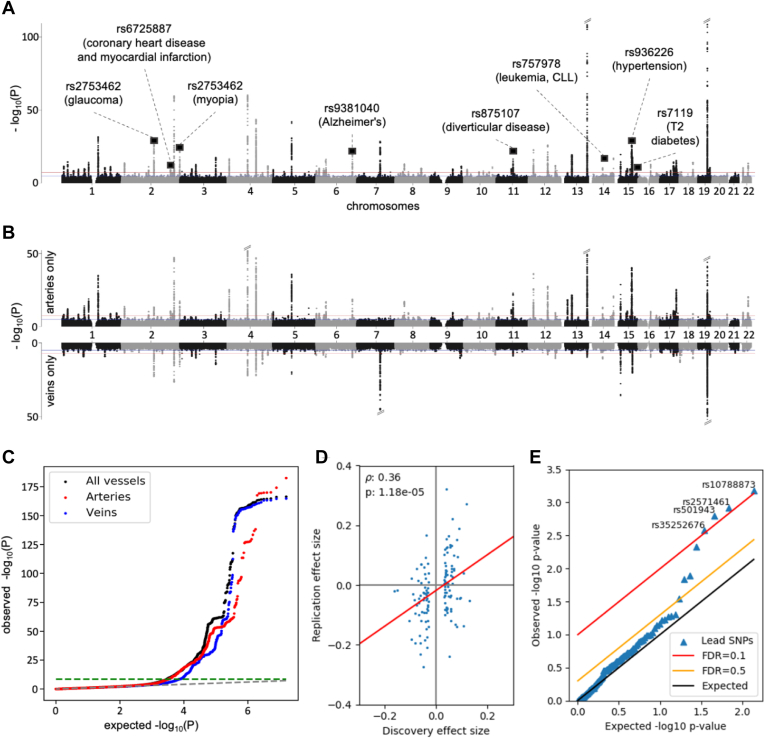
Single nucleotide polymorphism (SNP) *P*-values and effects. **A,** Manhattan plot of Genome-Wide Association Study (GWAS) of retinal vessel tortuosity, combining all vessel types (both arteries and veins). The red line indicates the genome-wide significance level after Bonferroni correction (*P* = 5 × 10^−8^). Oblique dashes on top of peaks mark extremely significant *P*-values that have been cropped. Squares mark the position of disease SNPs (Table 4). The trait was corrected for phenotypic variables which showed a statistically significant association, i.e.: age, sex, and a subset of principal components of genotypes. **B,** Manhattan plots of the vessels-specific GWAS (artery-specific on top, vein-specific at the bottom). Confounder correction, significance level and cropping of extremely significant *P*-values as in the (**A**). **C,** GWAS q-q plot: arteries in red, veins in blue, combined-vessels signal in black; the genome-wide significance level is represented as a green dashed line. **D,** Statistically significant correlation between the measured effect sizes in the discovery cohort (UK Biobank [UKBB], n = 62 751) and replication metacohort (the *Swiss Kidney Project on Genes in Hypertension* plus *OphtalmoLaus*, n = 911). We considered all lead (independent) SNPs in the UKBB. We tested all 136 SNPs with matching rsIDs in the replication metacohort except 1 censored outlier (rs187691758), 89 of which had the same sign of their effect size estimate in the UKBB. The resulting Pearson correlation is *r =* 0.36; *P =* 1.18 × 10^−5^. **E,** Benjamini-Hochberg procedure on discovery lead SNPs from the UKBB yields 4 hits in the replication cohort using false discovery rate (FDR) = 0.2.

We identified 7072 significantly associated SNPs in the combined-vessel GWAS on DF tortuosity in the UKBB (Supplemental Dataset 4A). The vessel type specific GWAS resulted in 6563 significantly associated SNPs for arteries, and 2896 SNPs for veins when using a Bonferroni threshold of 5 × 10^−8^ (Supplemental Dataset 4B, C). We applied LD pruning, identifying 128 independent loci in the combined-vessel GWAS, 116 in the artery-specific GWAS, and 63 in the vein-specific GWAS. Accounting for overlap between these sets (Supplemental Text 9), we obtained a total of 175 independent lead SNPs (Figure 2A–C). The top 10 SNPs are listed in Table 1, ordered by significance (for complete listings, see Supplemental Dataset 1). Among the significantly associated variants, rs1808382 and rs7991229 had been previously reported^44^ (Supplemental Text 8), whereas the remaining 173 independent lead SNPs represented novel loci associated (Supplemental Dataset 5).

**Table 1:** Top Retinal Tortuosity SNPs

Chr	SNP	EA	RA	Freq	beta	−log_10_*P*	GWAS Type
13	rs9559797	G	C	0.580	−0.162	182.4	artery and vein
19	rs16972767	G	A	0.473	−0.150	164.6	artery and vein
4	rs17008193	T	C	0.403	−0.089	55.0	artery and vein
7	rs187691758	A	G	0.005	0.627	53.2	vein
4	rs12506823	G	A	0.406	0.083	47.9	artery and vein
2	rs2571461	T	G	0.601	−0.082	47.1	artery and vein
15	rs12913832	A	G	0.744	0.080	43.6	artery and vein
12	rs11045245	A	G	0.375	0.073	37.3	artery
5	rs784420	A	G	0.281	0.078	37.0	artery and vein
4	rs11727963	G	A	0.166	0.092	35.1	artery

The 10 most significant distance factor tortuosity SNPs, ordered by *P*-value. For full results, refer to the list of 175 independent lead SNPs in Supplemental Dataset 1.

beta = effect size estimate; Chr = chromosome; EA = effect allele; freq = allele frequency of effect allele; GWAS = genome-wide association study; GWAS type = vessel type to which the signal applied; RA = reference allele; SNP = rsIDs of the single nucleotide polymorphism; −log_10_*P* = normalized *P*-value in the discovery cohort.

### Heritability of DF is Larger than for Other Tortuosity Measures

The SNP-based heritability differed substantially across tortuosity measures, with DF receiving the highest estimate (h^2^_SNP_ = 0.25, standard error [SE] = 0.025). This was approximately twice the heritability estimate of the 6 alternative curvature-based measures (0.11 ≤ h^2^_SNP_ ≤ 0.13, 0.011 ≤ SE ≤ 0.012, Supplemental Text 2). We did not observe any significant genomic inflation (Table 2). Heritability also varied depending on vessel type (h^2^_SNP_ = 0.23 [SE = 0.020] for arteries, and h^2^_SNP_ = 0.15 [SE = 0.021] for veins). The distribution of the DF phenotype for each vessel type is shown in Supplemental Text 3.

**Table 2:** SNP-Based Heritability

GWAS Type	h2SNP	lambda GC	Mean Chi2	Intercept	Ratio
combined-vessel	0.25 (0.025)	1.14	1.31	1.01 (0.01)	0.03 (0.03)
artery	0.23 (0.020)	1.12	1.27	1.00 (0.01)	< 0
vein	0.15 (0.021)	1.10	1.18	1.00 (0.01)	< 0

h^2^_SNP_ = portion of phenotypic variance cumulatively explained by the SNPs; GWAS = genome-wide association study; intercept = linkage disequilibrium score regression intercept (values close to 1 indicates little influence of confounders, mostly of population stratification); lambda GC = inflation, measure of the effect of confounding and polygenicity acting on the trait; ratio = ratio of the proportion of the inflation in the mean Chi^2^ that is not due to polygenicity (a ratio close to, or smaller than, 0 is desirable as it indicates low inflation from population stratification); SNP = single nucleotide polymorphism. Standard error are given in parentheses.

### Replication of Lead SNPs and Genes in a Small Metacohort

**Figure 3:**
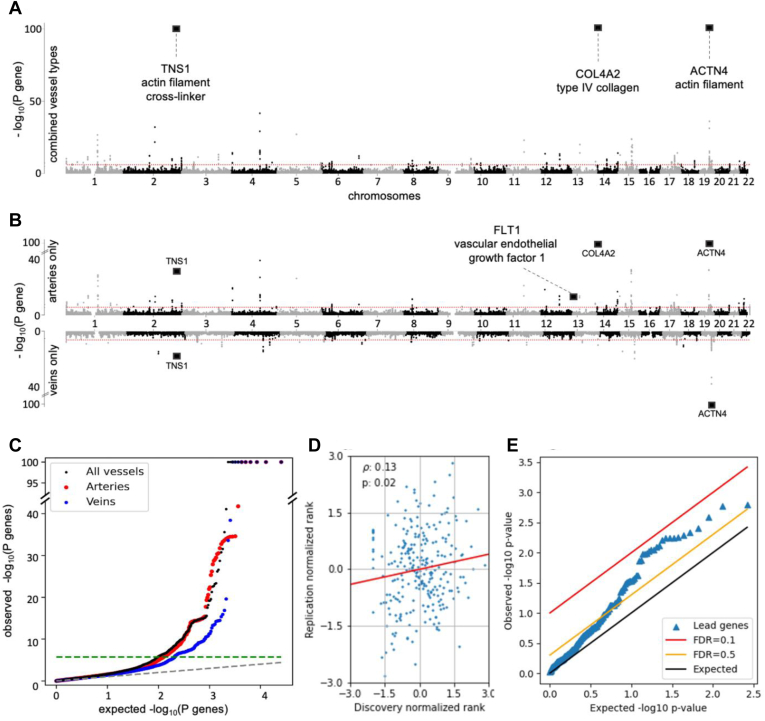
Gene *P*-values and replication scores. **A,** Gene-based Manhattan plot of retinal vessel tortuosity, combining all vessel types (both arteries and veins). Two hundred three genes were significant in arteries, 123 in genes, and 265 when combining the vessel types. Gene-based tests were computed by *PascalX*.^64^ The red line indicates the genome-wide significance level after Bonferroni correction (*P =* 5 × 10^−8^). Squares mark the position of particularly relevant genes (see corresponding Results section). **B,** Gene-based Manhattan plots of the vessels-specific genome-wide association study (artery-specific on top, vein-specific at the bottom). **C,** q-q plot of gene *P*-values: arteries in red, veins in blue, combined-vessel signal in black; the genome-wide significance level is represented as a green dashed line. **D,** Statistically significant correlation between q-q normalized genes’ *P*-values in the discovery (UK Biobank) and in the replication metacohort (the *Swiss Kidney Project on Genes in Hypertension* + *OphtalmoLaus*). Only genes that were significant in the discovery cohort were considered. The resulting Pearson correlation is *r =* 0.13 (*P =* 0.02). **E,** Benjamini-Hochberg procedure replicates 58 hits at false discovery rate (FDR) = 0.5 in the replication metacohort. We used a candidate approach, meaning only genes that were significant in the discovery cohort were considered.

The sample size of the replication metacohort (n = 909) is too low to replicate any of our discoveries with a fixed Bonferroni *P*-value threshold to correct for multiple hypotheses testing. We therefore used the well-established Benjamini–Hochberg procedure,^76^ which fixes a false discovery rate (FDR), corresponding to a variable threshold that is less stringent for SNPs with lower rank. With this procedure, for FDR = 0.1 (so expecting 1 in 10 positives to be false) we replicated 4 SNPs (rs10788873, rs2571461, rs501943, and rs35252676, indicated in Fig 2E) and at FDR = 0.5, 4 additional SNPs replicate. At FDR = 0.05 we could not replicate any of our hits. For genes, we found that 58 replicated at FDR = 0.5 but none at FDR = 0.1. Clearly, our replication metacohort lacks power, but many candidate SNPs, and even more so candidate genes, have more significant *P*-values than expected. Consistently, we observed a Pearson correlation of *r =* 0.36 (*P =* 1.18 × 10^−5^) between the SNP effect size estimates in the 2 studies (Fig 2D and Supplemental Text 5), and *r* = 0.13 (*P* = 0.02) between normalized gene ranks (Fig 3D).

### Tortuosity Genes and Pathways Affect Vascular Tissue Remodeling and Angiogenesis

Mapping the SNP-wise association signals onto genes (Methods), we identified 265 significant genes in the discovery GWAS combining vessel types, 203 in the artery-specific GWAS, and 123 in the vein-specific GWAS. Accounting for overlap between these sets (Supplemental Text 9), we obtained a total of 312 genes (Fig 3A–C). Top genes are reported in Table 3 (for a complete listing, see Supplemental Dataset 6A–C). Among those, we replicate the 3 genes in 2 independent loci (*ACTN4*/*CAPN12*, *COL4A2*) that were found in a previous GWAS study on tortuosity.^44^ A large fraction of these genes carried annotations related to vessel integrity, vascular tissue remodeling and angiogenesis. Specifically, we identified a cluster of highly significant genes on chromosome 19, including *ACTN4* (related to actin filament bundling), *TNS1* (cross-linking of actin filaments), and *CAPN12* (involved in structural integrity to blood vessel walls). This locus also included 3 genes involved in adhesion to the connective tissue^77^: *LGALS7*, *LGALS7B,* and *LGALS4*. We also replicated the highly significant association of tortuosity with 2 type IV collagen genes, *COL4A2* and *COL4A1*,^44^ the latter of which has already been associated with familial retinal arteriolar tortuosity.^78^ *SYNPO2*, related to actin polymerization, vascular injury,^79^ and ocular growth,^80^ also received a highly significant association. Finally, among the artery-specific genes, we found *FLT1* coding for VEGFR1, which plays a role in vessel formation and vascular biology^81^ (see Discussion for further details and interpretation of these results).

**Table 3:** Top Retinal Tortuosity Genes

Gene	Chr	Base Pair	−log_10_*P* Combined	−log_10_*P* Artery	−log_10_*P* Vein
*ACTN4*	19	39 138 289	> 100	> 100	> 100
*CAPN12*	19	39 220 827	> 100	> 100	> 100
*EIF3K*	19	39 109 735	> 100	> 100	> 100
*LGALS7*	19	39 261 611	> 100	> 100	> 100
*LGALS7B*	19	39 279 851	> 100	> 100	> 100
*COL4A2*	13	110 958 159	> 100	> 100	9.5
*LGALS4*	19	39 292 311	> 100	34.5	> 100
*MAP4K1*	19	39 078 281	> 100	34.3	> 100
*TNS1*	2	218 664 512	> 100	32.7	16.9
*ECH1*	19	39 306 062	> 100	15.3	> 100
*AC104534.3*	19	39 310 806	> 100	14.0	> 100

The 15 most significant distance factor tortuosity genes, for each genome-wide association study (combining all vessels, considering only arteries, and only veins). *P*-values were computed by PascalX^64^ (precision cutoff: 1 × 10^−100^). For full results, refer to Supplemental Dataset 6A–C.

**Figure 4:**
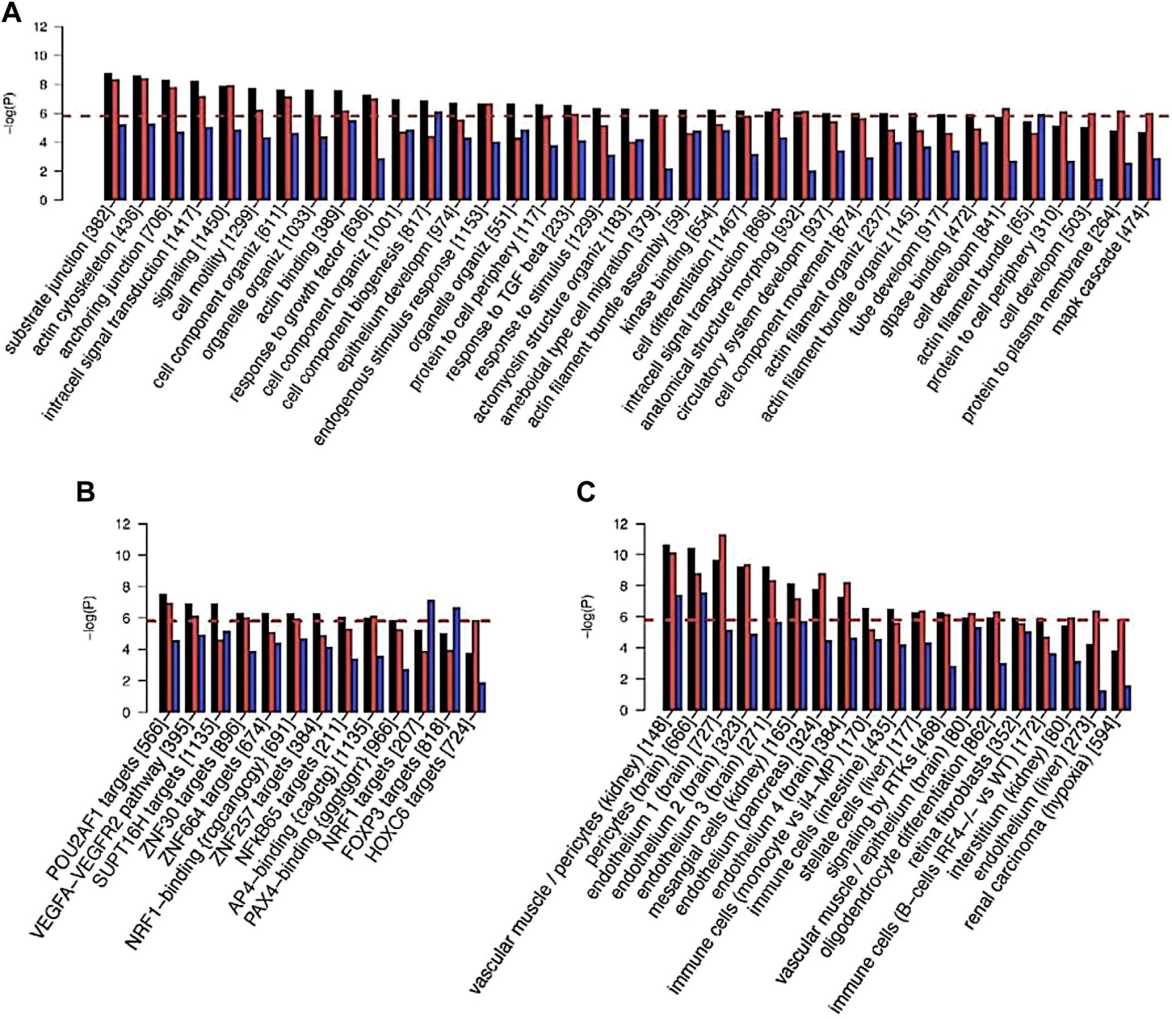
Enriched pathways and gene-sets. Arteries in red, veins in blue, combined-vessel signal in black: scores for 31 120 gene-sets in MSigDB (v7.2)^82^ were calculated by *PascalX*.^64^ Only gene-sets for which significance was reached by ≥ 1 genome-wide association study are shown. The red dashed line indicates Bonferroni-threshold (−log_10_*P* = 5.7). The number of genes in each set is indicated in squared brackets. Gene-set names have been shortened and some redundant gene ontology (GO) categories are not shown. For details, refer to the extended plot in Supplemental Text 13. **A,** Enrichment in GO categories. **B,** Enrichment in pathways referring to a particular molecule (typically a transcription factor) or binding motif. **C,** Enrichment in gene-set obtained from transcriptomic analysis of tissues of treated cell types. TGF = transforming growth factor.

Gene set enrichment (Methods) yielded 78 significant sets in total (Fig 4), with the strongest signals arising from the combined and artery-specific analysis (Supplemental Text 9 and Supplemental Dataset 7A–C). Similarly to genes, many of the pathways pointed to specific biological processes, cellular components, and molecular functions related to vessel integrity and remodeling. These included “human retinal fibroblasts,” “vascular SMCs” (both in the kidney and the neuroepithelium), and “epithelium development.” We also observed a pathway related to “VEGFs,” VEGFA-VEGFR2, which is a well-known therapeutic target for ocular diseases. We highlight several transcription factors and binding motifs for further experimentation (Fig 4B). The role of integrity and development of blood vessels for tortuosity was supported by the enrichment of several gene ontology terms such as “circulatory system development,” “anatomical structure morphogenesis,” and “tube development.” The enriched terms “cell-substrate junction,” “anchoring junction,” “actin,” and “actomyosin” revealed some of the molecular players involved (see Discussion for more details).

Compared to the DF analysis, the alternative tortuosity measures had lower heritability and fewer enriched genes and pathways. However, some were unique and disease-relevant, such as a pathway related to “abnormal cardiac ventricle morphology” (Supplemental Text 2).

### Tortuosity Genes Are Overexpressed in Arteries and Heart Tissues

**Figure 5:**
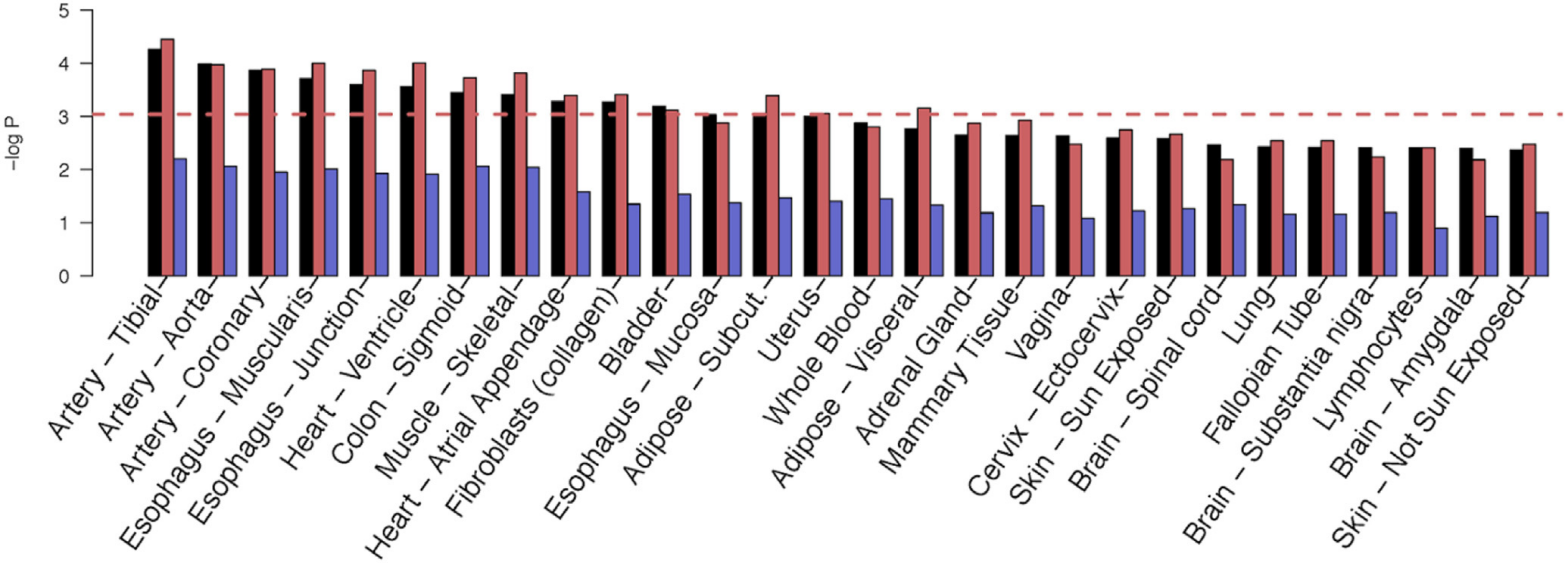
Tissue expression results. Arteries in red, veins in blue, combined-vessel signal in black: tissue-specific gene expression analysis of GTEx (version 8)^69^ performed using *PascalX*.^64^ We defined sets based on the significant genes from each of the 3 genome-wide association studies we carried out and asked whether they were over-expressed in a particular tissue. Only top tissues are shown here, for full results refer to Fig S19.

Performing enrichment analyses across expression data from 54 tissues, we found that tortuosity genes were overexpressed in 3 types of arteries (i.e., aorta, tibial artery and coronary artery), 2 heart tissues (i.e., ventricle and atrial appendage), and, less significantly, fibroblasts and muscular tissues. The profile of enrichment significance values across tissues for tortuosity genes detected by combined-vessel type GWAS analysis is more similar to that of the artery-specific GWAS than that of vein-specific one (Fig 5), which did not result in any significant tissue associations (for a strict Bonferroni threshold of *P* = 0.05/54 = 9.2 × 10^−4^).

### Tortuosity Loci Are Known Disease Variants

Nine of the discovered tortuosity loci had been previously reported as disease variants that mapped to specific genes (Table 4): 3 loci were linked to vascular diseases (coronary heart disease, myocardial infarction, and arterial hypertension), 2 loci were linked to ocular diseases (glaucoma and myopia), 3 loci were linked to other systemic diseases (chronic lymphocytic leukemia, type 2 diabetes, and Alzheimer’s disease), and 1 loci was linked to digestive conditions (diverticular disease). Similarly, we identified 12 loci influencing both tortuosity and disease risk factors. We also uncovered 26 additional disease variants that have not been confidently mapped to a specific gene (Supplemental Text 10).

**Table 4:** Pleiotropic Disease-Variants

Shared SNP	Disease GWAS	Gene	−log_10_*P*	Ref
rs875107	diverticular disease	*ANO1*	22.5	83
rs7588567	glaucoma	*NCKAP5*	21.2	84
rs7119	type 2 diabetes	*HMG20A*	17.5	85
rs936226	hypertension	*CYP1A2*	14.7	86
rs757978	chronic lymphocytic leukemia (CLL)	*FARP2*	10.4	87
rs2753462	myopia	*FARP2*	10.4	88
rs6725887 (∗)	coronary heart disease	*WDR12*	9.6	89
rs6725887 (∗)	myocardial infarction	*WDR12*	9.6	90
rs9381040	Alzheimer's disease	*TREML2*	6.0	91
rs11083475	heart rate (rhythm disorders)	*ACTN4*	> 100	92
rs9555695	waist-hip ratio (obesity)	*COL4A2*	> 100	93
rs2571445	lung function (pulmonary disease)	*TNS1*	> 100	94
rs3791979	intraocular pressure (open angle glaucoma)	*TNS1*	> 100	95
rs17263971	eGFR (Chronic Kidney Disease) and retinal dysfunction	*SYNPO2*	28.7	79,96
rs35252676	pulse pressure (CVD)	*LHFPL2*	26.6	97
rs1378942 (∗)	diastolic blood pressure (CVD)	*CSK*	23.4	98
rs1378942 (∗)	mean arterial pressure (CVD)	*CSK*	23.4	99
rs17355629	pulse pressure (CVD)	*LRCH1*	19.6	100
rs7655064	waist-hip ratio (obesity)	*MYOZ2*	14.5	93
rs6495122	diastolic blood pressure (CVD)	*CPLX3*	14.5	101
rs12913832	intraocular pressure (open angle glaucoma)	*HERC2*	12.3	102
rs9303401	cognitive ability (mental disorders)	*PPM1E*	10.01	103

CVD = cardiovascular diseases; eGFR= estimated glomerular filtration rate; GWAS = genome-wide association study; SNP = single nucleotide polymorphism.

List of variants identified in the tortuosity GWAS (combined-vessel analysis) which were found to be associated with a disease outcome or risk factor in an independent study. We report only exact variants (same rsID in both tortuosity and disease GWAS), which we could confidently map to a gene. Gene *P*-values were computed by *PascalX*.^64^ Variants associated with > 1 disease are marked by a star (∗).

### Genetic Overlap With Cardiometabolic Risk Factors

**Figure 6:**
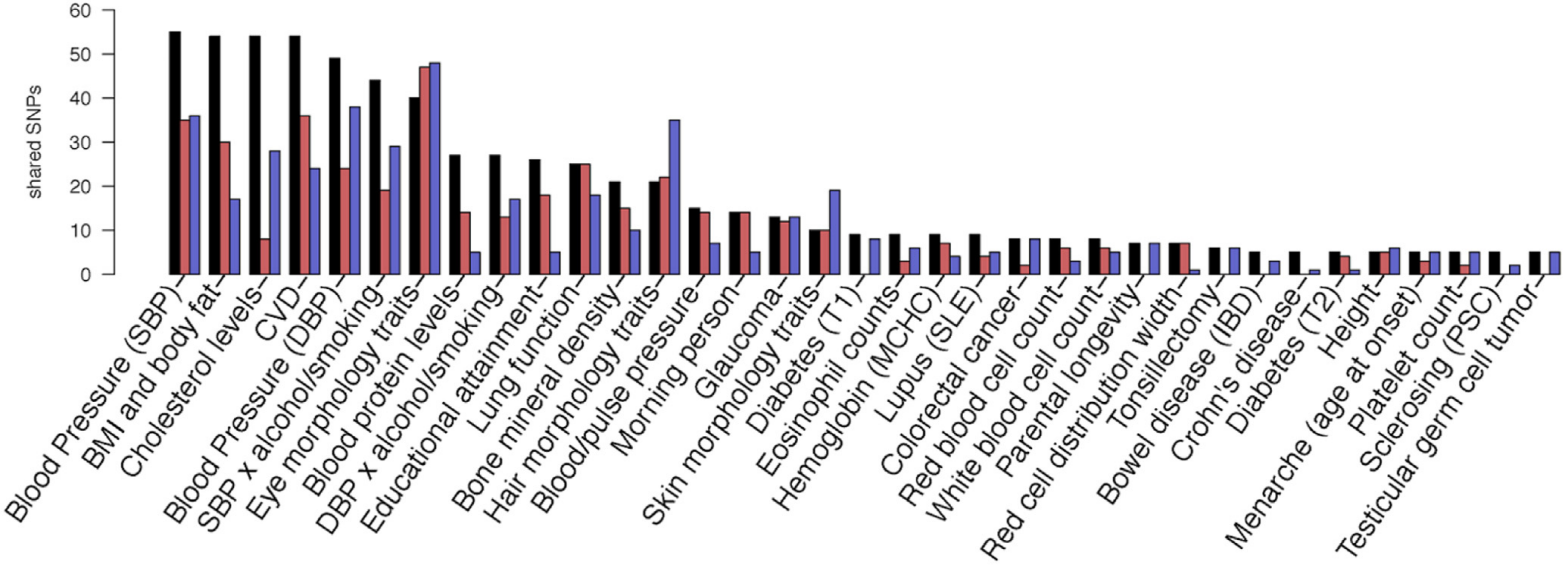
Overlap in genetic signals with diseases and other complex traits. Arteries in red, veins in blue, combined-vessel signal in black: number of variants shared with other traits reported in the genome-wide association study Catalog^70^ (also considering single nucleotide polymorphisms [SNPs] in high linkage disequilibrium with the lead SNP, *r*^*2*^ > 0.8). Only traits with ≥ 5 shared associations are included (for a full list, including rsIDs, refer to the Supplemental Dataset 3). The traits with the highest number of shared SNPs belong to metabolic syndrome (blood pressure, body mass index [BMI], blood cholesterol levels) and cardiovascular disease (CVD). This analysis was generated using functional mapping and annotation of genetic associations (FUMA).^104^ DBP = diastolic blood pressure; IBD = inflammatory bowel disease; MCHC = mean corpuscular hemoglobin concentration; PSC = primary sclerosing cholangitis; SBP = systolic blood pressure; SLE = systemic lupus erythematosus; SNP = single nucleotide polymorphism; T1 = type 1; T2 = type 2.

We expanded our analysis of disease variants to SNPs belonging to the same LD block (Fig 6). We observe a sizable number of tortuosity-associated variants that overlap with CVD (54 SNPs). Several traits related to metabolic syndrome also stand out: blood pressure (55 SNPs for systolic blood pressure, 49 for diastolic blood pressure, and 15 for pulse pressure), blood cholesterol levels (54 SNPs), BMI (54 SNPs), blood pressure linked to alcohol intake and smoking (44 SNPs for systolic blood pressure + alcohol, 27 for diastolic blood pressure + alcohol), and type 2 diabetes (5 SNPs). In addition, other CVD risk factors share a high number of variants associated with tortuosity, such as protein levels (27 SNPs) and type 1 diabetes (9 SNPs). Finally, we detected an overlap with various eye morphology traits, including optic disc morphometry (40 SNPs).

### Causal Effects Between Tortuosity, BMI, and LDL

Using inverse-variance weighting Mendelian Randomisation (MR), we observed that exposure to elevated (standardized) levels of LDL reduced the tortuosity of veins by 3% (*P =* 0.02) and arteries by 5% (*P =* 0.001). Conversely, increased venous (but not arterial nor combined) tortuosity reduced BMI by 4.4% (*P =* 0.01) (Supplemental Text 11).

## Discussion

Blood vessel tortuosity is a complex trait whose variation is induced in part during developmental angiogenesis and vascular differentiation and in part through vessel remodeling due to pathological processes in adult life. Both sources of variation are modulated by the environment, but also genetically through gene and regulatory variants that subtly modulate these processes. In order to better understand the involved genetic architecture, we conducted the largest GWAS on retinal vessel tortuosity to date, identifying 173 novel loci and pinpointing numerous genes and gene-sets enriched with these primary association signals. Leveraging the unprecedented number of hits, we performed MR that revealed the causal relationships between retinal tortuosity, BMI, and blood lipids. This provides context for the considerable overlap we observed between variants associated with vessel tortuosity and cardiometabolic diseases as well as their risk factors. Our results were consistent with the overexpression of tortuosity-related genes in the aorta, tibial artery, coronary artery, and heart tissues. We found these genes to be involved in the development of blood vessels, the maintenance of vessel integrity, and the remodeling as a consequence of disease processes.

### Vessel Integrity

Several enriched gene ontology categories that are integral to vessel development were enriched, namely “morphogenesis of anatomical structures,” “development of circulatory system,” and “tube development.” Similarly gene ontology categories pertinent to the structural integrity of vessels and the stability of specific tissues were highlighted: “cell-substrate junction” and “anchoring junction” which are responsible for the mechanical attachment of a cell and its cytoskeleton to the extracellular matrix. Molecularly, “actin cytoskeleton,” “actin binding,” “actin filament bundle organization,” and “positive regulation of actin filament bundle assembly” highlighted the important role of actin.

Among the top hits, we found genes directly related to vessel integrity. The product of *ACTN4* contributes to cell adhesion and to assembly of the tight junction by mediating actin filament bundling. The paralogues *COL4A1* and *COL4A2* provide structural support and elasticity to connective tissues by forming the heterotrimer α1α1α2, which is the most abundant collagen in the basement membrane.^105^ We found both *COL4A2* and *ACTN4* to be overexpressed in vascular tissues (Supplemental Text 12). Two more genes with actin-related activity were also among our top hits: *TNS1*, which promotes cell migration and regulates angiogenesis,^106^ and *SYNPO2*, which is activated by actin polymerization, highly expressed in SMCs^79^ and known to provide structural integrity to blood vessel walls.^107^ Finally, we identified 3 genes coding for galectins, which are involved in adhesion to the connective tissue via modulation of cell-cell and cell-matrix interactions^77^: *LGALS7*, its paralog *LGALS7B* and *LGALS4*.

### Vessel Remodeling

Pathological stresses such as inflammation, infection, or injury can cause remodeling of vessels, manifesting as occlusions, kinks, tubulations, or other collateral formation of vessels. Pathway analysis identified gene sets of ECs (4 sets), SMCs (2 sets), fibroblasts (1 set), and pericytes (1 set) which are the basic cell types composing vessel walls. Dysregulated response of vascular SMC can induce hypertension, and excessive proliferation of these cells contributes to CVD progression.^108^ Endothelial cells dysfunction can lead to hyperpermeability, neurovascular decoupling, and proinflammatory responses.^7^ We identified a gene set for “human retinal fibroblasts'' consistent with the fact that this cell type is the most common in connective tissue and involved in maintaining the extracellular matrix. Under stress, fibroblasts proliferate, resulting in the accumulation of extracellular materials that ultimately limits elasticity.^109^ In addition, we found enrichment in a gene set related to “mesangial cells,” which are kidney-specific pericyte cells. Retinal capillaries are composed of ECs and pericytes. These contractile cells control blood flow in capillaries^110^ and their function is inhibited under stress, such as in high glucose conditions typical in diabetes.^111^ Therefore, dysregulation of these gene sets has the potential to induce vessel remodeling under stress.

We identified genes directly involved in vessel remodeling. In particular, *FLT1* plays a role in the process of collateral vessel formation, which is a form of vascular remodeling in response to stress, such as hypoxia or hypertension.^112^ *FLT1* is transcribed in several tissues, including arteries and heart,^69^ and translated into VEGFR1. VEGFR1 is upregulated in response to microinflammation in the early stages of several vascular diseases.^112^ In the retina, VEGFR1 is observed in ECs, SMCs, pericytes, and RPE cells (which modulate fibroblast proliferation), and excess VEGFR1 contributes to vessel leakage and angiogenesis.^112^

### Associations With Diseases

We detected pleiotropic effects of tortuosity loci, which we showed to be independently associated with CAD, myocardial infarction, hypertension, diabetes, chronic lymphocytic leukemia, Alzheimer’s disease, myopia, and glaucoma. We also found tortuosity related genes to be involved in disease pathomechanisms. *ACTN4*, our top hit, was recently associated with vasorelaxation,^113^ a mechanism that can lead to hypertension when malfunctioning. The lead SNP in *ACTN4* tortuosity (rs1808382) is also independently associated with CAD.^44^ *COL4A1* mutation has been reported as the cause of familial retinal arteriolar tortuosity^78^ and cerebral small vessel disease^114^ vessel leakage and hyperpermeability.^115^ Fittingly, *COL4A2* also figured among our variants with pleiotropic effects on disease risk (Table 4). Variants in the fetal genome near *FLT1* have been associated with preeclampsia,^116^ a condition of pregnant women presenting with hypertension and damage to the liver and kidneys, whose underlying mechanism involves abnormal formation of blood vessels in the placenta.^117^ Retinal vessel modifications have been observed to precede clinical onset of preeclampsia and persist up to 12 months postpartum.^118^, ^119^, ^120^

We elucidated causal links between tortuosity and disease risk factors by applying MR. Specifically, we established that elevated LDL exposure causally reduces arterial tortuosity. High-LDL is known to cause the buildup of atherosclerotic plaque,^121^ which has been clinically linked to arterial tortuosity.^122^^,^^123^ In fact, arteriosclerosis may make retinal arterial walls less flexible and thereby reduce their DF. We observed a *negative* causal effect of venous tortuosity on BMI, despite the known *positive* correlation between BMI and retinal tortuosity,^124^ suggesting that environmental factors may play a role in the relationship between BMI and vascular tortuosity.

### Limitations

This study was subject to the following limitations: First, we focused on the DF as a tortuosity measure, since the corresponding GWAS revealed many more significant loci, genes, and pathways, as well as a higher heritability estimate in comparison to the alternative curvature-based tortuosity measures. These measures are more sensitive to local physiological vessel features, such as aneurysms or sharp bending (“kinks”), while DF only captures the total vessel elongation. Yet, they may also be more sensitive to the vessel segmentation procedure than the DF. Interestingly, the GWAS for these measures revealed several specific genes and pathways that were not significant in the DF analysis, which may be associated with pathologies manifesting as local disruptions in the microvascular network. Further work is needed to elucidate to what extent the stronger association signals for the DF are due to its robustness as a tortuosity measure or its quality to capture *total* vessel elongation as the most physiologically relevant trait. Second, due to the small size of our replication metacohort, we essentially just had sufficient power to verify an *overall* concordance with the discovery cohort in terms of the highly significant correlation between SNP- and gene-effect sizes, providing independent evidence that they were not driven by any artifacts specific to the UKBB.^51^ Even though we could only replicate very few of our SNP-wise hits (4/136 at FDR = 0.1), the situation was somewhat better at the level of genes (57/262 at FDR = 0.5), underlining the usefulness of signal aggregation from SNPs to genes.^64^^,^^65^ Our specific findings should thus be viewed as discoveries in the United Kingdom population that still need to be replicated in a much bigger cohort than our Swiss metacohort. Finally, we did not attempt to stratify this population by existing diseases, including retinal disorders or other ocular conditions, nor remove subjects with a retinal image from 1 eye only, all of which may affect our results.

This study exploits advanced automated image processing to characterize different *vessel type specific* retinal tortuosity measures from retinal fundus images of close to 70 000 subjects to conduct a high-powered GWAS on this trait. The resulting significant association signals allowed us to provide novel insights into the genetic architecture of retinal tortuosity. Specifically, we identified a large number of genes, annotated gene-sets and tissues relevant for this trait, and revealed pleiotropic links with and causal effects to or from disease-related traits. Our study makes important methodological advancements in the large-scale analysis of medically relevant images, which can be applied to other retinal and nonretinal features both in fundamental and clinical research. Our findings provide a significant progress in understanding of molecular players and mechanisms modulating retinal vessel tortuosity and their links to ocular and cardiometabolic diseases, which is fundamental for developing better tools for their diagnosis and treatment.
