# Supplementary material for: Genome-wide Association Studies of Retinal Vessel Tortuosity Identify Numerous Novel Loci Revealing Genes and Pathways Associated With Ocular and Cardiometabolic Diseases
Source: Ophthalmol Sci. 2023 Feb 16;3(3):100288. doi: 10.1016/j.xops.2023.100288 (PMC10149284; doi:10.1016/j.xops.2023.100288)
Supplement: Supplementary data [file mmc1.docx]

**SUPPLEMENTAL MATERIAL**

**Genome-Wide Association Studies of Retinal Vessel Tortuosity Identify Numerous Novel Loci Revealing Genes and Pathways Associated with Ocular and Cardiometabolic Diseases**

Mattia Tomasoni, PhD^1,2,7,†^; Michael Johannes Beyeler, MSc^1,2,†^; Sofia Ortin Vela, MSc^1,2,†^; Ninon Mounier, PhD^2,3^; Eleonora Porcu, PhD^2,3,4^; Tanguy Corre^1,2,3^;

Daniel Krefl, PhD^1,2^; Alexander Luke Button, PhD^1,2^; Hana Abouzeid, MD^5,6^; Konstantinidis Lazaros, MD^7^; Murielle Bochud, MD, PhD^3^; Reinier Schlingemann, MD, PhD^7^; Ciara Bergin, PhD^7^; Sven Bergmann, PhD^1,2,8^

^1^Dept. of Computational Biology, University of Lausanne, Lausanne, Switzerland;

^2^Swiss Institute of Bioinformatics, Lausanne, Switzerland; ^3^Center for Primary Care and Public Health (Unisanté), University of Lausanne, Lausanne, Switzerland; ^4^Center for Integrative Genomics, University of Lausanne, Lausanne, Switzerland; ^5^Division of Ophthalmology, Geneva University Hospitals, Switzerland; ^6^Clinical Eye Research Center Memorial Adolphe de Rothschild, Geneva, Switzerland; ^7^Jules-Gonin Eye Hospital, Lausanne, Switzerland; ^8^Dept. of Integrative Biomedical Sciences, University of Cape Town, Cape Town, South Africa; ^†^Authors contributed equally to this work.

**Address for Correspondence:**

Sven Bergmann, PhD

University of Lausanne

Genopode

1016 Lausanne

Switzerland

E-mail: [sven.bergmann@unil.ch](mailto:sven.bergmann@unil.ch)

**TABLE OF CONTENTS**

[**SUPPLEMENTAL DATASETS**](#_ojh4mqc9s2lh) **3**

[**SUPPLEMENTAL METHODS**](#_ff9x6ijac097) **3**

[**Text 1: Quality Control in tortuosity measurements**](#_2vinuneohoxi) **3**

[**Text 2: DF and other tortuosity measures**](#_rt5h2eedofeb) **3**

[Definition of DF](#_qypjq2kxk27a) 3

[Definitions of six, alternative, curvature-based measures](#_9mt4e5ymu1gd) 4

[PCA of DF and curvature-based measurements](#_jehvo9noyzod) 4

[Heritability of DF and curvature-based measurements](#_rfmiyyquptic) 5

[Enrichment analysis of curvature-based measurement associations](#_qdgab711bti4) 5

[**Text 3: Distribution of DF**](#_bukiox78mblp) **7**

[Correlation between measurements of the left and right eye](#_ia5qw2tujcpb) 7

[Systematic left-right differences](#_8ouul771ptxm) 7

[DF tortuosity across cohorts](#_1l8uw4auxawn) 8

[Stratified DF analysis: sex, age and vessel type](#_pewgk3rv2p3p) 8

[**Text 4: Image segmentation, and deep learning classification of arteries and veins**](#_t760qyjqsc0j) **9**

[Image processing methods](#_pujogi6zc48y) 9

[Accuracy of vessel type classification](#_gtpox3bavk6q) 9

[Censoring unclearly classified vessels](#_bfjga55rijm4) 10

[GWAS with random vessel type calling](#_dx8a81m4vhi0) 11

[**Text 5: Replication Analysis**](#_etat7oocs34j) **11**

[Power calculations](#_ackghcgbu8iu) 11

[Correlation of effect sizes in the meta-cohort](#_jzduawlqpnx9) 12

[Replication of hits in the meta-cohort](#_o1demd7e3w1y) 14

[**SUPPLEMENTAL RESULTS**](#_5evio3kd1ule) **15**

[**Text 6: Baseline Characteristics**](#_ghpq6xwuq0uj) **15**

[**Text 7: Correlation with disease status**](#_6r0px2ef4fnw) **15**

[**Text 8: Replication and confounders**](#_po5cjueox4ke) **16**

[Replication of known hits](#_g0n3nhmh6j0z) 16

[Variance explained by major confounders vs. genetics](#_8t4c5ictu58k) 18

[**Text 9: Vessel-type comparisons for SNPs, Genes and Pathways**](#_pg9tblmqve1u) **19**

[**Text 10: Genetic associations with disease and risk**](#_rcetvdj3f319) **20**

[Tortuosity variants associated with disease outcome](#_os60zn4eahat) 20

[Tortuosity variants associated with disease risk factors](#_wkw8vql0qzfz) 20

[**Text 11: Mendelian Randomization**](#_ebu33frzztqv) **21**

[**Text 12: ACTN4 and COL4A2 over-expression**](#_jfg00pwwqe01) **22**

[**Text 13: Full gene set enrichment results**](#_nwnj3wie6npf) **23**

[Text 14: Dependency of GWAS on covariates selection](#_dh03y7spjei2) 28

# SUPPLEMENTAL DATASETS

[Supplemental Dataset 1 - 175 lead SNPs](https://docs.google.com/spreadsheets/d/1mhy8mRZs_bUdFyL331dPPU_N0-mBPAQ9LsHMCzPFGfE/edit?usp=sharing)

[Supplemental Dataset 2 - overlap with GWAS Catalog (SNPs in LD)](https://docs.google.com/spreadsheets/d/1wYTp7pweURdA5-MINn5LWXQ7oT_4Z1e8E56kAgDkfbc/edit?usp=sharing)

[Supplemental Dataset 3 - overlap with GWAS Catalog (SNPs with matching rsIDs)](https://docs.google.com/spreadsheets/d/1dTh9yhHiRfQqBzPkUoHazvo4vSvBEJX9gL_bFGzfoR0/edit?usp=sharing)

[Supplemental Dataset 4A - significant SNPs](https://docs.google.com/spreadsheets/d/1PUSbmK_snLIdW4wtx7YqEUhrCPlWRH6fLfsUE4T-3dg/edit?usp=sharing) (combined)

[Supplemental Dataset 4B - significant SNPs (artery)](https://docs.google.com/spreadsheets/d/1ei2pogiEswGmz36-dXvMa61Kd4vFvVDVkCcIuqG43po/edit?usp=sharing)

[Supplemental Dataset 4C - significant SNPs (vein)](https://docs.google.com/spreadsheets/d/1Q0tyyNL7lOfLIqX4nVHbIJdJpGFrKCV3M8MakI2zaa8/edit?usp=sharing)

[Supplemental Dataset 5 - replication of lead SNPs](https://docs.google.com/spreadsheets/d/1On_QBISreAj58mBZkIl_r_o3HhlRMTeg9cv4FFsypEg/edit?usp=sharing) and genes

[Supplemental Dataset 6A - gene scores (combined)](https://docs.google.com/spreadsheets/d/19_1skGdPyYilA_bOGNaWrdezrCRauxTGGuFQyWoABKc/edit?usp=sharing)

[Supplemental Dataset 6B - gene scores (artery)](https://docs.google.com/spreadsheets/d/1koU44Ca6jRZPADqk8cWGkgl0NngQGG5MzmzQBQX673o/edit?usp=sharing)

[Supplemental Dataset 6C - gene scores (vein)](https://docs.google.com/spreadsheets/d/1T4jqBUnd5dxqsyrVC3jzFhWlRVPqTP9jGJtqLAIPETc/edit?usp=sharing)

[Supplemental Dataset 7A - gene set enrichment (combined)](https://docs.google.com/spreadsheets/d/14iuAwCYAY49CwaqZqigXPBiP2iBgoZnYO7zbyMktnpw/edit?usp=sharing)

[Supplemental Dataset 7B - gene set enrichment (artery)](https://docs.google.com/spreadsheets/d/1vLyStn6tPZ4Prbg93IQMJDM3vl-qnGE29a_teODlegU/edit?usp=sharing)

[Supplemental Dataset 7C - gene set enrichment (vein)](https://docs.google.com/spreadsheets/d/1MakdTwy7JKr3L3E_WyDCePeZj4fJbRzRTt9Ib7NjrtQ/edit?usp=sharing)

# SUPPLEMENTAL METHODS

## Text 1: Quality Control in tortuosity measurements

The quality control (QC) procedure is performed during data extraction. The objective is to remove (i) lower quality images and (ii) images containing artifacts. QC relies on thresholding being applied to two distributions: 1) Distribution of the total number of equally spaced diameters that were fitted to the vessels of each eye. Images between with values 11 000 and 20 000 passed QC. 2) Distribution of the number of vessels contained in each eye. Images with values between 100 and 250 passed QC. By visual inspection, we fine tuned these thresholds to discriminate (i) low-quality images that were too dark, too light, out of focus (lower-bound thresholds), or (ii) images that contained spurious vessels, i.e., artifacts of the picture that were being erroneously segmented as vessels (higher-bound thresholds). Images that pass QC (roughly two out of three) are further processed to extract several tortuosity measures.

## Text 2: DF and other tortuosity measures

### Definition of DF

We consider a vessel as a curve in a two dimensional space on the interval [t_0_, t_1_]. For a given curve there are different tortuosity measures: The simplest one, the "Distance Factor" (DF), is calculated as the total arc length over total chord length. This measure computes the tortuosity of the segment by examining how long the curve is relative to its chord length.

$$DF = \frac{s(C)}{chord(C)}$$

Let $s(C)$ be the arc length of the curve C, and $chord(C)$ its chord length:

$s(C) =\int_{{t_{0}}}^{t_{1}} \sqrt{{x'(t)}^{2} +{y'(t)}^{2}} dt ,$ $chord(C) = \sqrt{{(x(t_{1})-x(t_{0}))}^{2} +{(y(t_{1})-y(t_{0}))}^{2}}$

where the prime denotes derivation with respect to *t*.

### Definitions of six, alternative, curvature-based measures

A first curvature-based tortuosity measure is obtained as the integral over the absolute value of curvature along the entire curvature:

${\tau_{2} =\int_{{t_{0}}}^{t_{1}} \left| \kappa(t) \right|dt}$, where the curvature is defined as: $|\kappa(t)|=\frac{|x'(t)y''(t) - x''(t)y'(t)|}{\left[ {y'(t)}^{2} + {x'(t)}^{2} \right]^{3/2}}$

This is equivalent to the inverse curvature radius *R(t)*, i.e. the radius of a circle that is tangent to the curve at t: |$\kappa$| = *1/R*.

A second tortuosity measure is the integral over the curvature squared along the entire curve:

$${\tau_{3} =\int_{{t_{0}}}^{t_{1}} {\kappa(t)}^{2} dt}$$

Finally, four tortuosity measures arise from normalizing and 𝛕_3_ by division through either the curve or the arc length:

$${\tau_{4} =\int_{{t_{0}}}^{t_{1}} \frac{\left| \kappa(t) \right|}{s(C)} dt ,}{\tau_{5} =\int_{{t_{0}}}^{t_{1}} \frac{{\kappa(t)}^{2}}{s(C)} dt ,}{\tau_{6} =\int_{{t_{0}}}^{t_{1}} \frac{\left| \kappa(t) \right|}{chord(C)} dt},{\tau_{7} =\int_{{t_{0}}}^{t_{1}} \frac{{\kappa(t)}^{2}}{chord(C)} dt}$$

We note that, by definition, 𝛕_2_ and 𝛕_3_ depend on the length of the curve (with non-zero curvature). In the case of vessels with constant curvature radius: 𝛕_2_= *s(C)/R* and 𝛕*_3_= s(C)/R^2^*. Our analysis shows that 𝛕_2_ differs from other measures the most, followed by 𝛕_3_, while 𝛕_4-7_ are indeed quite similar to each other (see Supplemental [Figure 1](#fig_PCA_alternative))

### PCA of DF and curvature-based measurements

**
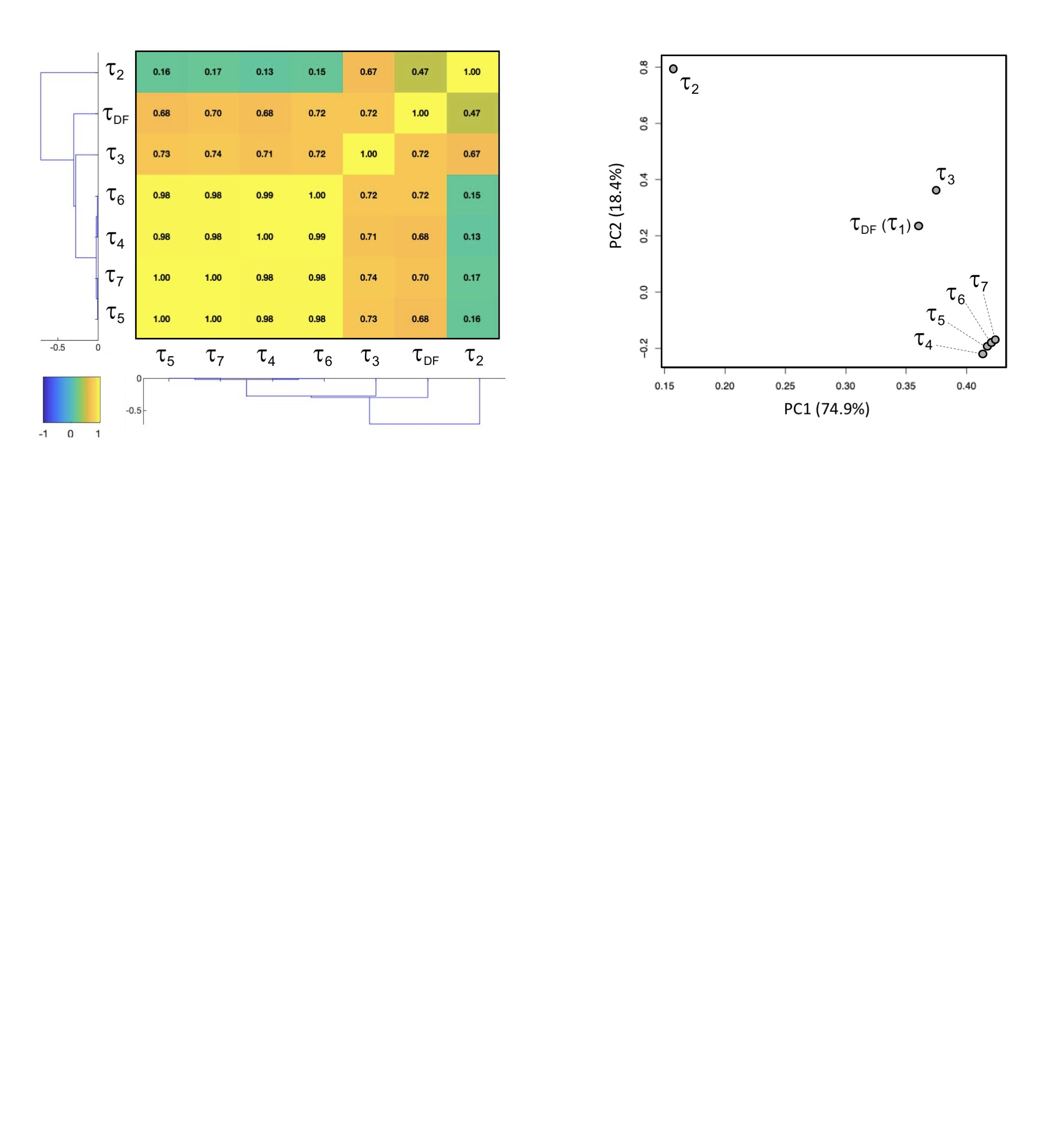

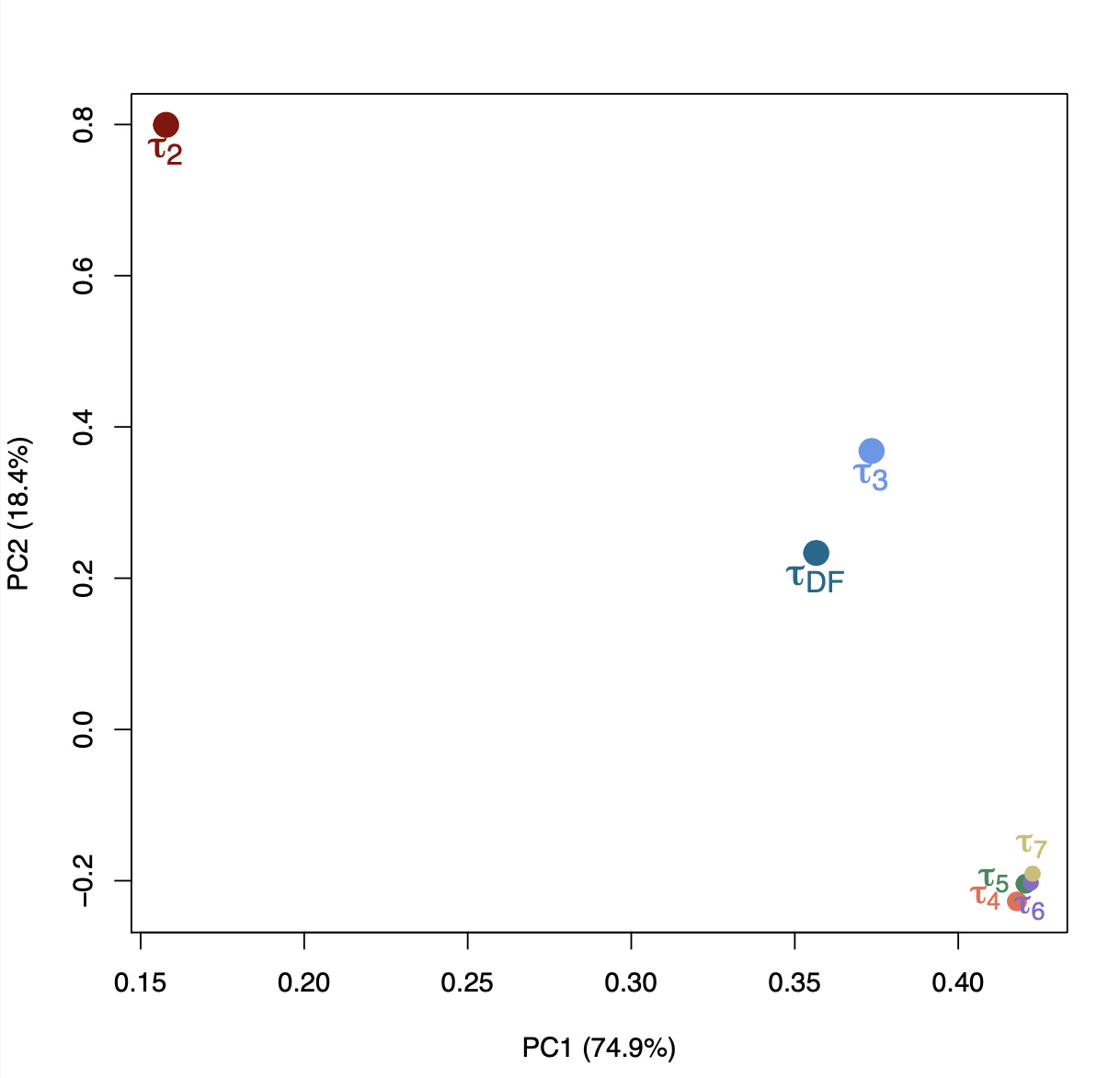
**

**Supplemental** [Figure 1](#figur_PCA_alternative) **| Dimensionality reduction on tortuosity measurements across 62 751 individuals.** Left: Hierarchical clustering of the pairwise correlations-matrix. Correlations are measured using the Pearson correlation coefficient. Right: We performed dimensionality reduction using a Principal Component Analysis (PCA) approach and plotted PC2 against PC1.

### Heritability of DF and curvature-based measurements

| **Measure** | ***h^2^_SNP_*** | ***Lambda GC*** | ***Mean Chi^2^*** | ***Intercept*** | ***Ratio*** |
| --- | --- | --- | --- | --- | --- |
| DF (Distance Factor) | 0.25 (0.025) | 1.14 | 1.31 | 1.01 (0.01) | 0.03 (0.03) |
| 𝛕_2_ | 0.11 (0.011) | 1.08 | 1.11 | 0.98 (0.01) | < 0 |
| 𝛕_3_ | 0.11 (0.012) | 1.09 | 1.13 | 0.99 (0.01) | < 0 |
| 𝛕_4_ | 0.12 (0.011) | 1.11 | 1.15 | 1.00 (0.01) | 0.02 (0.05) |
| 𝛕_5_ | 0.12 (0.011) | 1.10 | 1.14 | 1.00 (0.01) | < 0 |
| 𝛕_6_ | 0.13 (0.012) | 1.11 | 1.16 | 1.00 (0.01) | 0.01 (0.04) |
| 𝛕_7_ | 0.12 (0.011) | 1.10 | 1.15 | 1.00 (0.01) | < 0 |

**Supplemental** [Table 1](#table_LDscores_h2_alternative) **| SNP-based heritability of alternative tortuosity measures. h^2^_SNP_** is the portion of phenotypic variance cumulatively explained by the SNPs. Traits defined by the six alternative tortuosity measures were less heritable than the one defined by the Distance Factor. *Lambda GC* is the measure of inflation (it measures the effect of confounding and polygenicity acting on the trait). *Intercept* is the LD Score regression intercept (values close to 1 indicates little influence of confounders, mostly of population stratification). *Ratio* is the ratio of the proportion of the inflation in the *Mean Chi^2^* that is not due to polygenicity (a ratio close to, or smaller than, 0 is desirable as it indicates low inflation from population stratification). SE are given in parentheses.

### Enrichment analysis of curvature-based measurement associations

The alternative tortuosity measures have less significant SNPs, genes and pathways overall compared to the distance factor, but harbor few unique pathways, among which is a pathway, in 𝛕_6,_ called “abnormal cardiac ventricle morphology” (-log_10_ p=5.8) from the human phenotype oncology (HP) group. Being the gene scores of them highly significant in our analysis.

On the phenotypic level, we found that tau 2 (total curvature) is least similar to all the others, that tau 3 (total squared curvature) is comparably similar to the DF, and that tau 4-7 (average curvature) form a distinct cluster of similar measurements.

Here, despite all alternative measures having significantly lower SNP-wise heritability than the DF, we found eighteen genes ([Figure 2](#fig_alt_specific)a) and four pathways ([Figure 2](#fig_alt_specific)b) specific to them, i.e. not present in the DF. Among them are genes with potentially relevant annotations, as described by GeneCards [[24]](https://paperpile.com/c/4pBVkZ/qOyc): OCA2 (Oculocutaneous Albinism 2), the overall top hit, is a determinant of eye color, and associated with albinism. TRIOBP, the most significant specific hit in tau 2, is a structural protein binding to F-actin, and acts as a stabilizer of the cytoskeleton. LGALS1 is implicated in modulation of cell-cell and cell-matrix interactions, and thus a structural protein that might affect, among other things, vessel bendiness. GLIS3 is a zinc-finger protein that plays a role in eye development.

We also identified a cluster of four genes (LRIT1, LRIT2, CDHR1, RGR) on locus 10q23 (Supplemental [Figure 2](#fig_alt_specific)c) through STRING [[25]](https://paperpile.com/c/4pBVkZ/kJEr), all of which have been associated with eye disorders. RGR has been associated with Retinitis pigmentosa. LRIT1, LRIT2 and CDHR1 have been associated with nanophthalmos, a developmental eye disorder characterized by small eyes. Molecularly, CDHR1 is a calcium-dependent cell adhesion molecule expressed in blood vessels, and thus likely to affect their morphology.

Two out of the four pathways both specific to the average curvature measures, are plausibly related to vascular changes. The first, GO_CELL_JUNCTION_ORGANISATION, is a GO set of genes influencing the tightness of connection between neighboring cells. The second, HP_ABNORMAL_CARDIAC_VENTRICLE_MORPHOLOGY, Human Phenotype Ontology set of genes associated with disease-related abnormalities in cardiovascular tissue. This pathway has three genes strongly driving the signal: 1) RYR1, a sarcoplasmic reticulum calcium release channel, 2) MYOZ2, a sarcomeric protein involved in calcium-dependent signal transduction, and 3) FADD, an apoptotic adapter molecule. Interestingly, no pathways were specific to the most dissimilar measure, tau 2.

In summary, first, this confirms two observations we made on the phenotype level: 1) tau 2 with twelve unique gene hits is the most dissimilar from the others, and 2) tau 4-7 are almost identical and can probably be treated as one measure. Second, we identified relevant genes and pathways specific to these measures, indicating that they may capture disease-relevant vascular changes the DF is not sensitive to.

a.
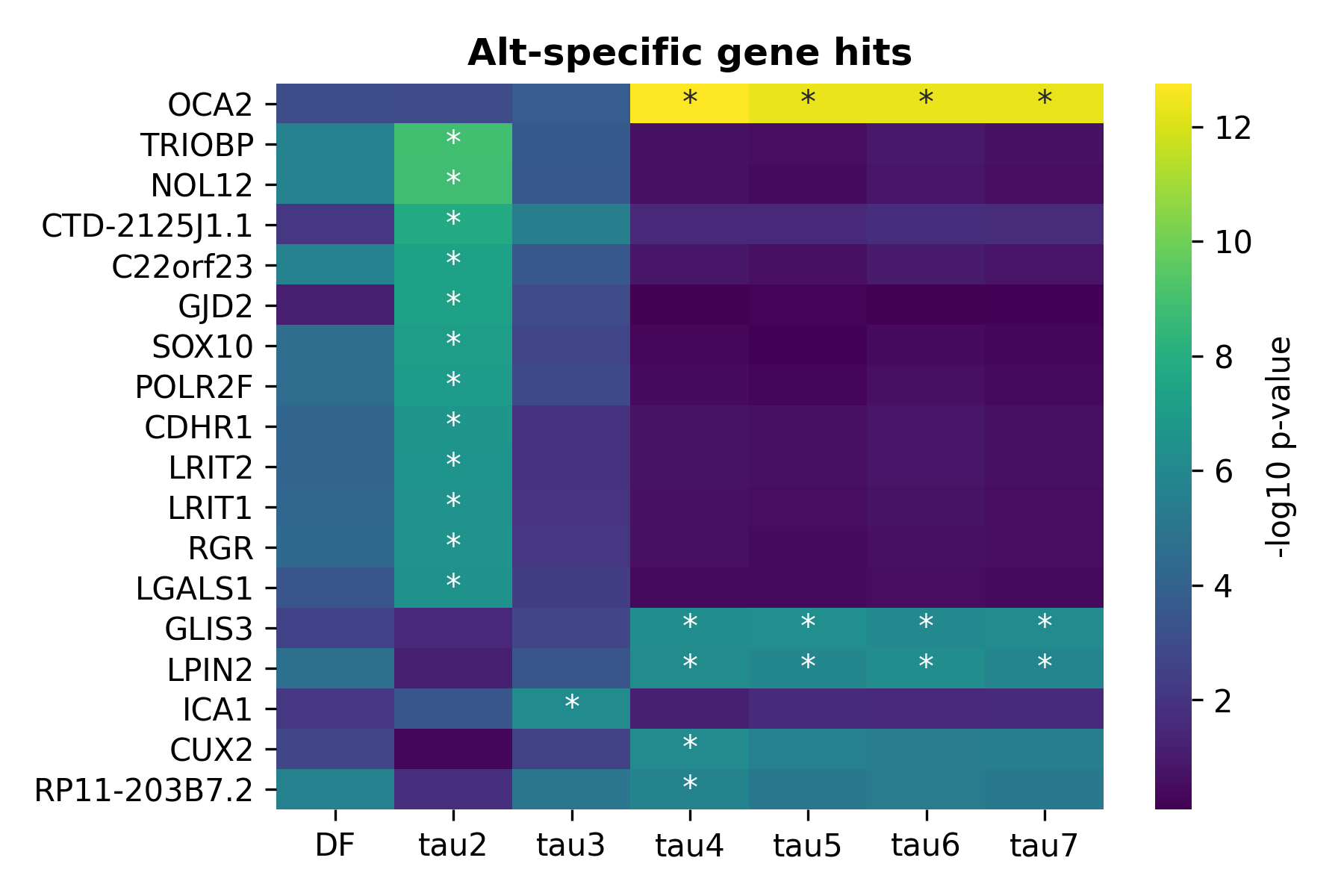


b.


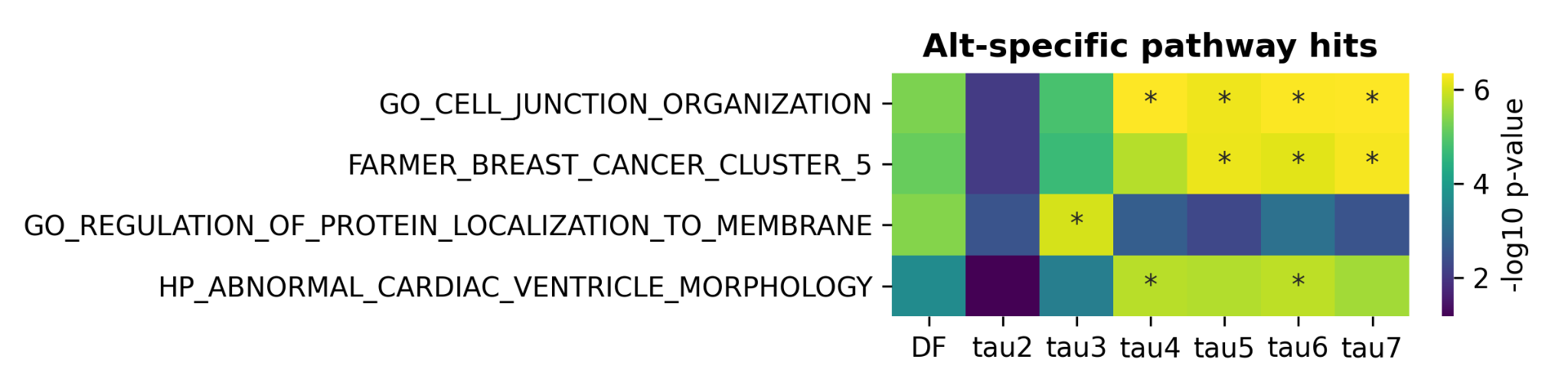


c.


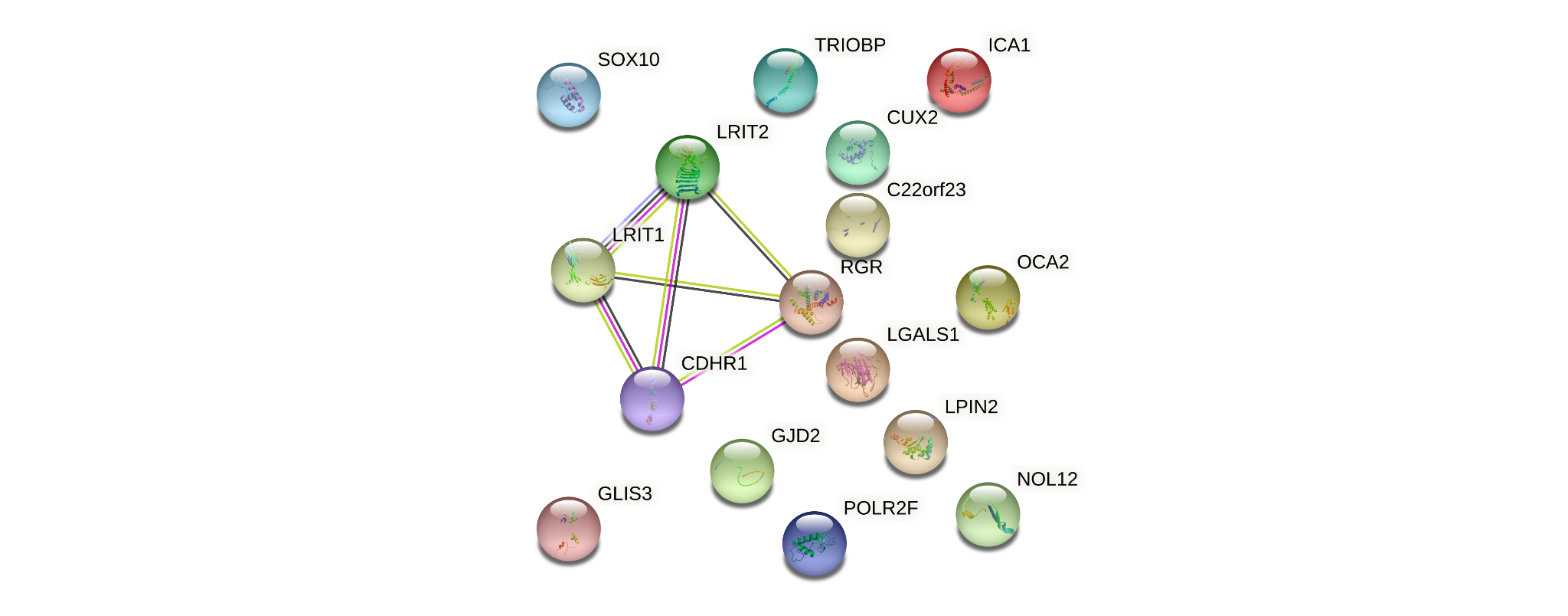


**Supplemental** [Figure 2](#figur_alt_specific) **| Genes and pathways specific to alternative tortuosity measures.** We use a Bonferroni-corrected -log10 p-value significance threshold of 5.71 for genes, and 5.79 for pathways. Significant hits are marked with an asterisk (*). The tables are ordered by maximal significance.

1. All the genes and pathways that are significant in one of the alternative tortuosity measures, but not in the distance factor (DF), are displayed. There are twelve genes specific to tau 2, one to tau 3, and five to the very similar measures tau 4-7.
2. Four pathways are specific to the alternative tortuosity measurements: one in tau 3, three in tau 4-7, but none were found in tau 2. They contain 577, 10, 170, and 394 scored genes respectively.
3. STRING [[25]](https://paperpile.com/c/4pBVkZ/kJEr) cluster of four genes (LRIT1, LRIT2, CDHR1, RGR), all of which reside on locus 10q23, and have been associated with eye pathologies.

## Text 3: Distribution of DF

### Correlation between measurements of the left and right eye

**
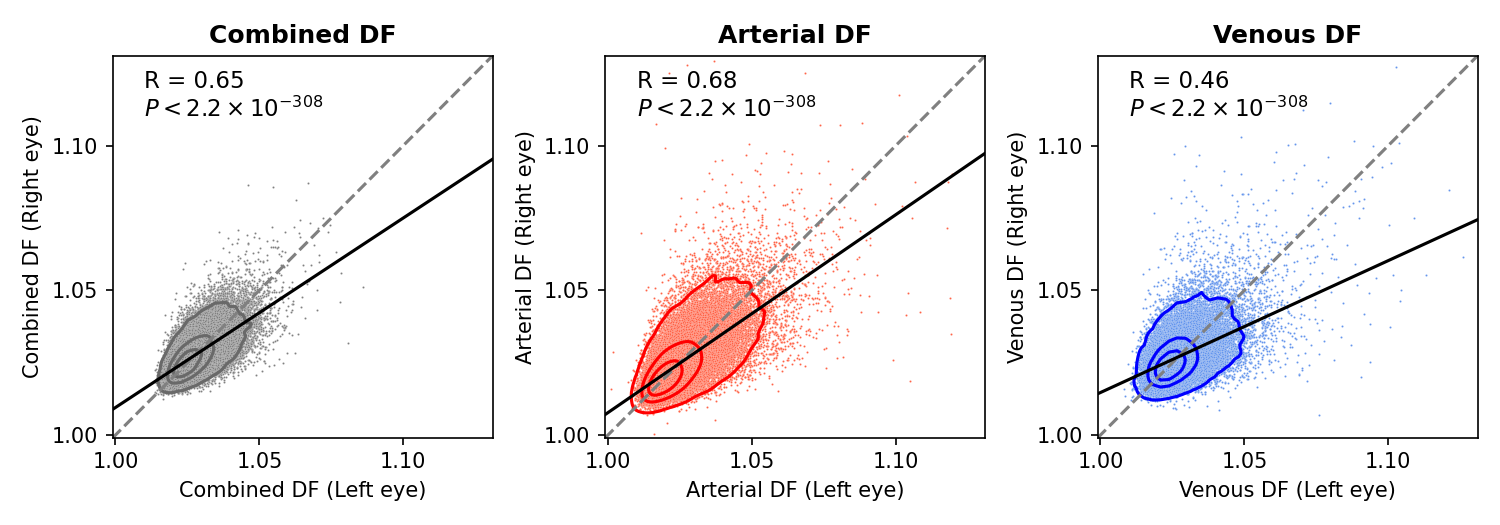
**

**Supplemental** [Figure 3](#figur_DF_eye_age)**:** DF measurements are significantly correlated (Spearman’s R) across eyes. The correlation between eyes is higher in arteries (R=0.68) than in veins (R=0.46). In solid black is the regression line; in dotted gray is x=y. The following participants are not visible within the chosen bounds: Arterial DF: 1914784 (1.14, 1.09), 2065513 (1.14, 1.08), 4872563 (1.15, 1.03), 4609835 (1.03, 1.14); Venous DF: 5093514 (1.41, 1.07), 5374275 (1.15, 1.09), 5664283 (1.57, 1.03), 2536956 (1.13, 1.14), 4660418 (1.02, 1.15), 5615035 (1.05, 1.20).

### Systematic left-right differences


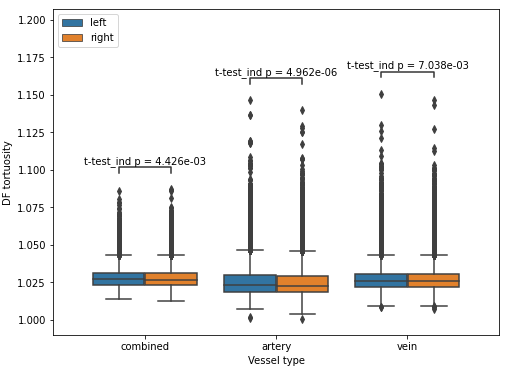


Supplemental [Figure 4](#figur_lr_difference): We observe that left eyes have slightly higher DF across vessel types, but the standardized effect sizes (mean difference divided by standard deviation) are very small: 0.022, 0.034 and 0.022 for combined vessel types, arteries and veins respectively.

### DF tortuosity across cohorts

**
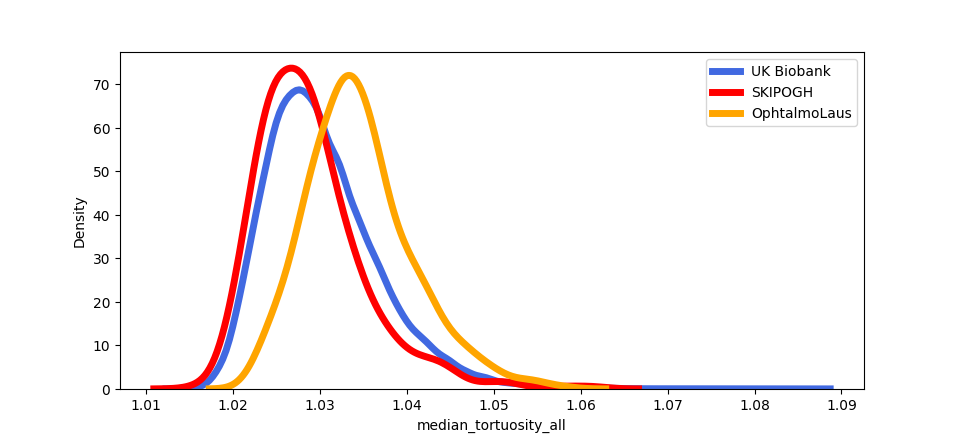
**

**Supplemental** [Figure 5](#figur_DF_hist) **| Distribution of DF tortuosity across cohorts.** UK Biobank mean±SD= 1.030±6.5×10^-3^; SKIPOGH mean±SD=1.029±6.2×10^-3^; *OphtalmoLaus* mean±SD=1.034±6.0×10^-3^.

### Stratified DF analysis: sex, age and vessel type

**
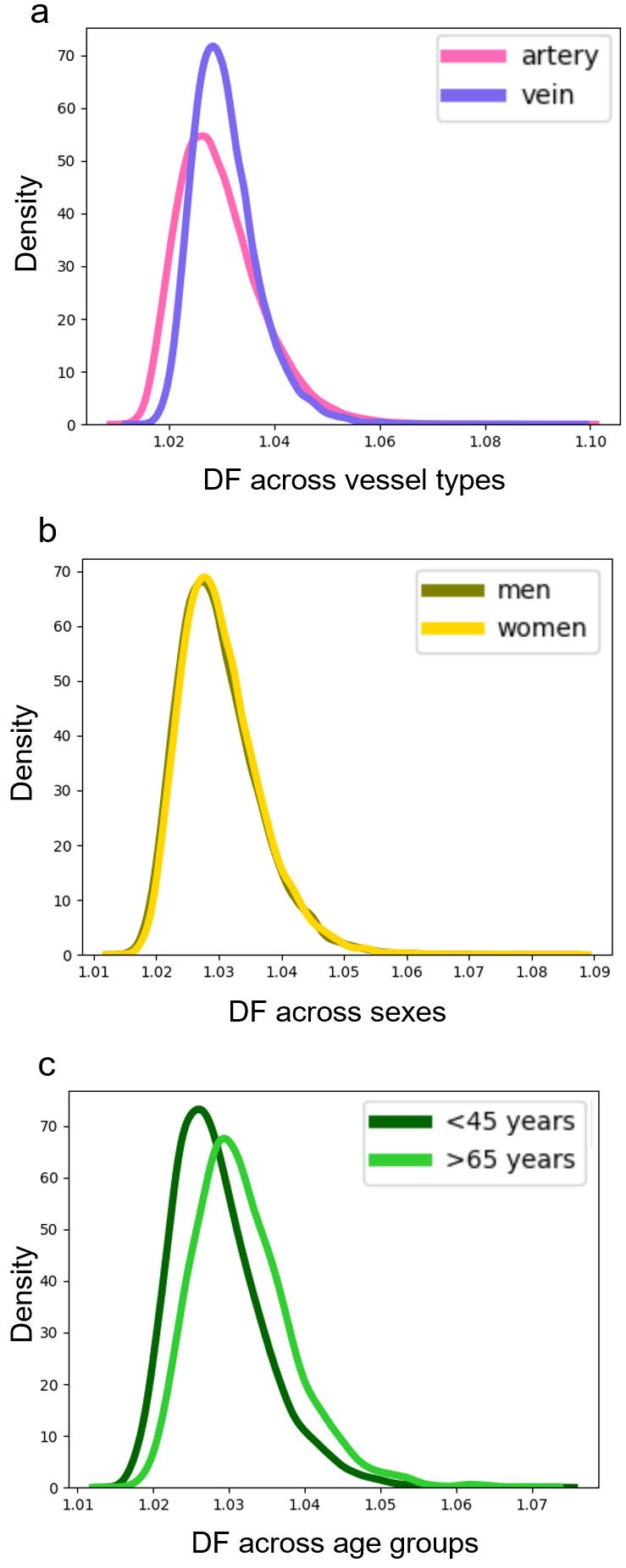
**

**Supplemental** [Figure 6](#figur_DF_hist_stratified) **| Median DF between age groups, sexes and vessel types in the Uk Biobank.**

**a**. distribution in arteries (DF = 1.029) and veins (DF = 1.030); Cohen’s d = 0.13, *p =*9×10^-142^.

**b**, distribution in men (DF = 1.0300) and women (DF = 1.0304); Cohen’s d = 0.049, *p =*9×10^-10^.

**c**, median DF increases as a function of age. In the youngest decile (<45 years) DF = 1.028, while in the oldest decile (>65 years) DF = 1.031. Cohen’s d = 0.49, *p =*1×10^-1^

## Text 4: Image segmentation and deep learning classification of arteries and veins

### Image processing methods

ARIA [(Bankhead et al. 2012)](https://paperpile.com/c/4pBVkZ/9XWqH) is a classical image processing program using wavelet transforms to automatically binarize raw fundus images into a pixel-wise mask of vessel and background. It was benchmarked on the well-established DRIVE data set, where it achieved an accuracy of approximately 94%. ARIA skeletonizes the binary mask into vessel centerlines, and cuts the skeletons at branches. This results in a collection of vessel segments, of which we know pixel-wise position, and a corresponding diameter measurement at each position. This allowed us to compute tortuosity statistics directly from ARIA output.

We performed a Github search to look for ready-to-use software to perform artery-vein classification on fundus images (time of search: summer 2020). At the time, LWNET [(Galdran et al. 2020)](https://paperpile.com/c/4pBVkZ/kcuJc) was the only software that worked flawlessly, achieved competitive performance (artery-vein classification DICE score = 96.7 on DRIVE), and provided training weights on multiple public ground truth datasets. LWNET uses a variation of the UNET architecture, which is commonly used in medical image segmentation, and results in a pixel-wise mask of 3 categories: artery, vein, background.

### Accuracy of vessel type classification

LWNET [[2]](https://paperpile.com/c/4pBVkZ/kcuJc) converts raw RGB fundus images into a categorical image of three categories, which are defined by the following RGB values: 1) artery: red (255,0,0), 2) vein: blue (0,0,255) and 3) background: black (0,0,0) (Supplemental [Figure 7](#fig_AUC_AV)a).

We used LWNET to perform automatic artery-vein classification on our 44 Skipogh ground truth images (fundus images for which we have manual annotation by ophthalmologist HA), and subsequently extracted the resulting categories for vessel segment centerlines, which we previously computed using ARIA software. For all the centerlines of the 44 images we computed a segment score based on individual centerline pixel classifications as follows:

$$S= (\Sigma red-\Sigma blue)/N_{pixel}$$

A ROC curve and its derived AUC measure was computed on the resulting vector of segment scores using logistic regression. This resulted in AUC=0.93. Accuracy was computed simply as:

$$acc= T/(T+F)$$

where *T* is the number of correctly categorized segments, and *F* the number of wrongly categorized segments. We chose not to censor any segments, and called classification based on the simple rule that vessels with S>0 were called arteries and vessels with S<0 were called veins. This resulted in *acc*= 0.88.


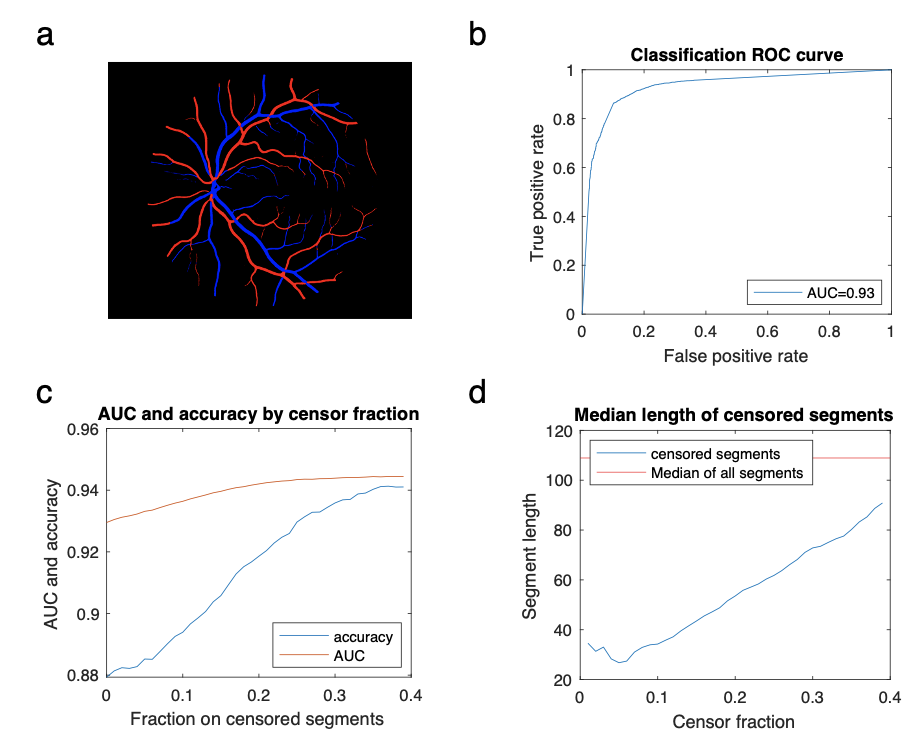


**Supplemental** [Figure 7](#figur_AUC_AV) **| AUC and accuracy of artery-vein classification.** **a.** LWNET output image consists of 3 categories: artery, vein and background. **b.** ROC curve when not censoring any segments, resulting in AUC=0.93 and accuracy=0.88 in Skipogh. **c.** AUC and accuracy could be further improved by censoring the most unclearly classified vessel segments. **d.** Median length of censored segments is significantly shorter than the overall median length, which could be the reason why we found decreased heritability in our traits in the censoring approach.

### Censoring unclearly classified vessels

Performance could be further increased by removing segments with more uncertain scores (i.e. close to zero). We increasingly removed the vessel segments with the lowest absolute score, and measured how this influenced AUC and accuracy based on the remaining segments. We found that, while AUC shows only moderate increase, accuracy increased from 0.88, without censoring, to almost 0.94 when removing a third of the segments (Supplemental [Figure 7](#fig_AUC_AV)c). However, we also found that by doing this we predominantly removed shorter segments (see Supplemental [Figure 7](#fig_AUC_AV)d).

We then ran a GWAS based on only the approximately ⅔ most confidently scored segments in the UK Biobank, and found a doubling of the number of Genome-Wide-significant hits for arteries (+117%) (the amount of signal for veins was, on the contrary, not affected). Further analysis, though, showed an inflation in the Q-Q plot of the artery-specific GWAS based on high-confidence vessels only. This was confirmed by analysis of the parameters of the LD Score Regression, which indicated a loss in the ability of the results to explain (SNP)-based heritability of the trait (h_SNP_^2^ dropped from 0.25 to 0.11), coupled with genomic inflation (intercept had increased from 1.01 to 1.93). For these reasons, all vessels identified as arteries or veins were used in the respective vessel-type-specific analysis, as selecting vessels with the highest identification score had brought marginal improvement to the already high AUC at the price of introducing a bias.

### GWAS with random vessel type calling

We estimated the independence of the signals arising from the above-described classification of arteries and veins as follows: we modified the pipeline to perform random calling of arteries and veins (by shuffling the vector of artery and veins scores computed for each eye). We then compared the similarity in the signal between two random vessel-type GWAS: we clearly show that the effect sizes (which are significant in at least one of the two GWAS) are nearly identical (*r*=0.99, *p*=4·10^-82^) when the vessel type calling is random. By comparison, the effect sizes are much less coupled (*r*=0.76, *p*=1·10^-20^) in the artery- and vein-specific GWAS based on the vessel type calling procedure that has been described above.


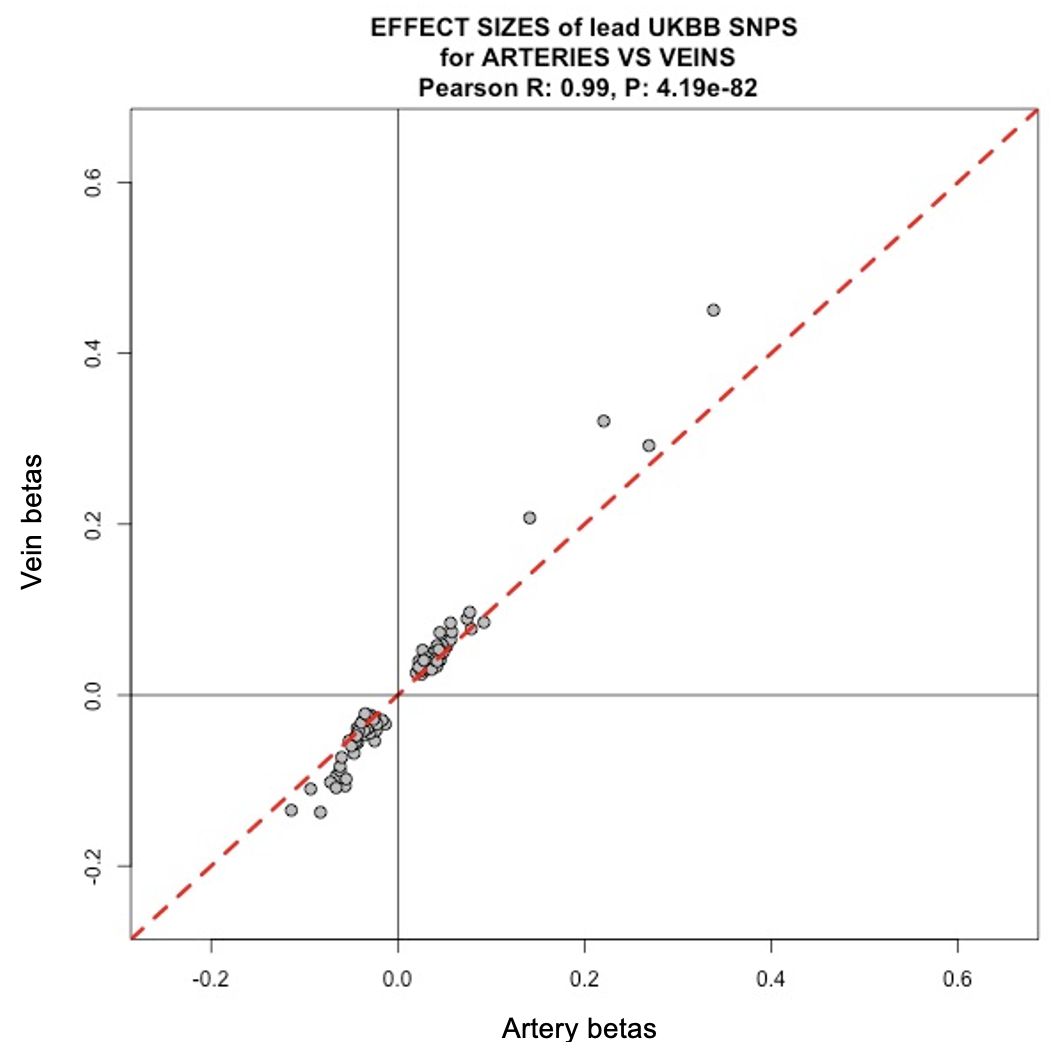

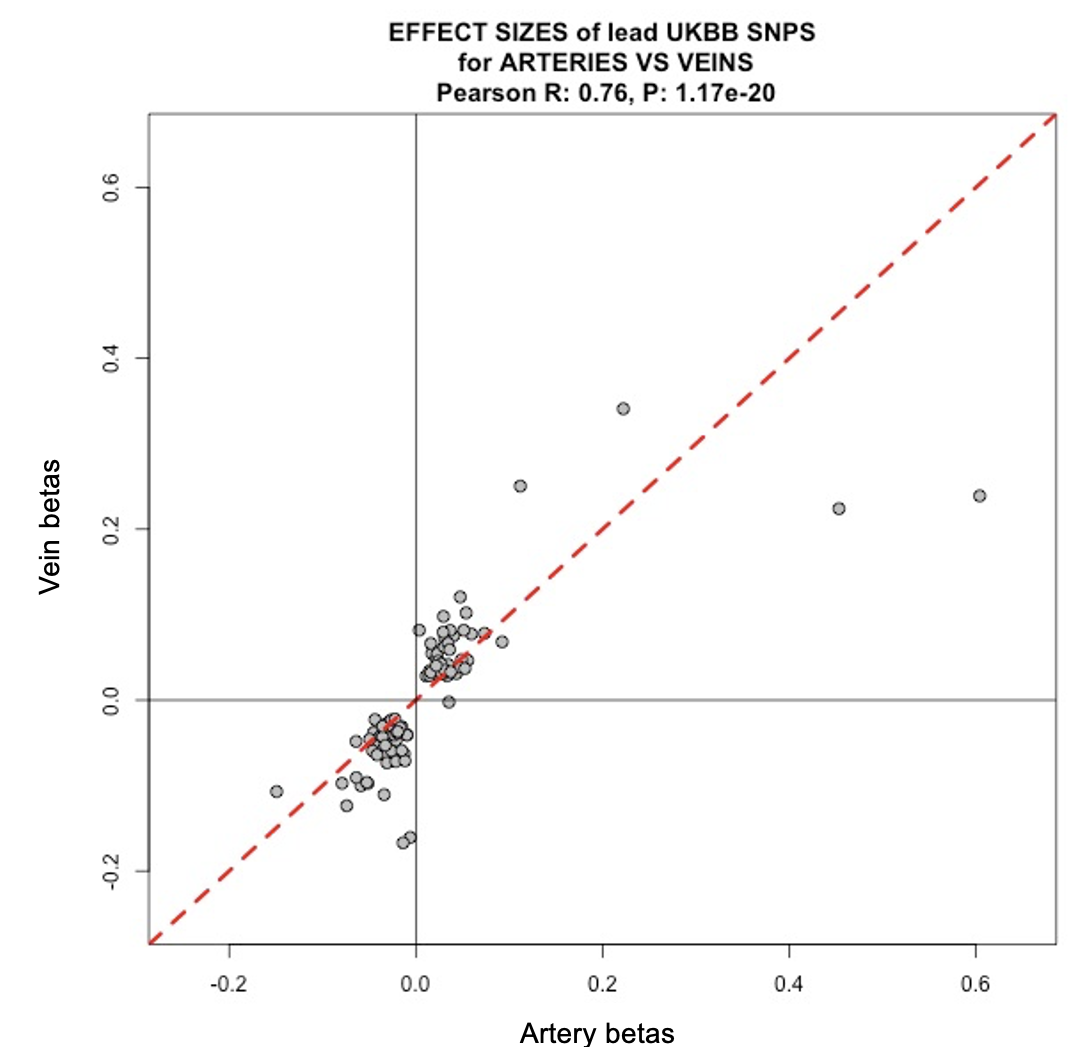


**Supplemental** [Figure 8](#figur_av_rand_beta) **| Correlation in the effect sizes from arteries and veins.** To support the effectiveness of our vessel type calling procedure, we performed random calling of arteries and veins (by shuffling the vector of artery and veins scores computed for each eye). We then performed two random vessel-type GWAS (left) and compared the results with the artery and vein-specific GWAS (right): we clearly show that the effect sizes (which are significant in at least one of the two GWAS) are less coupled when the vessel type is not random.

## Text 5: Replication Analysis

### Power calculations

The table below shows the expected number of hits depending on effect size, minor allele frequency, and number of performed tests in function of sample size. As the average absolute effect size of our discovered SNPs is 0.067, and around 100 candidates to replicate (second row in table), we had little power, and therefore did not expect to replicate any hits at our replication sample size of 1000.


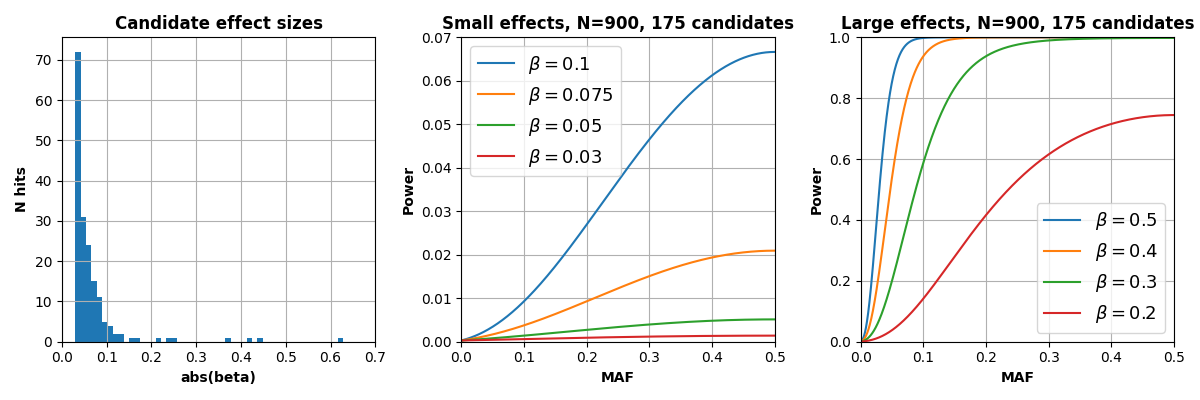


Supplemental [Figure 9](#figur_power) | Left: Distribution of absolute effect sizes of the lead SNPs identified by the discovery cohort. Center and right: Power calculations, showing expected power with Bonferroni correction for 175 candidates and replication sample size of roughly 900 as a function of the minor allele frequency for different effect sizes.

### Correlation of effect sizes in the meta-cohort

Of the 136 SNP shared between the discovery (UKBB) and replication studies (SKIPOGH plus *OphtalmoLaus*), the sign of the effect sizes was concordant in 90 (binomial test p = 5.0×10-5). We observed a Pearson correlation of *r*= 0.53 (*p*= 1.2×10^-11^) between the effect size estimates in the two studies. When some outliers are removed, the correlation drops from *r*= 0.53 to *r*= 0.36, remaining highly significant (see Supplemental Figure 10a).

The UKBB and SKIPOGH genotyping data may not be directly comparable, since the fact that the imputation methods were different might inflate the results) We propose a targeted analysis to show that the effect sizes between UKBB and SKIPOGH (alone) are significantly correlated (see Supplemental Figure 10b).


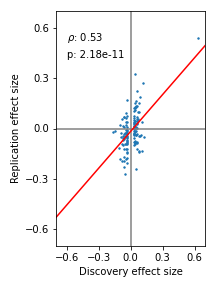

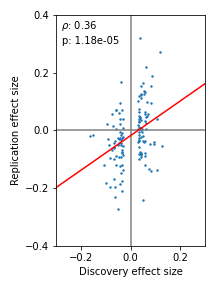


**a.** Discovery (UKBB) and replication cohort (SKIPOGH plus *OphtalmoLaus*)

#


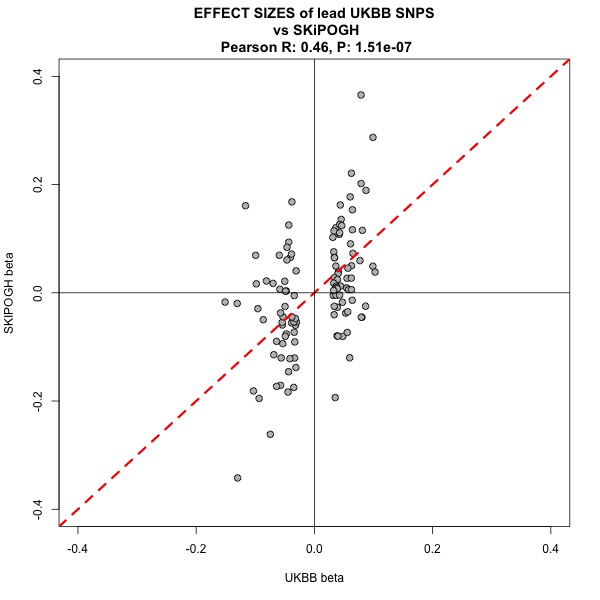

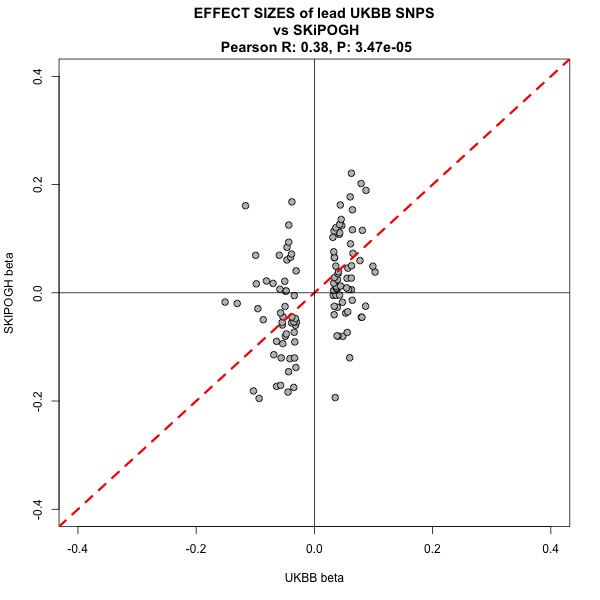


**b.** Discovery (UKBB) and and SKIPOGH cohort

**Supplemental** [Figure 10](#figur_replication_skipogh) **| Extended plot for correlation of effect sizes between discovery and replication cohort.** **a,** Left: correlation of effect sizes in the discovery (UKBB) and replication cohort (SKIPOGH plus *OphtalmoLaus*). Right: same plot without considering the outlier. We removed one outlier in the top-right quadrant, corresponding to rs187691758. This shows the robustness of the result: even though the Pearson correlation dropped from *r*=0.53 to *r*=0.36, it remained highly significant (p=1.18·10^--5^). Out of the 135 remaining SNPs, 89 shared the sign in effect size (binomial test p=​​2.94·10^-6^). **b,** Left: correlation of effect sizes in the discovery and SKIPOGH cohort. Right: the same plot, without considering weak outliers. We removed two outliers in the bottom-left quadrant: (-0.12986,-0.3421) corresponding to rs77218478, (-0.074573, -0.2615) corresponding to rs79297533; and two outliers in the top-right quadrant: (0.078949,0.3656) corresponding to rs72950114 and (0.09906, 0.2872) corresponding to rs138138767. This shows the robustness of the result: even though the Pearson correlation dropped from *r*=0.46 to *r*=0.38, it remained highly significant (*p*=3.47E-5).

### Replication of hits in the meta-cohort

We performed a meta-analysis of the two cohorts OphtalmoLaus (N=514) and SKIPOGH (​N=​397), using a fixed-effects model (see below). For each SNP, the fixed-effects model computes meta-values of the standard error (SE) and effect size summary statistics, with the meta effect size being a weighted average of individual effect sizes, weighted by their corresponding inverse SE:

$\beta_{meta}=\frac{\sum_{i}^{M} w_{i}\times{\beta_{i}}}{\sum_{i}^{M} w_{i}}$ $SE_{meta}=\frac{1}{\sqrt{\sum_{i}^{M} w_{i}}}$ where $w_{i}=\frac{1}{S{E_{i}}^{2}}$, and M the number of cohorts

The resulting SNP P-value is then given by a two-tailed t-statistic

$t=\frac{\beta_{meta}}{SE_{meta}}$ with $\sum_{i}^{M} N_{i}-1$ degrees of freedom (911 in our case)

All replication is performed on the combined-vessel median distance factor phenotype.


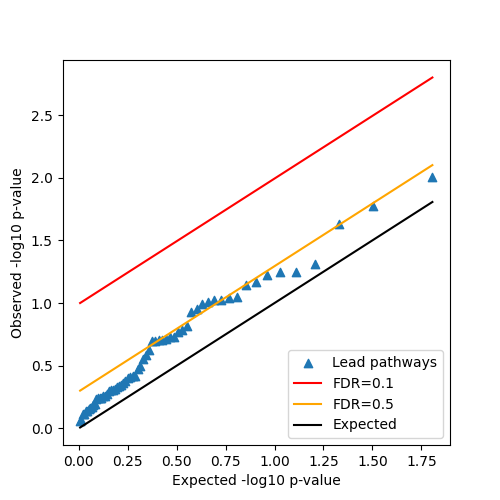


**Supplemental** [Figure 11](#figur_colski_fe) **| Replication meta-analysis with fixed-effects.**

Meta-GWAS summary statistics were obtained using the **fixed-effects** model described above. SNP-level and gene-level summary statistics were reported in the main text. SNP-level summary statistics were aggregated into pathway-level p-values using *PascalX*. Benjamini-Hochberg (BH) procedure on significant pathways from the discovery cohort doesn’t replicates two pathways: GO_REGULATION_OF_ANATOMICAL_STRUCTURE_MORPHOGENESIS, and ZNF257_TARGET_GENES

# SUPPLEMENTAL RESULTS

## Text 6: Baseline Characteristics

| **Age (mean±SD)** | 56±8 years |
| --- | --- |
| **Sex (females at birth)** | 35 098 (54%) |
| **Smokers (mean±SD)** | 4 618 (7%) |
| **BMI (mean±SD)** | 27±5 kg/m^2^ |
| **SBP (mean±SD)** | 140±20 mmHg |
| **DBP (mean±SD)** | 82±11 mmHg |
| **Ethnicity** | 54 343 (94%) self-reported ethnicity as White, 1 243 (2.1%) as Asian, 962 (1.7%) as Back, 373 (0.6%) as Mixed, 175 (0.3%) as Chinese, and 521 (0.9%) as Other |

**Supplemental** [Table 2](#table_BaselineCharacteristics) **| Baseline characteristics in the UK Biobank.**

| **Vascular diseases** | **Type 2 diabetes** | **Angina** | **Myocardial infarction** | **Deep-vein thrombosis** | **Stroke** | **Stage 2 hypertension** |
| --- | --- | --- | --- | --- | --- | --- |
| **Number of cases** | 2 644 | 1 448 | 1 077 | 1 072 | 750 | 8 797 |
| **Ocular diseases** | **Glaucoma** | **Macular degeneration** | **Diabetes related eye disease** | **Injury or trauma resulting in loss of vision** | **Cataract** | **Other serious eye condition** |
| **Number of cases** | 935 | 593 | 575 | 353 | 2415 | 1050 |

**Supplemental** [Table 3](#table_baseline_diseases) **| Baseline disease characteristics in the UK Biobank.** Deep-vein thrombosis refers to a blood clot in the leg. Stage 2 hypertension refers to automated reading of blood pressure >90 mmHg diastolic or >140 mmHg systolic.

Across the analyzed individuals, baseline characteristic of the UK Biobank can be found in [table 2](#tab_BaselineCharacteristics). Among the participants for which at least one retinal fundus image was available.

Among the participants for which at least one retinal fundus image was available, the number of cases (i.e., people who has been diagnosed) for vascular-related and ophthalmological diseases can be found in [table 3](#tab_baseline_diseases).

## Text 7: Correlation with disease status

We built a logistic regression classifier and found retinal vein tortuosity to have predictive power over disease outcome: angina AUC 55.2%, heart attack AUC 53.4%, stroke AUC 54.6%, Deep Vein Thrombosis (DVT) AUC 53.3% and hypertension AUC 56.6%. To determine whether retinal tortuosity might be used as an independent biomarker for CVD, we trained logistic regression models with known risk factors: age, sex, SBP and smoking (pack years): angina AUC 76.2%, heart attack AUC 80.6%, stroke AUC 69.3%, DVT 61.8%, The same procedure was applied to the prediction of hypertension, but without using SBP as a risk factor, resulting in AUC 75.4%. These risk factors models did not significantly increase in performance by adding any of the median vessel tortuosity measures: we conclude that, despite associations with CVD outcome, retinal tortuosity does not represent an increased health risk after correcting for known risk factors. The analysis was performed on all vessels, then repeated only on veins and only on arteries. Results varied slightly. To illustrate this, we show the distributions of median vessel tortuosity in hypertensive patients vs. controls.


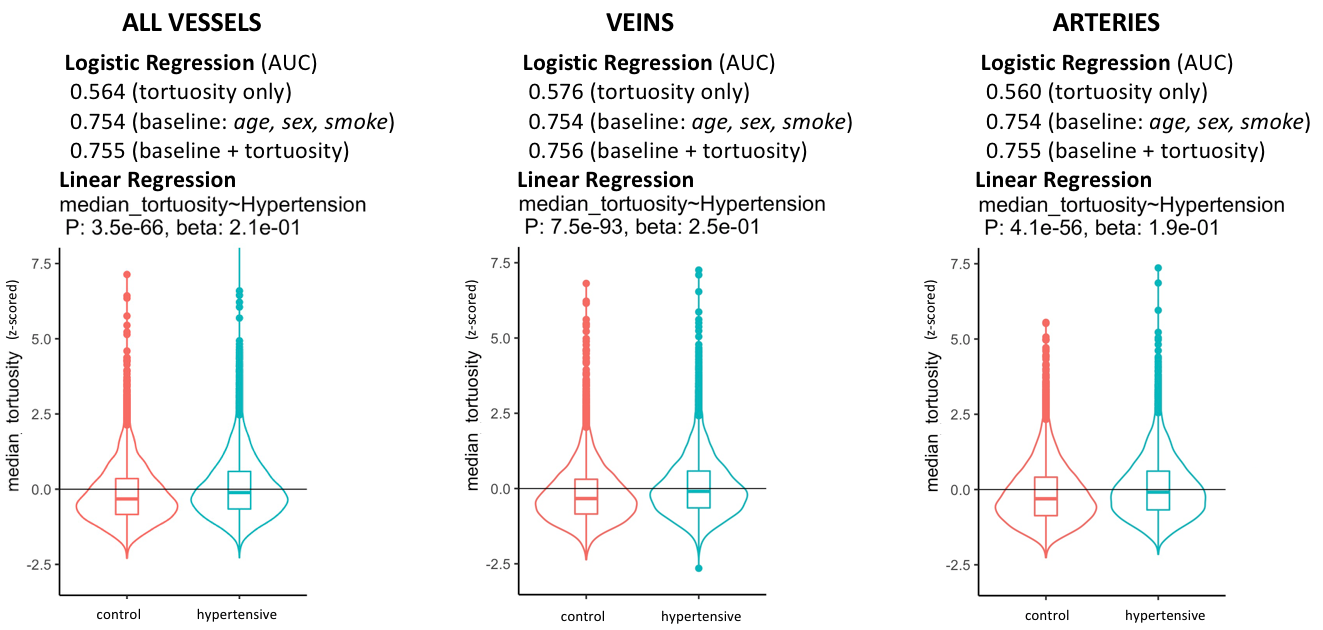


**Supplemental** [Figure 13](#figur_predict_disease) **| Predictive power of median tortuosity over hypertension.** Effects and p-values are calculated using linear regression. AUC refers to logistic regression.

## Text 8: Replication and confounders

### Replication of known hits

We replicated two known associations. We failed to replicate a third, for which association was controversial (it was only marginally significant in its discovery cohort and had failed replication in the independent cohort of the study that originally proposed it).

| **RSID** | **REPLICATED**  **in our GWAS** | **REPLICATED**  **in its own study** | **POSITION** | **GENE(S)** | **trait** |
| --- | --- | --- | --- | --- | --- |
| rs1808382 | yes | yes | 19: 38.7 Kb | ACTN4 / CAPN12 | retinal venular tortuosity |
| rs7991229 | yes | yes | 13: 111.1 Kb | COL4A2 | retinal arteriolar tortuosity |
| rs73157566 | no | no | 12: 129.5 Kb | NLRP9P1 | retinal venular tortuosity |

**Supplemental** [Table 4](#table_known_associations) **| Known associations with retinal vessel tortuosity.** Three SNPs known to associate with a phenotype related to retinal vessel tortuosity in the literature[[4]](https://paperpile.com/c/4pBVkZ/Ttc9g). Details can be found in the list of all statistically significant SNPs (see Supplemental Dataset 1A/[1B](https://docs.google.com/spreadsheets/d/1ei2pogiEswGmz36-dXvMa61Kd4vFvVDVkCcIuqG43po/edit?usp=sharing)/[1C](https://docs.google.com/spreadsheets/d/1Q0tyyNL7lOfLIqX4nVHbIJdJpGFrKCV3M8MakI2zaa8/edit?usp=sharing)).


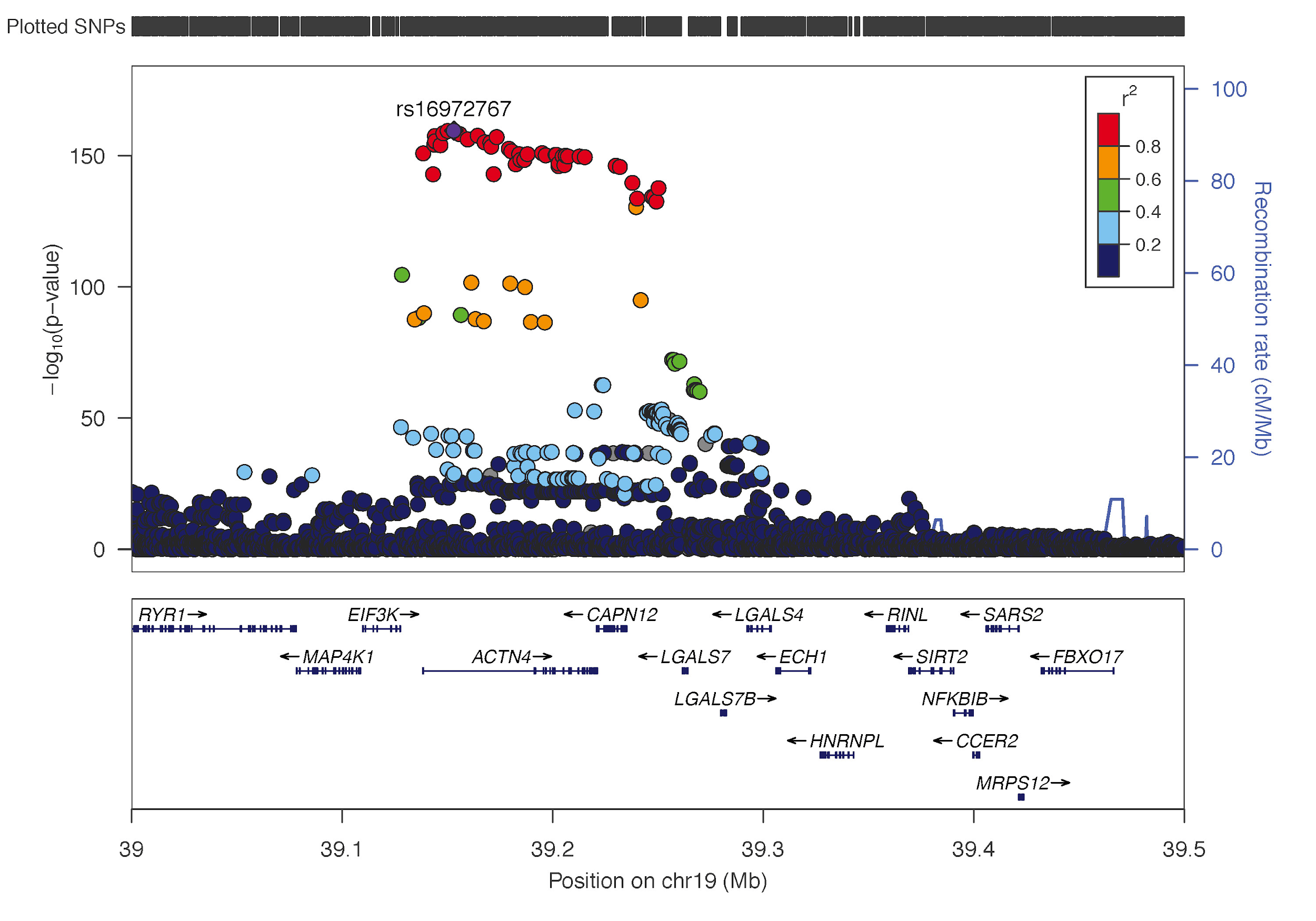


**Supplemental** [Figure 14](#figur_locuszoom1) **| LocusZoom of known associations with rs1808382.** Although we did not recover the exact rsid variant, we report a number of exonic variants (more likely to be causative) in extremely strong LD, which represent our strongest signal: in particular, we recovered a variant in perfect LD, rs16972767, which is an intron variant for ACTN4, the gene on which rs1808382 was reported as having potential direct regulatory effects[[4]](https://paperpile.com/c/4pBVkZ/Ttc9g). rs16972767 is reported in the literature as a venular tortuosity hit: in accordance with this, its significance was higher in our venular tortuosity GWAS (-log_10_ p=165) rather than in our arteriolar tortuosity GWAS (-log_10_ p=65).


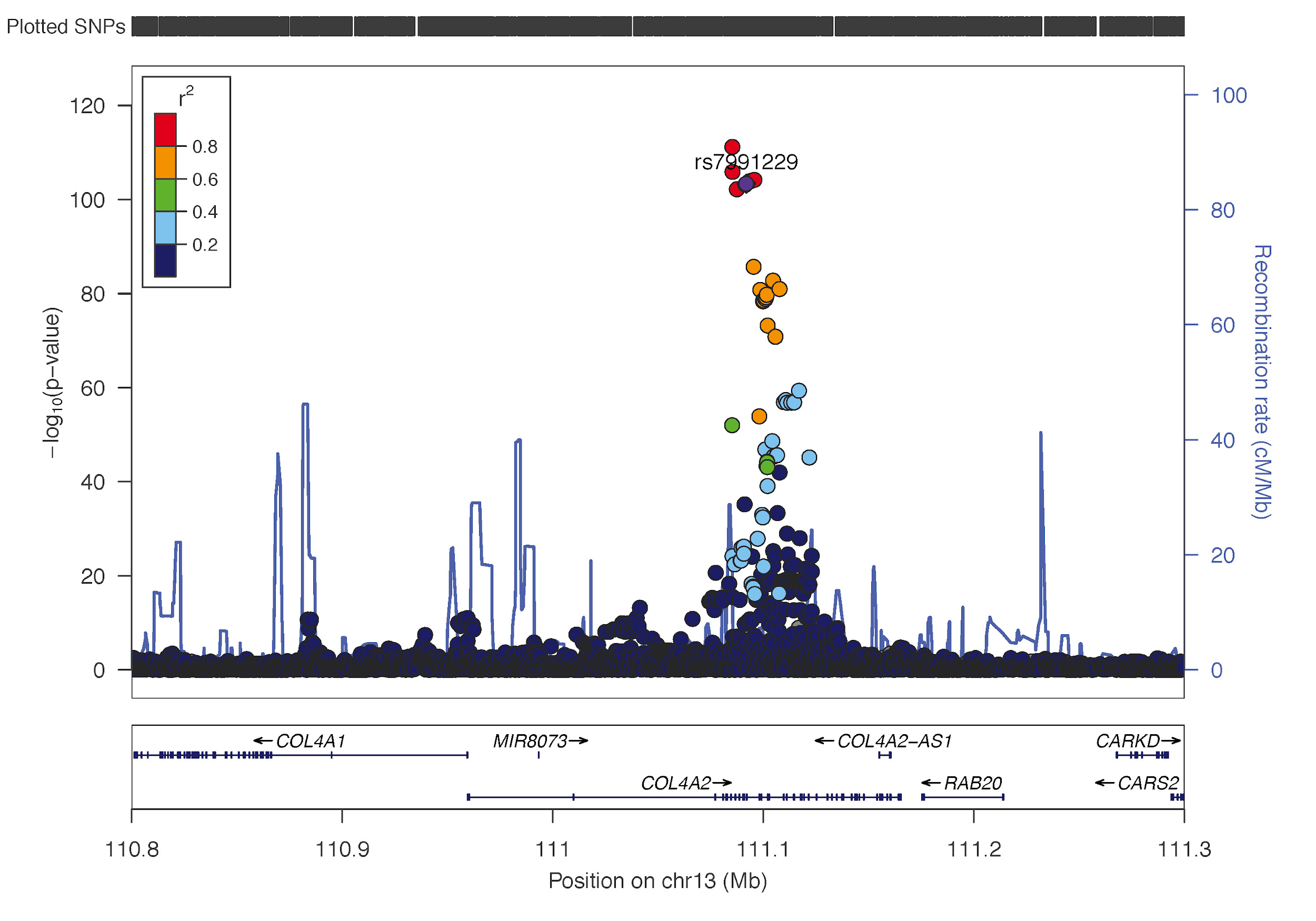


**Supplemental** [Figure 15](#figur_locuszoom2) **| LocusZoom of known associations with rs7991229.** This SNP was reported in the literature as an association to arteriolar tortuosity: indeed, its significance was substantially higher in our artery tortuosity GWAS (-log_10_ p=166) than in our vein tortuosity GWAS (-log_10_ p=8).


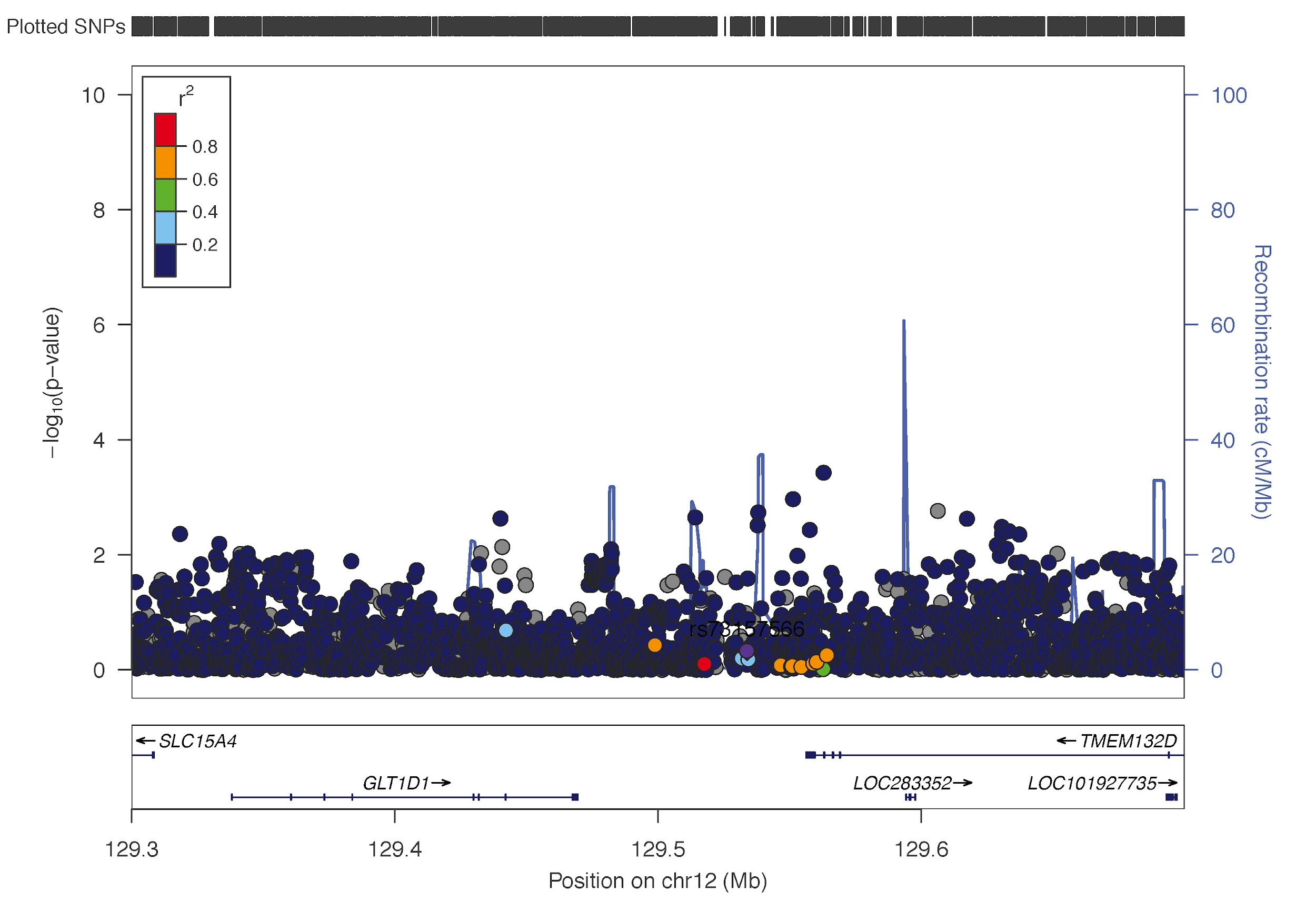


**Supplemental** [Figure 16](#figur_locuszoom3) **| LocusZoom of (controversial) known associations with rs73157566.** We did not reproduce it. Given the fact that this association had not reached genome-wide significance in its own replication cohort, we propose this locus should not be considered as associated retinal vessel tortuosity.

### Variance explained by major confounders vs. genetics

To compare the proportion of variance in distance factor (DF) tortuosity explained by or GWAS – estimated by ${h^{2}}_{SNP}$ – to the amount explained by major confounders, we calculated $R^{2}$ of the following linear models:

| **Model** | $\boldsymbol{R}^{\boldsymbol{2}}$ |
| --- | --- |
| 1. DF ~ age | 2.7% |
| 1. DF ~ spherical power | 0.5% |
| 1. DF ~ age + spherical power | 3.0% |
| 1. DF ~ age + sex + spherical power + PCs (1, 2, 5, 6, 7, 8, 16, 17, 18) | 4.1% |

In comparison, performing GWAS on the residuals of model 4, we estimated heritability of DF tortuosity at ${h^{2}}_{SNP}=25\%$, thus the SNPs in our GWAS explain a total of (1-4.1%)*25%=24% of the phenotypic variance of the DF, a proportion much larger than all the covariates combined.

## Text 9: Vessel-type comparisons for SNPs, Genes and Pathways

**1) SNPs**


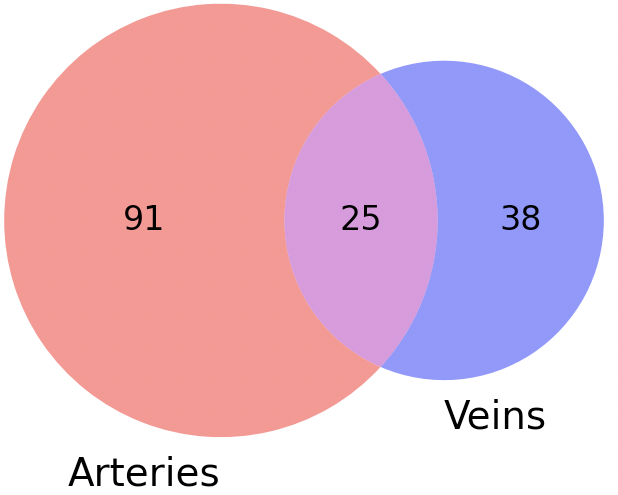

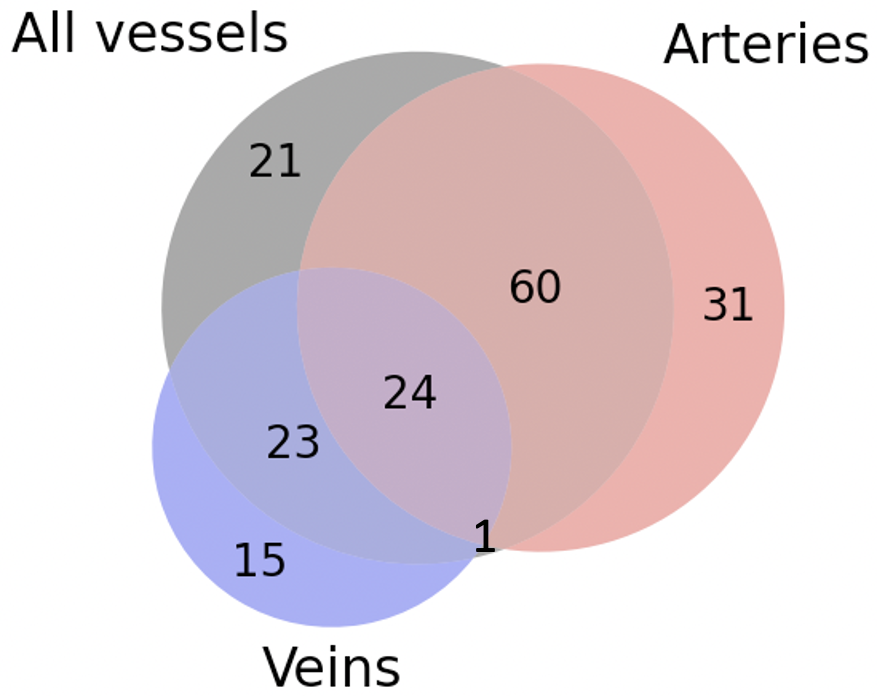


**________________________________________________**

**2) Genes**


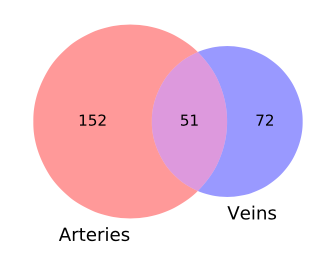

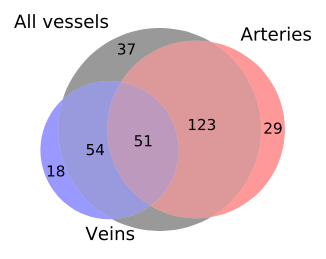


**________________________________________________**

**3) Pathways**


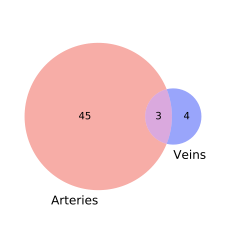

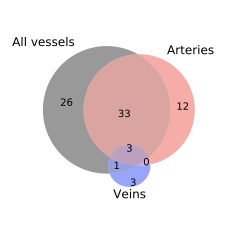


**Supplemental** [Figure 17](#figur_coparative_AV) **| Venn diagrams.** Overlap between Bonferroni-thresholded 1) SNPs, 2) genes and 3) pathways across phenotypes. Left: **Hits exclusive to artery GWAS *vs.* vein GWAS:** Venn diagram showing overlap between significant 1) SNPs, 2) genes, 3) pathways in arteries and veins. We find overall more significant SNPS, genes and pathways in arteries, and also more than twice as many artery-exclusive SNPs, genes and pathways than vein-exclusive. Right: **Hits exclusive to artery GWAS *vs.* vein GWAS *vs.* all-vessels (combined-vessel-type GWAS):** Combining arteries and veins into a single “all vessels” phenotype catches many of the SNPs, genes and pathways detected by the artery and vein phenotypes and also provide some news

## Text 10: Genetic associations with disease and risk

### Tortuosity variants associated with disease outcome

| **SHARED SNP** | **ref** | **DISEASE GWAS** |
| --- | --- | --- |
| rs3184504 | [[5]](https://paperpile.com/c/4pBVkZ/d4FtO) | Coronary heart disease |
| rs2472299 | [[6]](https://paperpile.com/c/4pBVkZ/Xbhsn) |  |
| rs10483727 | [[7]](https://paperpile.com/c/4pBVkZ/tidye) | Glaucoma |
| rs3184504 | [[8]](https://paperpile.com/c/4pBVkZ/ykYon) |  |
| rs35155027 | [[8]](https://paperpile.com/c/4pBVkZ/ykYon) |  |
| rs10483727 | [[7]](https://paperpile.com/c/4pBVkZ/tidye) |  |
| rs34935520 | [[7]](https://paperpile.com/c/4pBVkZ/tidye) |  |
| rs10774625 | [[9]](https://paperpile.com/c/4pBVkZ/s22tj) | Hypertension |
| rs6495127 | [[9]](https://paperpile.com/c/4pBVkZ/s22tj) |  |
| rs3184504 | [[10]](https://paperpile.com/c/4pBVkZ/gNnhZ) | Type 1 diabetes |
| rs3184504 | [[11]](https://paperpile.com/c/4pBVkZ/4rhsL) |  |
| rs653178 | [[12]](https://paperpile.com/c/4pBVkZ/x7QKM) | Chronic kidney disease |
| rs653178 | [[13]](https://paperpile.com/c/4pBVkZ/ec8Mm) | Celiac disease |
| rs4766578 | [[12]](https://paperpile.com/c/4pBVkZ/x7QKM) | Heart failure |
| rs4766578 | [[14]](https://paperpile.com/c/4pBVkZ/33U5Y) | Vitiligo |
| rs1129038 | [[14]](https://paperpile.com/c/4pBVkZ/33U5Y) |  |
| rs653178 | [[12]](https://paperpile.com/c/4pBVkZ/x7QKM) | Chronic kidney disease |

**Supplemental** [Table 5](#table_disease_outcome1) **| Variants associated with disease.** List of variants influencing both tortuosity and disease outcome without statistically significant association to a gene.

| **SHARED SNP** | **ref** | **DISEASE GWAS** |
| --- | --- | --- |
| rs78058190 | [[15]](https://paperpile.com/c/4pBVkZ/3iVgy) | Cardiovascular diseases |
| rs10942863 | [[15]](https://paperpile.com/c/4pBVkZ/3iVgy) |  |
| rs62434109 | [[15]](https://paperpile.com/c/4pBVkZ/3iVgy) |  |
| rs3184504 | [[15]](https://paperpile.com/c/4pBVkZ/3iVgy) |  |
| rs11072508 | [[15]](https://paperpile.com/c/4pBVkZ/3iVgy) |  |
| rs189349094 | [[15]](https://paperpile.com/c/4pBVkZ/3iVgy) |  |
| rs72938315 | [[15]](https://paperpile.com/c/4pBVkZ/3iVgy) | Respiratory diseases |
| rs7687906 | [[16]](https://paperpile.com/c/4pBVkZ/ropyf) | Obesity-related traits |
| rs3184504 | [[10]](https://paperpile.com/c/4pBVkZ/gNnhZ) | Autoimmune traits |
| rs7310615 | [[15]](https://paperpile.com/c/4pBVkZ/3iVgy) |  |

**Supplemental** [Table 6](#table_disease_outcome2) **| Variants associated with disease (general outcome).** List of variants influencing both tortuosity and general non-specific disease outcomes (e.g.,"Obesity-related traits" does not specify the exact disease outcome).

### Tortuosity variants associated with disease risk factors

| **SHARED SNP** | **ref** | **RISK FACTOR** |
| --- | --- | --- |
| rs35155027 | [[8]](https://paperpile.com/c/4pBVkZ/ykYon) | Intraocular pressure (open-angle glaucoma) |
| rs8053277 | [[8]](https://paperpile.com/c/4pBVkZ/ykYon) | Vertical cup-disc ratio (glaucoma) |
| rs3184504 | [[17]](https://paperpile.com/c/4pBVkZ/i8qhg) | Systolic blood pressure (CVD) |
| rs1378942 | [[18]](https://paperpile.com/c/4pBVkZ/iDWJd) |  |
| rs3184504 | [[17]](https://paperpile.com/c/4pBVkZ/i8qhg) | Diastolic blood pressure (CVD) |
| rs3184504 | [[18]](https://paperpile.com/c/4pBVkZ/iDWJd) |  |
| rs653178 | [[19]](https://paperpile.com/c/4pBVkZ/rDOwW) |  |
| rs653178 | [[20]](https://paperpile.com/c/4pBVkZ/edvlO) | Mean arterial pressure (CVD) |
| rs597808 | [[15]](https://paperpile.com/c/4pBVkZ/3iVgy) | Waist-hip ratio (obesity) |
| rs78058190 | [[15]](https://paperpile.com/c/4pBVkZ/3iVgy) |  |
| rs4744056 | [[21]](https://paperpile.com/c/4pBVkZ/isYSy) | General cognitive ability (mental disorders) |

**Supplemental** [Table 7](#table_risk_factor) **| Variants associated with risk of disease.** List of variants influencing both tortuosity and disease outcome without statistically significant association to a gene.

## Text 11: Mendelian Randomization

| **Tortuosity -> Outcome** | | | | | **Exposure -> Tortuosity** | | | | |
| --- | --- | --- | --- | --- | --- | --- | --- | --- | --- |
| **Exposure** | **Outcome** | **Effect** | **SE** | **Pvalue** | **Exposure** | **Outcome** | **Effect** | **SE** | **Pvalue** |
| artery | BMI | 0.019 | 0.015 | 0.200 | BMI | artery | 0.028 | 0.030 | 0.356 |
| mixed |  | 0.008 | 0.015 | 0.581 |  | mixed | -0.011 | 0.029 | 0.715 |
| vein |  | -0.044 | 0.018 | 0.013 |  | vein | -0.047 | 0.027 | 0.078 |
| artery | CAD | -0.053 | 0.041 | 0.200 | CAD | artery | -0.018 | 0.028 | 0.524 |
| mixed |  | -0.061 | 0.038 | 0.109 |  | mixed | -0.008 | 0.024 | 0.748 |
| vein |  | -0.076 | 0.046 | 0.097 |  | vein | -0.002 | 0.016 | 0.924 |
| artery | SBP | -0.200 | 0.362 | 0.581 | SBP | artery | -0.017 | 0.017 | 0.324 |
| mixed |  | 0.033 | 0.344 | 0.924 |  | mixed | -0.015 | 0.015 | 0.309 |
| vein |  | -0.035 | 0.538 | 0.948 |  | vein | -0.010 | 0.009 | 0.252 |
| artery | HDL | 0.017 | 0.015 | 0.236 | HDL | artery | 0.010 | 0.008 | 0.235 |
| mixed |  | 0.014 | 0.015 | 0.351 |  | mixed | 0.011 | 0.009 | 0.193 |
| vein |  | 0.009 | 0.024 | 0.722 |  | vein | 0.008 | 0.009 | 0.383 |
| artery | LDL | -0.001 | 0.016 | 0.940 | LDL | artery | -0.046 | 0.013 | 0.001 |
| mixed |  | 0.006 | 0.017 | 0.709 |  | mixed | -0.043 | 0.013 | 0.001 |
| vein |  | 0.002 | 0.029 | 0.945 |  | vein | -0.031 | 0.013 | 0.018 |
| artery | TC | 0.000 | 0.017 | 0.991 | TC | artery | 0.000 | 0.002 | 0.947 |
| mixed |  | 0.006 | 0.019 | 0.741 |  | mixed | 0.000 | 0.002 | 0.960 |
| vein |  | -0.006 | 0.032 | 0.844 |  | vein | 0.000 | 0.002 | 0.963 |
| artery | TG | -0.006 | 0.013 | 0.656 | TG | artery | -0.038 | 0.018 | 0.038 |
| mixed |  | 0.003 | 0.013 | 0.833 |  | mixed | -0.034 | 0.016 | 0.038 |
| vein |  | -0.008 | 0.022 | 0.720 |  | vein | -0.014 | 0.014 | 0.301 |

**Supplemental** [Table 8](#table_MD) **|** **Bi-directional Mendelian Randomization results**. Causal estimates are based on the inverse variance weighted (IVW) method. Abbreviations: Body Mass Index (BMI), Coronary artery disease (CAD), Systolic Blood Pressure (SBP), High-density lipoprotein (HDL), Low-density lipoprotein (LDL), total cholesterol (TC) and triglycerides (TG).

## Text 12: ACTN4 and COL4A2 over-expression

**
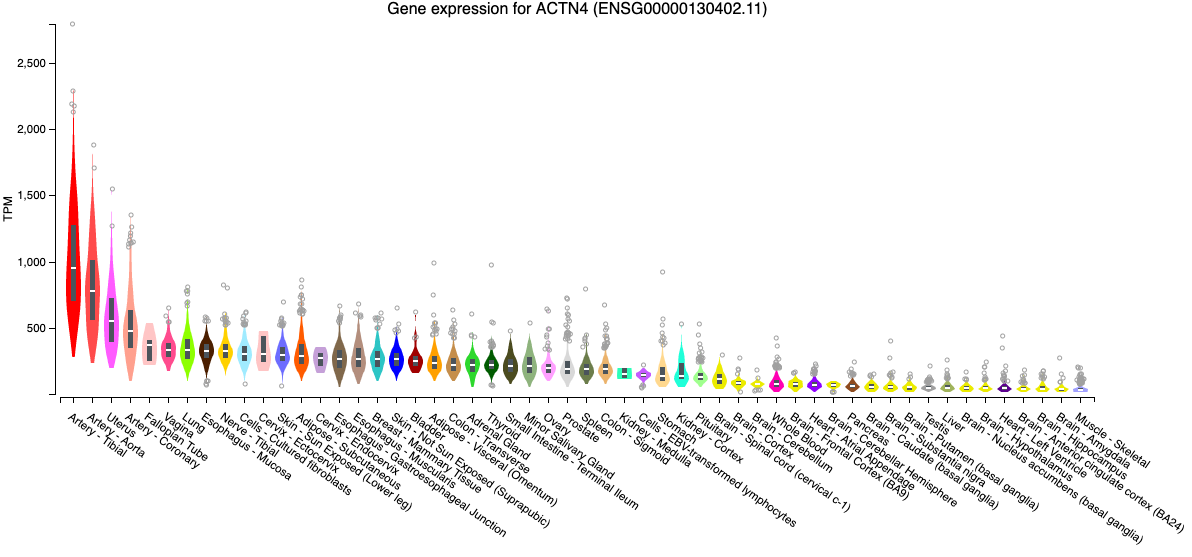
**

**
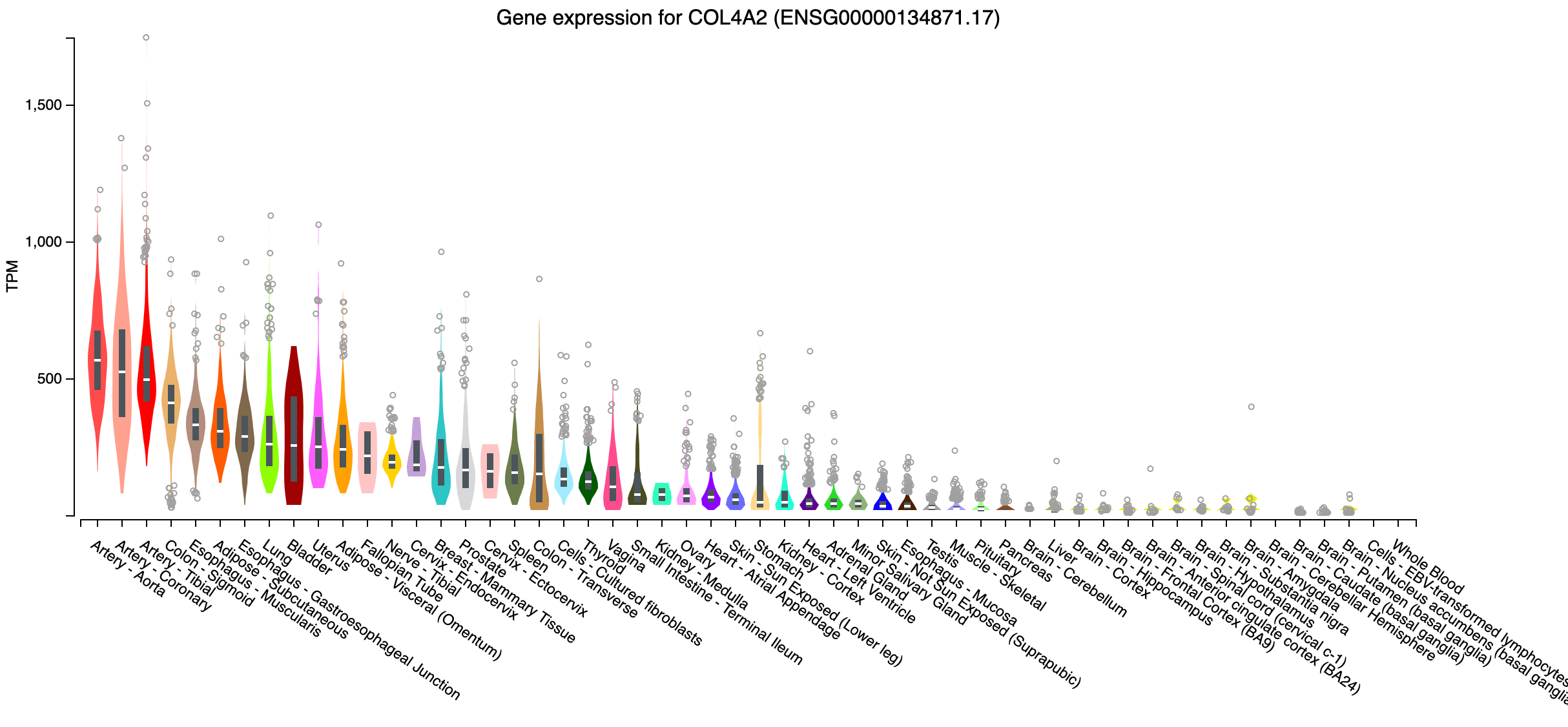
**

**Supplemental** [Figure 18](#figur_ACTN4_COL4A2_expression) **| ACTN4 and COL4A2 abundance according to GTEx.** The two highly significant genes’ ACTN4 and COL4A2 mRNAs were both found to be highly abundant in blood vessels.

## Text 13: Full gene set enrichment results
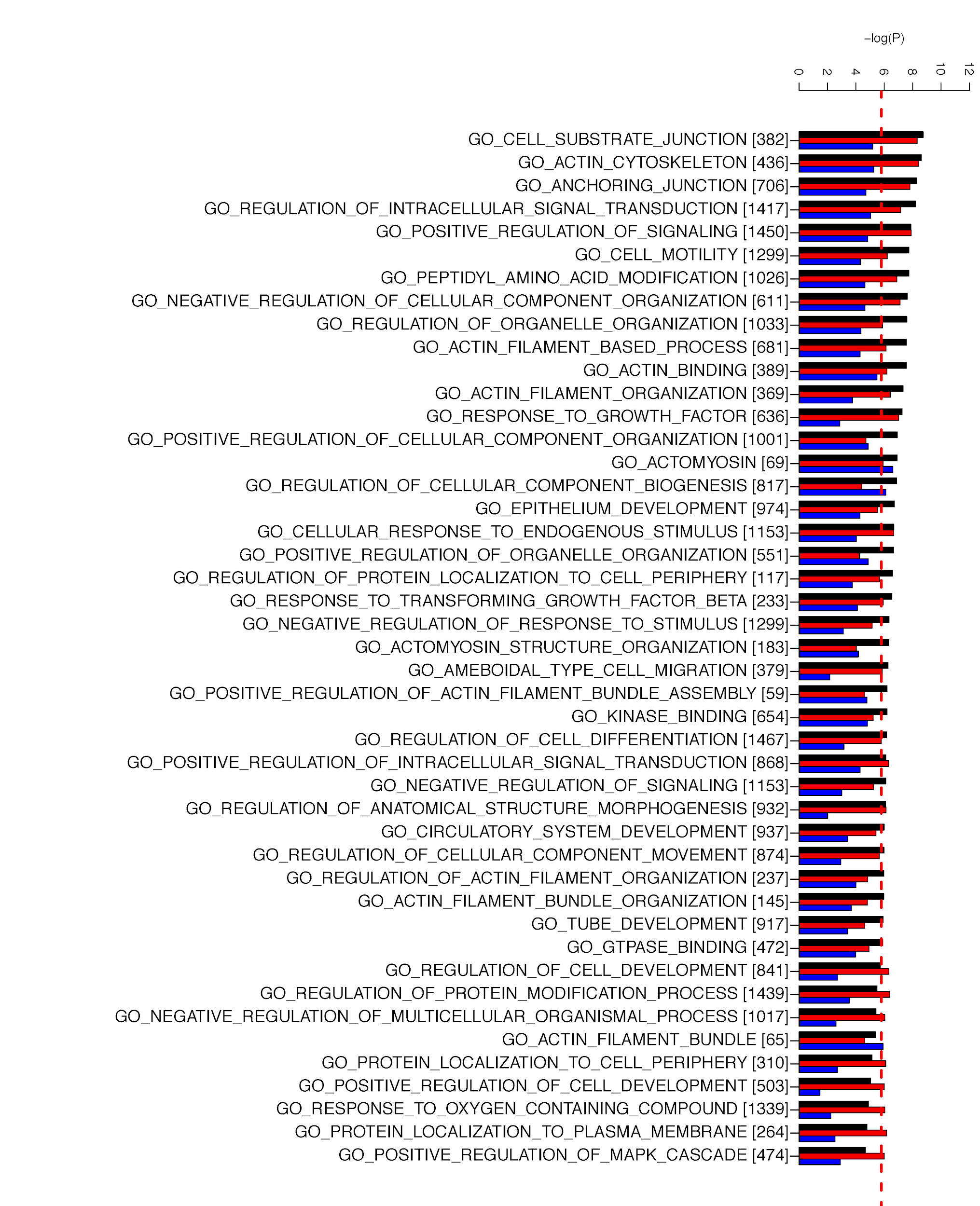


**Supplemental** [Figure 19](#figur_Overlap_GO) **| Extended version of Figure 5 from the main text (GO terms).** Extended version where all enriched GO terms are shown (irrespective of them being very general or redundant) . Additionally, label names are printed out in full. The number of genes in each set is indicated in squared brackets.


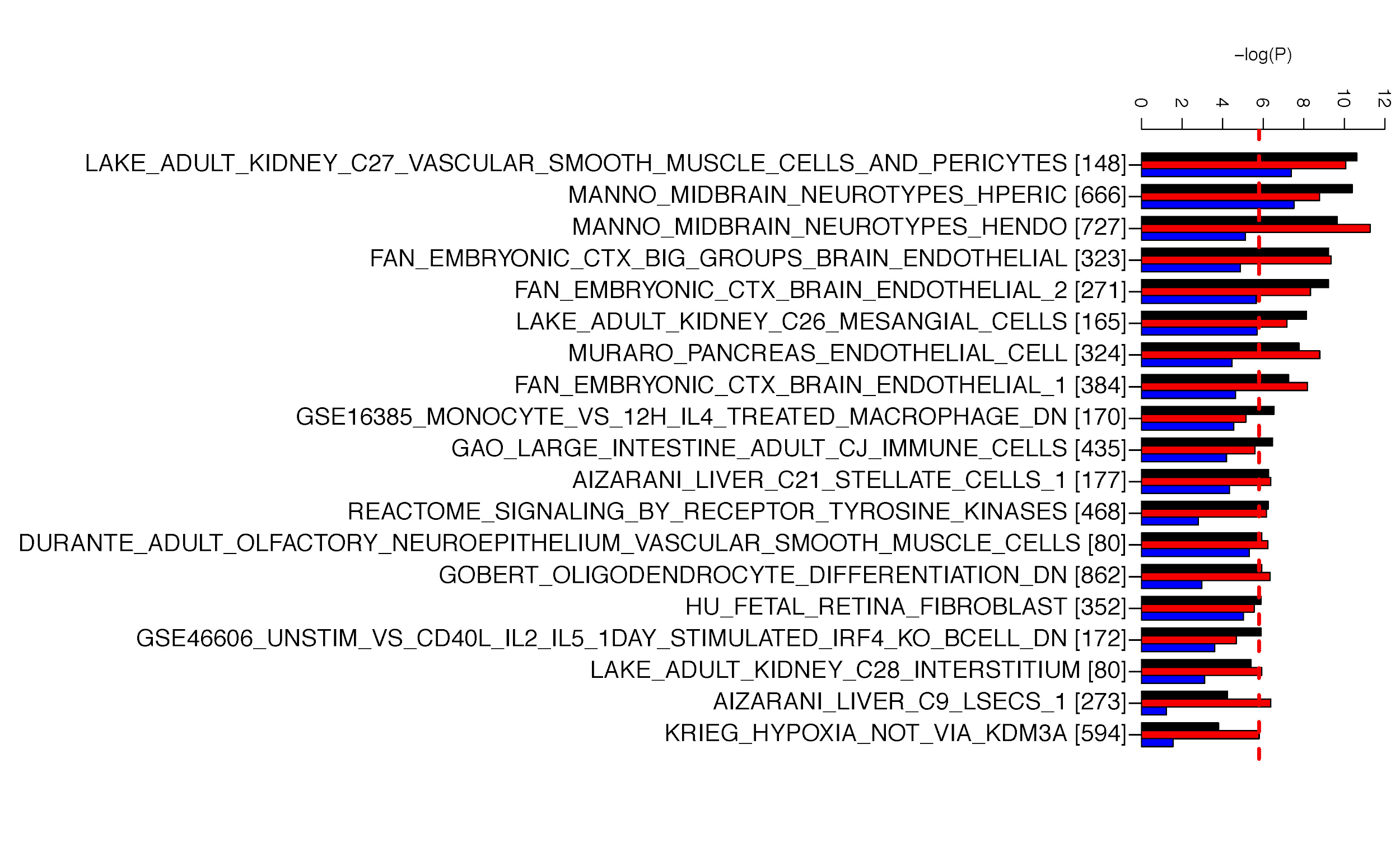


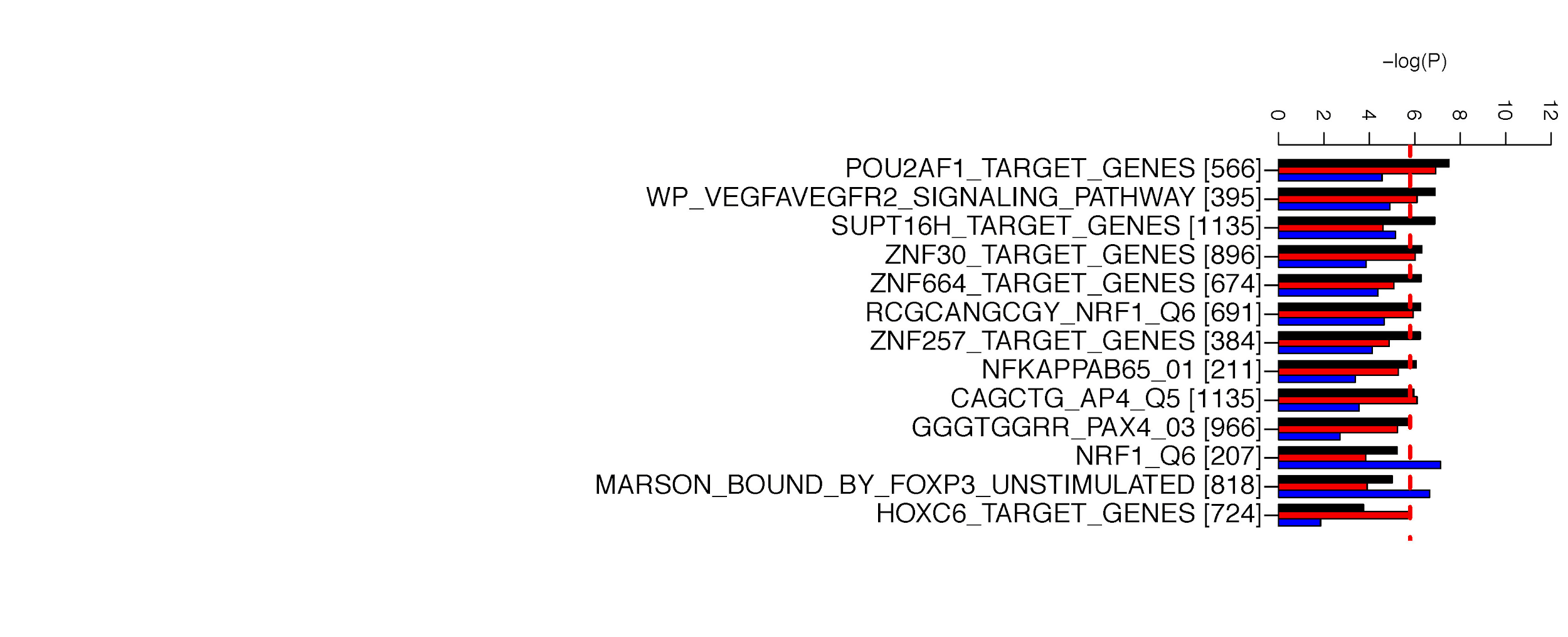


**Supplemental** [Figure 20](#figur_Overlap_pathways) **| Extended version of Figure 5 from the main text (pathways).** Extended version where all enriched pathways are shown (irrespective of them being very general or redundant) . Additionally, label names are printed out in full. The number of genes in each set is indicated in squared brackets.


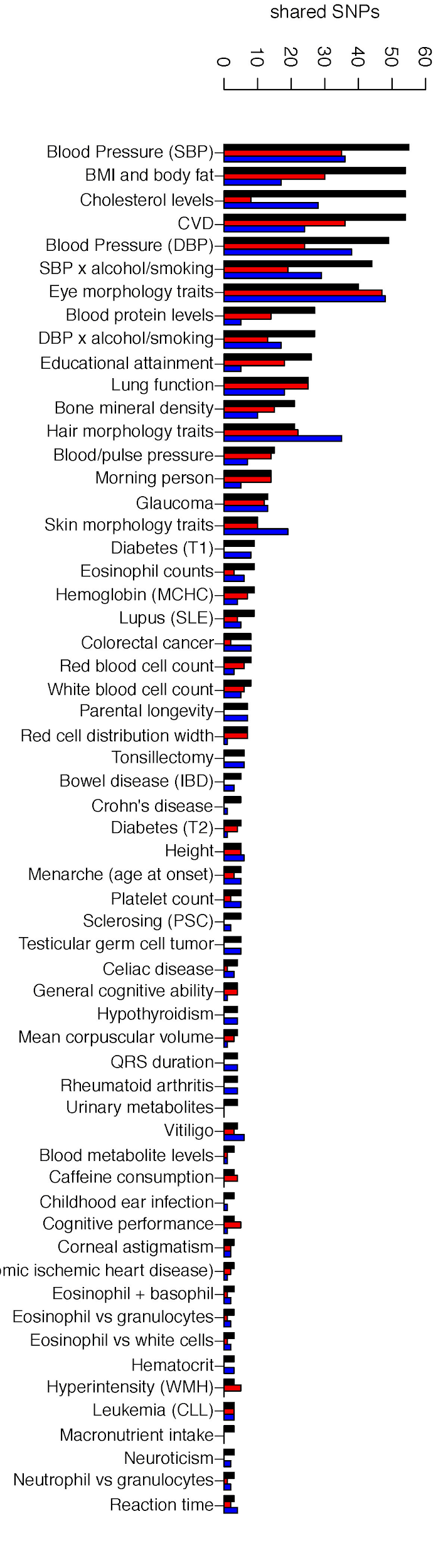


**Supplemental** [Figure 21](#figur_Overlap_signals_diseases) **| Extended version of Figure 4 from the main text.** Extended version where all traits with at least 3 shared associations are included. For full data, refer to Supplemental Dataset 3.

**List of label replacements**

Supplemental [Figure 21](#fig_Overlap_signals_diseases) was generated based on data in Supplemental Dataset 3. The following replacements were applied to the column "Trait" to homogenize or shorten some labels.

"Blood pressure"<-"Blood/pulse pressure"

"Pulse pressure"<-"Blood/pulse pressure"

"Systolic blood pressure"<-"Blood Pressure (SBP)"

"Mean arterial pressure"<-"Blood Pressure (SBP)"

"Diastolic blood pressure"<-"Blood Pressure (DBP)"

"Blood pressure traits (multi-trait analysis)"<-"Blood Pressure (DBP)"

"Body mass index"<-"BMI and body fat"

"Body fat percentage"<-"BMI and body fat"

"Fat-free mass"<-"BMI and body fat"

"Body mass index (joint analysis main effects and physical activity interaction)"<-"BMI and body fat"

"BMI and body fat (joint analysis main effects and physical activity interaction)"<-"BMI and body fat"

"Waist-to-hip ratio adjusted for BMI (additive genetic model)"<-"BMI and body fat"

"Waist-hip ratio"<-"BMI and body fat"

"Hip circumference adjusted for BMI"<-"BMI and body fat"

"Obesity-related traits"<-"BMI and body fat"

"Triglycerides"<-"Cholesterol levels"

"Cholesterol, total"<-"Cholesterol levels"

"LDL cholesterol"<-"Cholesterol levels"

"High density lipoprotein cholesterol levels"<-"Cholesterol levels"

"Low density lipoprotein cholesterol levels"<-"Cholesterol levels"

"Total cholesterol levels"<-"Cholesterol levels"

"HDL cholesterol"<-"Cholesterol levels"

"C-reactive protein levels or LDL-cholesterol levels (pleiotropy)"<-"Cholesterol levels"

"Coronary artery disease (myocardial infarction, percutaneous transluminal coronary angioplasty, coronary artery bypass grafting, angina or chronic ischemic heart disease)"<-"CVD"

"Myocardial infarction (early onset)"<-"CVD"

"Coronary artery disease or ischemic stroke"<-"CVD"

"Coronary artery disease or large artery stroke"<-"CVD"

"Cardiovascular disease"<-"CVD"

"Ischemic stroke"<-"CVD"

"Stroke"<-"CVD"

"Stroke (large artery atherosclerosis)"<-"CVD"

"Stroke (small-vessel)"<-"CVD"

"Myocardial infarction"<-"CVD"

"Ischemic stroke (small-vessel)"<-"CVD"

"Coronary artery disease"<-"CVD"

"Coronary heart disease"<-"CVD"

"CVD (small-vessel)"<-"CVD"

"Type 1 diabetes"<-"Diabetes (T1)"

"Latent autoimmune diabetes vs. type 2 diabetes"<-"Diabetes (T1)"

"Latent autoimmune diabetes vs. type 2 diabetes"<-"Diabetes (T2)"

"Type 2 diabetes"<-"Diabetes (T2)"

"Intraocular pressure"<-"Eye morphology traits"

"Macular thickness"<-"Eye morphology traits"

"Vertical cup-disc ratio"<-"Eye morphology traits"

"Optic cup area"<-"Eye morphology traits"

"Optic disc area"<-"Eye morphology traits"

"Eye color traits"<-"Eye morphology traits"

"Eye color"<-"Eye morphology traits"

"Eye color (brightness)"<-"Eye morphology traits"

"Eye color (hue)"<-"Eye morphology traits"

"Retinal vascular caliber"<-"Eye morphology traits"

"Eye color (saturation)"<-"Eye morphology traits"

"Blue vs. green eyes"<-"Eye morphology traits"

"Blue vs. brown eyes"<-"Eye morphology traits"

"Optic disc parameters"<-"Eye morphology traits"

"Optic disc parameters"<-"Hair morphology traits"

"Hair color"<-"Hair morphology traits"

"Hair morphology traits"<-"Hair morphology traits"

"Blond vs. brown/black hair color"<-"Hair morphology traits"

"Black vs. blond hair color"<-"Hair morphology traits"

"Black vs. red hair color"<-"Hair morphology traits"

"Blond vs. brown hair color"<-"Hair morphology traits"

"Brown vs. black hair color"<-"Hair morphology traits"

"Red vs. brown/black hair color"<-"Hair morphology traits"

"Skin pigmentation traits"<-"Skin morphology traits"

"Skin pigmentation"<-"Skin morphology traits"

"Perceived skin darkness"<-"Skin morphology traits"

"Glaucoma (primary open-angle)"<-"Glaucoma"

"Glaucoma (high intraocular pressure)"<-"Glaucoma"

"Lung function (FEV1/FVC)"<-"Lung function"

"Lung function (FEV1)"<-"Lung function"

"Post bronchodilator FEV1/FVC ratio"<-"Lung function"

"FEV1"<-"Lung function"

"Peak expiratory flow"<-"Lung function"

"Lung function (FVC)"<-"Lung function"

"Lung function (Lung function)"<-"Lung function"

"Mean arterial pressure x alcohol consumption (light vs heavy) interaction (2df test)"<-"SBP x alcohol/smoking"

"Mean arterial pressure x alcohol consumption interaction (2df test)"<-"SBP x alcohol/smoking"

"Blood Pressure (SBP) x alcohol consumption interaction (2df test)"<-"SBP x alcohol/smoking"

"Systolic blood pressure (alcohol consumption interaction)"<-"SBP x alcohol/smoking"

"Blood Pressure (DBP) x alcohol consumption interaction (2df test)"<-"DBP x alcohol/smoking"

"Diastolic blood pressure x smoking status (current vs non-current) interaction (2df test)"<-"DBP x alcohol/smoking"

"Diastolic blood pressure x smoking status (ever vs never) interaction (2df test)"<-"DBP x alcohol/smoking"

"Blood Pressure (SBP) x alcohol consumption (light vs heavy) interaction (2df test)"<-"SBP x alcohol/smoking"

"Blood Pressure (DBP) x alcohol consumption (light vs heavy) interaction (2df test)"<-"DBP x alcohol/smoking"

"Blood Pressure (SBP) (cigarette smoking interaction)"<-"SBP x alcohol/smoking"

"Blood Pressure (DBP) (cigarette smoking interaction)"<-"DBP x alcohol/smoking"

"Systolic blood pressure x alcohol consumption interaction (2df test)"<-"SBP x alcohol/smoking"

"Diastolic blood pressure x alcohol consumption interaction (2df test)"<-"DBP x alcohol/smoking"

"Systolic blood pressure x alcohol consumption (light vs heavy) interaction (2df test)"<-"SBP x alcohol/smoking"

"Diastolic blood pressure x alcohol consumption (light vs heavy) interaction (2df test)"<-"DBP x alcohol/smoking"

"Systolic blood pressure (cigarette smoking interaction)"<-"SBP x alcohol/smoking"

"Diastolic blood pressure (cigarette smoking interaction)"<-"DBP x alcohol/smoking"

"Educational attainment (MTAG)"<-"Educational attainment"

"Highest math class taken (MTAG)"<-"Educational attainment"

"Cognitive performance (MTAG)"<-"Cognitive performance"

"Parental longevity (combined parental attained age, Martingale residuals)"<-"Parental longevity"

"Educational attainment (years of education)"<-"Educational attainment"

"Alcohol consumption (drinks per week)"<-"Alcohol (drinks/week)"

"Mean corpuscular hemoglobin"<-"Hemoglobin (MCHC)"

"Mean corpuscular hemoglobin concentration"<-"Hemoglobin (MCHC)"

"Systemic lupus erythematosus"<-"Lupus (SLE)"

"Morning vs. evening chronotype"<-"Morning person"

"Chronotype"<-"Morning person"

"Primary sclerosing cholangitis"<-"Sclerosing (PSC)"

"Urinary metabolites (H-NMR features)"<-"Urinary metabolites"

"Heel bone mineral density"<-"Bone mineral density"

"Chronic lymphocytic leukemia"<-"Leukemia (CLL)"

"Dietary macronutrient intake"<-"Macronutrient intake"

"Eosinophil percentage of granulocytes"<-"Eosinophil vs granulocytes"

"Eosinophil percentage of white cells"<-"Eosinophil vs white cells"

"Neutrophil percentage of granulocytes"<-"Neutrophil vs granulocytes"

"Sum eosinophil basophil counts"<-"Eosinophil + basophil"

"White matter hyperintensity burden"<-"Hyperintensity (WMH)"

"Inflammatory bowel disease"<-"Bowel disease (IBD)"

## Text 14: Dependency of GWAS on covariates selection

Previous analysis have been computed using as covariants age, sex, and PC of the genotypes (we considered only PCs with a significant correlation to tortuosity, namely 1, 2, 5, 6, 7, 8, 16, 17 and 18). Since these are the basics ones, in this section we analyze how the results changes at the level of SNPs and genes, when adding also other covariates. Particularly: a) age^2, since due to its high influence in tortuosity a linear term might not be enough, b) the spherical power, to correct for eye physical measures, c) hypertension (specifically, SBD and DBP), and d) diabetes since they are known to has clinical associations with vascular features, e) smoking information, since it is known that it has secondary effects on the vasculature throughout the body, f) ocular diseases (the ones reported in the supplemental [Table 3](#table_baseline_diseases)), g) assessment-center attended, and h) genotyping array used. Scatterplots between the p-values of the tortuosity GWAS adding these covariates to the original ones against the p-values of the tortuosity GWAS of the original covariates can be found in supplemental figur[e 22](#figur_snp_scatterplots_cov). According to these results, correcting for these covariates did not substantially alter the p-values for any of the significant SNP-wise associations. Although, using spherical power equivalent as covariate had the highest impact on our SNP p-values. This correction reduced significance, at least in part because this information was missing for a subset of subjects, resulting in less power. Nevertheless, all top-hits remain highly significant.

a) b)

**
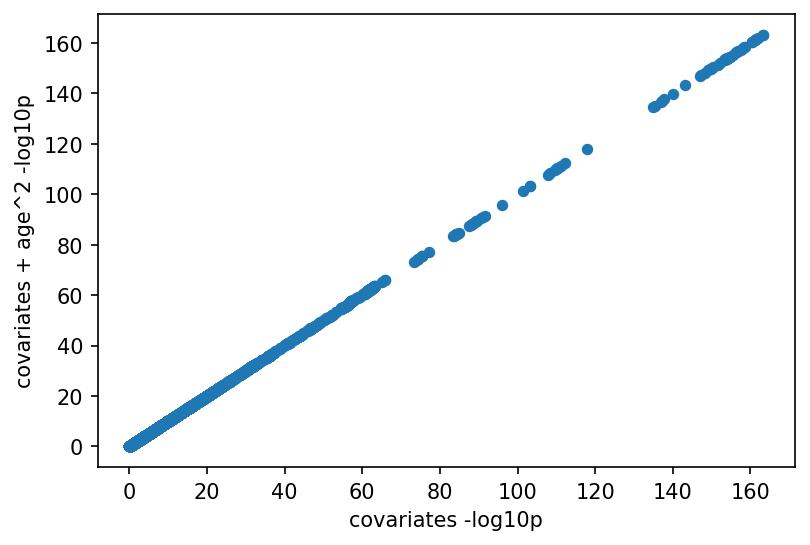

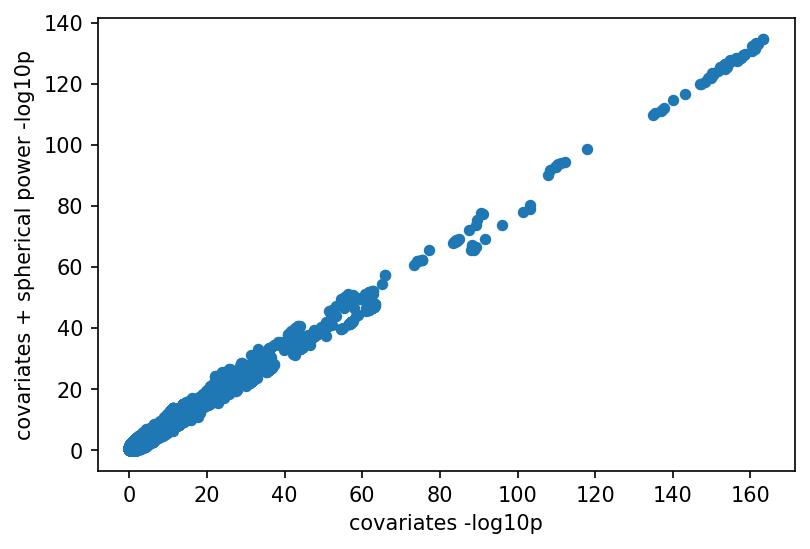
**

c) d)

**
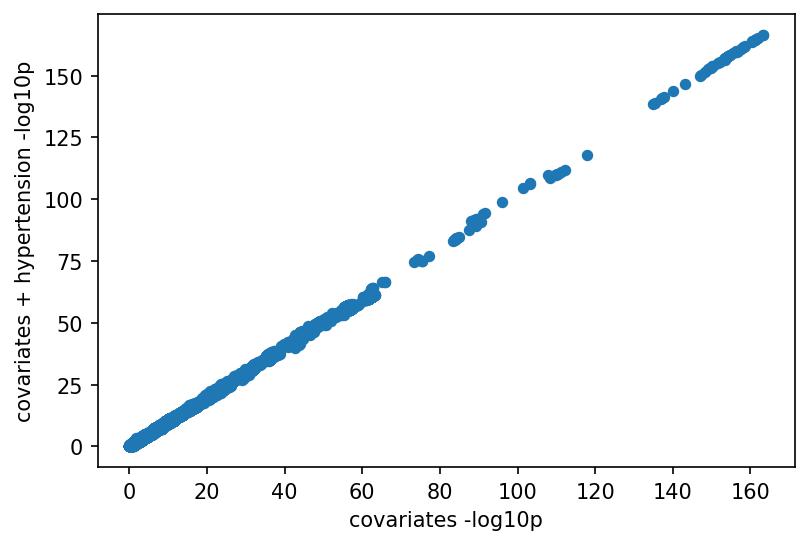
**
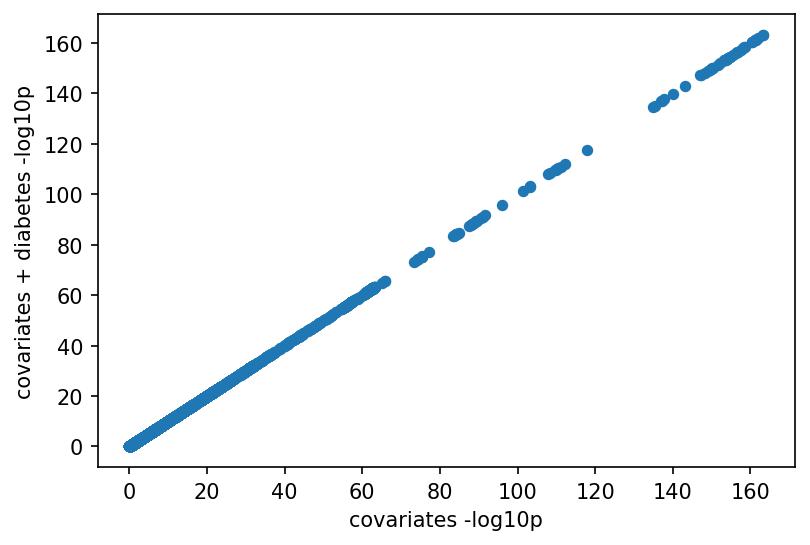


e) f)

**
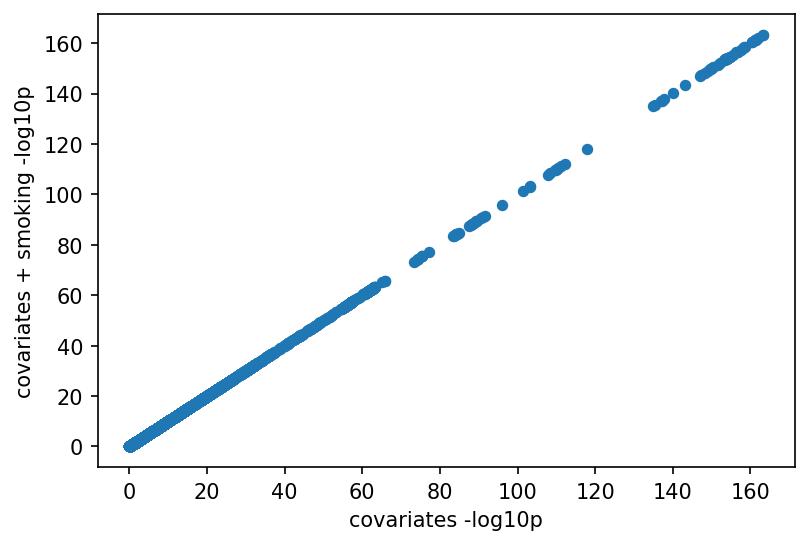

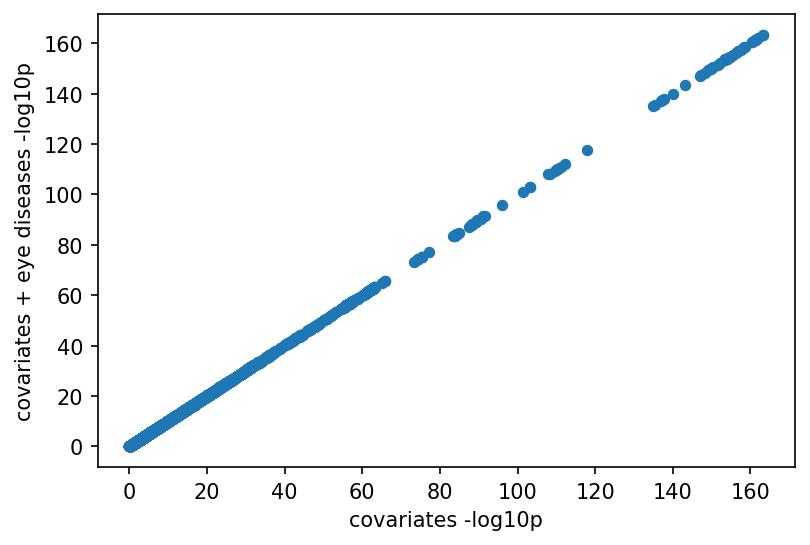
**

g**)**  h)


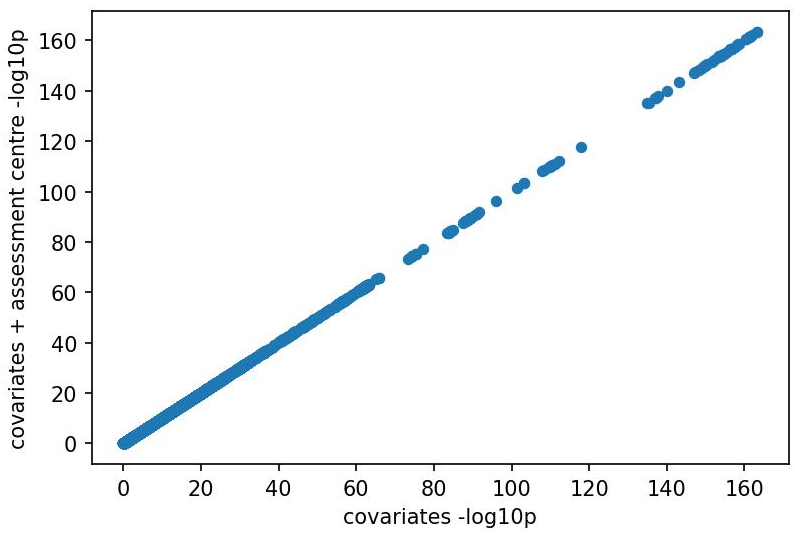

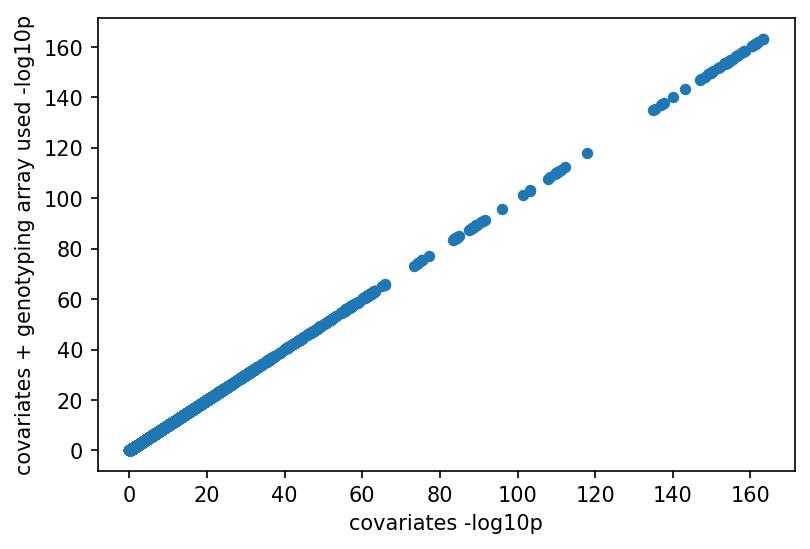


**Supplemental** [Figure 22](#figur_snp_scatterplots_cov) **|** SNPs scatter plots between -log10 P-values of the original GWAS and the -log10 P-values of the GWAS correcting for additional covariates.

**References**

1. [Bankhead P, Scholfield CN, McGeown JG, Curtis TM. Fast retinal vessel detection and measurement using wavelets and edge location refinement. PLoS One. 2012;7: e32435.](http://paperpile.com/b/4pBVkZ/9XWqH)

2. [Galdran A, Anjos A, Dolz J, Chakor H, Lombaert H, Ayed IB. The Little W-Net That Could: State-of-the-Art Retinal Vessel Segmentation with Minimalistic Models. 2020. Available:](http://paperpile.com/b/4pBVkZ/kcuJc) <http://arxiv.org/abs/2009.01907>

3. [Lee CH, Eskin E, Han B. Increasing the power of meta-analysis of genome-wide association studies to detect heterogeneous effects. Bioinformatics. 2017;33: i379–i388.](http://paperpile.com/b/4pBVkZ/cB7X)

4. [Veluchamy A, Ballerini L, Vitart V, Schraut KE, Kirin M, Campbell H, et al. Novel Genetic Locus Influencing Retinal Venular Tortuosity Is Also Associated With Risk of Coronary Artery Disease. Arterioscler Thromb Vasc Biol. 2019;39: 2542–2552.](http://paperpile.com/b/4pBVkZ/Ttc9g)

5. [Schunkert H, König IR, Kathiresan S, Reilly MP, Assimes TL, Holm H, et al. Large-scale association analysis identifies 13 new susceptibility loci for coronary artery disease. Nat Genet. 2011;43: 333–338.](http://paperpile.com/b/4pBVkZ/d4FtO)

6. [C4D Consortium. A genome-wide association study in Europeans and South Asians identifies five new loci for coronary artery disease. Nat Genet. 2011;43: 339–344.](http://paperpile.com/b/4pBVkZ/Xbhsn)

7. [Osman W, Low S-K, Takahashi A, Kubo M, Nakamura Y. A genome-wide association study in the Japanese population confirms 9p21 and 14q23 as susceptibility loci for primary open angle glaucoma. Hum Mol Genet. 2012;21: 2836–2842.](http://paperpile.com/b/4pBVkZ/tidye)

8. [Craig JE, Han X, Qassim A, Hassall M, Cooke Bailey JN, Kinzy TG, et al. Multitrait analysis of glaucoma identifies new risk loci and enables polygenic prediction of disease susceptibility and progression. Nat Genet. 2020;52: 160–166.](http://paperpile.com/b/4pBVkZ/ykYon)

9. [German CA, Sinsheimer JS, Klimentidis YC, Zhou H, Zhou JJ. Ordered multinomial regression for genetic association analysis of ordinal phenotypes at Biobank scale. Genet Epidemiol. 2020;44: 248–260.](http://paperpile.com/b/4pBVkZ/s22tj)

10. [Barrett JC, Clayton DG, Concannon P, Akolkar B, Cooper JD, Erlich HA, et al. Genome-wide association study and meta-analysis find that over 40 loci affect risk of type 1 diabetes. Nat Genet. 2009;41: 703–707.](http://paperpile.com/b/4pBVkZ/gNnhZ)

11. [Plagnol V, Howson JMM, Smyth DJ, Walker N, Hafler JP, Wallace C, et al. Genome-wide association analysis of autoantibody positivity in type 1 diabetes cases. PLoS Genet. 2011;7: e1002216.](http://paperpile.com/b/4pBVkZ/4rhsL)

12. [Shah S, Henry A, Roselli C, Lin H, Sveinbjörnsson G, Fatemifar G, et al. Genome-wide association and Mendelian randomisation analysis provide insights into the pathogenesis of heart failure. Nat Commun. 2020;11: 163.](http://paperpile.com/b/4pBVkZ/x7QKM)

13. [Dubois PCA, Trynka G, Franke L, Hunt KA, Romanos J, Curtotti A, et al. Multiple common variants for celiac disease influencing immune gene expression. Nat Genet. 2010;42: 295–302.](http://paperpile.com/b/4pBVkZ/ec8Mm)

14. [Jin Y, Birlea SA, Fain PR, Ferrara TM, Ben S, Riccardi SL, et al. Genome-wide association analyses identify 13 new susceptibility loci for generalized vitiligo. Nat Genet. 2012;44: 676–680.](http://paperpile.com/b/4pBVkZ/33U5Y)

15. [Kichaev G, Bhatia G, Loh P-R, Gazal S, Burch K, Freund MK, et al. Leveraging Polygenic Functional Enrichment to Improve GWAS Power. Am J Hum Genet. 2019;104: 65–75.](http://paperpile.com/b/4pBVkZ/3iVgy)

16. [Comuzzie AG, Cole SA, Laston SL, Voruganti VS, Haack K, Gibbs RA, et al. Novel genetic loci identified for the pathophysiology of childhood obesity in the Hispanic population. PLoS One. 2012;7: e51954.](http://paperpile.com/b/4pBVkZ/ropyf)

17. [Levy D, Ehret GB, Rice K, Verwoert GC, Launer LJ, Dehghan A, et al. Genome-wide association study of blood pressure and hypertension. Nat Genet. 2009;41: 677–687.](http://paperpile.com/b/4pBVkZ/i8qhg)

18. [Giri A, Hellwege JN, Keaton JM, Park J, Qiu C, Warren HR, et al. Trans-ethnic association study of blood pressure determinants in over 750,000 individuals. Nat Genet. 2019;51: 51–62.](http://paperpile.com/b/4pBVkZ/iDWJd)

19. [Newton-Cheh C, Johnson T, Gateva V, Tobin MD, Bochud M, Coin L, et al. Genome-wide association study identifies eight loci associated with blood pressure. Nat Genet. 2009;41: 666–676.](http://paperpile.com/b/4pBVkZ/rDOwW)

20. [Wain LV, Verwoert GC, O’Reilly PF, Shi G, Johnson T, Johnson AD, et al. Genome-wide association study identifies six new loci influencing pulse pressure and mean arterial pressure. Nat Genet. 2011;43: 1005–1011.](http://paperpile.com/b/4pBVkZ/edvlO)

21. [Davies G, Lam M, Harris SE, Trampush JW, Luciano M, Hill WD, et al. Study of 300,486 individuals identifies 148 independent genetic loci influencing general cognitive function. Nat Commun. 2018;9: 2098.](http://paperpile.com/b/4pBVkZ/isYSy)

22. [Krefl D, Bergmann S. PascalX v0.0.1. 2021. doi:](http://paperpile.com/b/4pBVkZ/B1ndF)[10.5281/zenodo.4429922](http://dx.doi.org/10.5281/zenodo.4429922)

23. [Lonsdale J, Thomas J, Salvatore M, Phillips R, Lo E, Shad S, et al. The Genotype-Tissue Expression (GTEx) project. Nat Genet. 2013;45: 580–585.](http://paperpile.com/b/4pBVkZ/I081K)

24. [Safran M, Dalah I, Alexander J, Rosen N, Iny Stein T, Shmoish M, et al. GeneCards Version 3: the human gene integrator. Database . 2010;2010: baq020.](http://paperpile.com/b/4pBVkZ/qOyc)

25. [Szklarczyk D, Gable AL, Lyon D, Junge A, Wyder S, Huerta-Cepas J, et al. STRING v11: protein–protein association networks with increased coverage, supporting functional discovery in genome-wide experimental datasets. Nucleic Acids Res. 2018;47: D607–D613.](http://paperpile.com/b/4pBVkZ/kJEr)
